# *N*-Succinyltransferase Encoded by a Cryptic Siderophore Biosynthesis Gene Cluster in *Streptomyces* Modifies Structurally Distinct Antibiotics

**DOI:** 10.1128/mbio.01789-22

**Published:** 2022-08-30

**Authors:** Olha Schneider, Martin Zehl, Reiko Ueoka, Christian Rückert, Tobias Buche, Yi Jiang, Qin-Yuan Li, Jörn Piel, Sergey B. Zotchev

**Affiliations:** a Department of Pharmaceutical Sciences, Division of Pharmacognosy, University of Vienna, Vienna, Austria; b Department of Analytical Chemistry, Faculty of Chemistry, University of Vienna, Vienna, Austria; c Department of Biology, Eidgenössische Technische Hochschule (ETH) Zurich, Zurich, Switzerland; d Center for Biotechnology, Bielefeld University, Bielefeld, Germany; e Yunnan Institute of Microbiology, Yunnan University, Kunming, People’s Republic of China; f Life Sciences Lab Center, School of Life Sciences, Yunnan University, Kunming, People’s Republic of China; Mass General Hospital

**Keywords:** *Streptomyces*, desertomycin analogue, succinyltransferase, antibiotic resistance, *N*-succinyltransferase

## Abstract

The antibiotic desertomycin A and its previously undescribed inactive *N*-succinylated analogue, desertomycin X, were isolated from *Streptomyces* sp. strain YIM 121038. Genome sequencing and analysis readily identified the desertomycin biosynthetic gene cluster (BGC), which lacked genes encoding acyltransferases that would account for desertomycin X formation. Scouting the genome for putative *N-*acyltransferase genes led to the identification of a candidate within a cryptic siderophore BGC (*csb*) encoding a putative homologue of the *N*6′-hydroxylysine acetyltransferase IucB. Expression of the codon-optimized gene designated *csbC* in Escherichia coli yielded the recombinant protein that was able to *N*-succinylate desertomycin A as well as several other structurally distinct antibiotics harboring amino groups. Some antibiotics were rendered antibiotically inactive due to the CsbC-catalyzed succinylation *in vitro*. Unlike many known *N*-acyltransferases involved in antibiotic resistance, CsbC could not efficiently acetylate the same antibiotics. When expressed in E. coli, CsbC provided low-level resistance to kanamycin and ampicillin, suggesting that it may play a role in antibiotic resistance in natural habitats, where the concentration of antibiotics is usually low.

## INTRODUCTION

Antibiotics are secondary metabolites (SMs) produced by certain bacteria and fungi, which are not required for growth, development, or reproduction, but may provide an advantage in competition for nutrients or defense against predators in natural environments. Genes encoding enzymes for the biosynthesis of SMs are usually colocalized in the genome and form so-called “biosynthetic gene clusters” (BGCs). Typical BGCs contain “core” genes responsible for the biosynthesis of a molecular scaffold, accessory genes encoding enzymes for scaffold modification, genes that regulate the BGC’s expression, and resistance genes for self-protection against the endogenously produced compound (1). Actinomycetes, filamentous Gram-positive bacteria of the phylum *Actinobacteria*, produce thousands of SMs, including ca 50% of all clinically used antibiotics (2). Actinomycetes protect themselves from the toxic effects of endogenously produced antibiotics using a variety of specific mechanisms (3). For example, the ErmE protein of the erythromycin producer Saccharopolyspora erythraea catalyzes methylation of 23S rRNA, thus preventing binding of erythromycin to the ribosome and inhibiting protein synthesis (4). The tetracycline producer Streptomyces rimosus encodes, within the tetracycline BGC, a transmembrane protein channel that pumps the antibiotic out of the cells by coupling antibiotic excretion with proton import (5, 6). Other prevalent examples of resistance mechanisms are the direct enzymatic modification of antibiotics via hydrolysis or transfer of a specific chemical group that prevents an antibiotic from binding to its target. Despite the structural diversity of antibiotics, two modes of the latter modification predominate: *N*-acetylation of amino groups and *O*-phosphorylation of hydroxyl groups, for which the donor cofactors are acyl coenzyme A (acyl-CoA) and ATP, respectively (6). *N*-Acetylation of amino groups is catalyzed by GNAT enzymes (Gcn5-related *N*-acetyltransferases), most of which use acetyl-CoA as a cosubstrate (7). However, GNAT proteins exist that prefer different acyl donors, such as myristoyl-CoA in Saccharomyces cerevisiae (8) or succinyl-CoA in Mycobacterium tuberculosis (9). The latter protein was shown to both acylate and succinylate mycobacterial nucleoid-associated protein HU (10). The involvement of *N*-acetyltransferases in antibiotic resistance first was shown in the kanamycin producer Streptomyces kanamyceticus, which possesses an aminoglycoside *N*-acetyltransferase (AAC) activity (11). The *aac* gene (new designation *kanM*) encoding the AAC enzyme has been cloned in Streptomyces lividans, where it functioned as a resistance determinant against kanamycin (12, 13). Except for the aforementioned KanM enzyme from *S. kanamyceticus*, not much information is available about the AAC proteins from *Streptomyces.*

Here, we describe the characterization of a GNAT-like protein, CsbC, encoded by a BGC for a cryptic siderophore in the newly isolated strain *Streptomyces* YIM 121038. We demonstrate *N*-succinyltransferase activity of CsbC on a range of structurally distinct antibiotics, some of which completely lose their activity due to such modification. The latter suggests that proteins similar to CsbC may contribute to antibiotic resistance in nature, providing an additional line of defense against competitors capable of antibiotic biosynthesis.

## RESULTS

### Isolation and genome analyses of *Streptomyces* sp. strain YIM 121038.

The actinobacterial strain YIM 121038 was isolated from a rain forest soil sample collected in the Province Yunnan, China. Its 16S rRNA gene sequence was deposited in NCBI (accession no. NCBI:txid2136401). According to its morphological and physiological properties and the 16S rRNA BLAST results, the YIM 121038 isolate was identified as a strain of *Streptomyces* sp. The 16S rRNA gene sequence similarity between the strain YIM121038 and Streptomyces spectabilis was 98.43%.

The genome of *Streptomyces* sp. strain YIM 121038 was sequenced and shown to contain one linear chromosome of 10,130,554 bp and a linear plasmid of 967,890 bp. *In silico* analysis of the draft genome for the presence of biosynthetic gene clusters (BGCs) using antiSMASH 6.0 (14) revealed >50 putative clusters for secondary metabolite biosynthesis (see Table S1 in the supplemental material). The chromosome of YIM 121038 harbored 42 BGCs, several of which could be associated with a high degree of certainty with known secondary metabolites. Most of the BGCs detected on the YIM 121038 chromosome (29 out of 42) had very close homologues in the genome of *Streptomyces* sp. strain MNP-20 and *Streptomyces* sp. strain NRRL B-1347. The linear plasmid of almost 1 Mb in size was found to harbor 11 BGCs (Table S1). The BLAST analysis of single genes within the clusters suggested their unique origin, since no plausible hits could be found in the databases.

10.1128/mbio.01789-22.6 TABLE S1 Secondary metabolite biosynthetic gene clusters in the genome of *Streptomyces* sp. strain YIM 121038 predicted with antiSMASH 6.0 followed by manual curation. Shaded cells represent BGCs identified on the 1-Mb linear plasmid. Download Table S1, DOCX file, 0.02 MB. Copyright © 2022 Schneider et al. 2022 Schneider et al. https://creativecommons.org/licenses/by/4.0/ This content is distributed under the terms of the Creative Commons Attribution 4.0 International license.

### Identification of desertomycin A and its succinylated congener.

**FIG 1:**
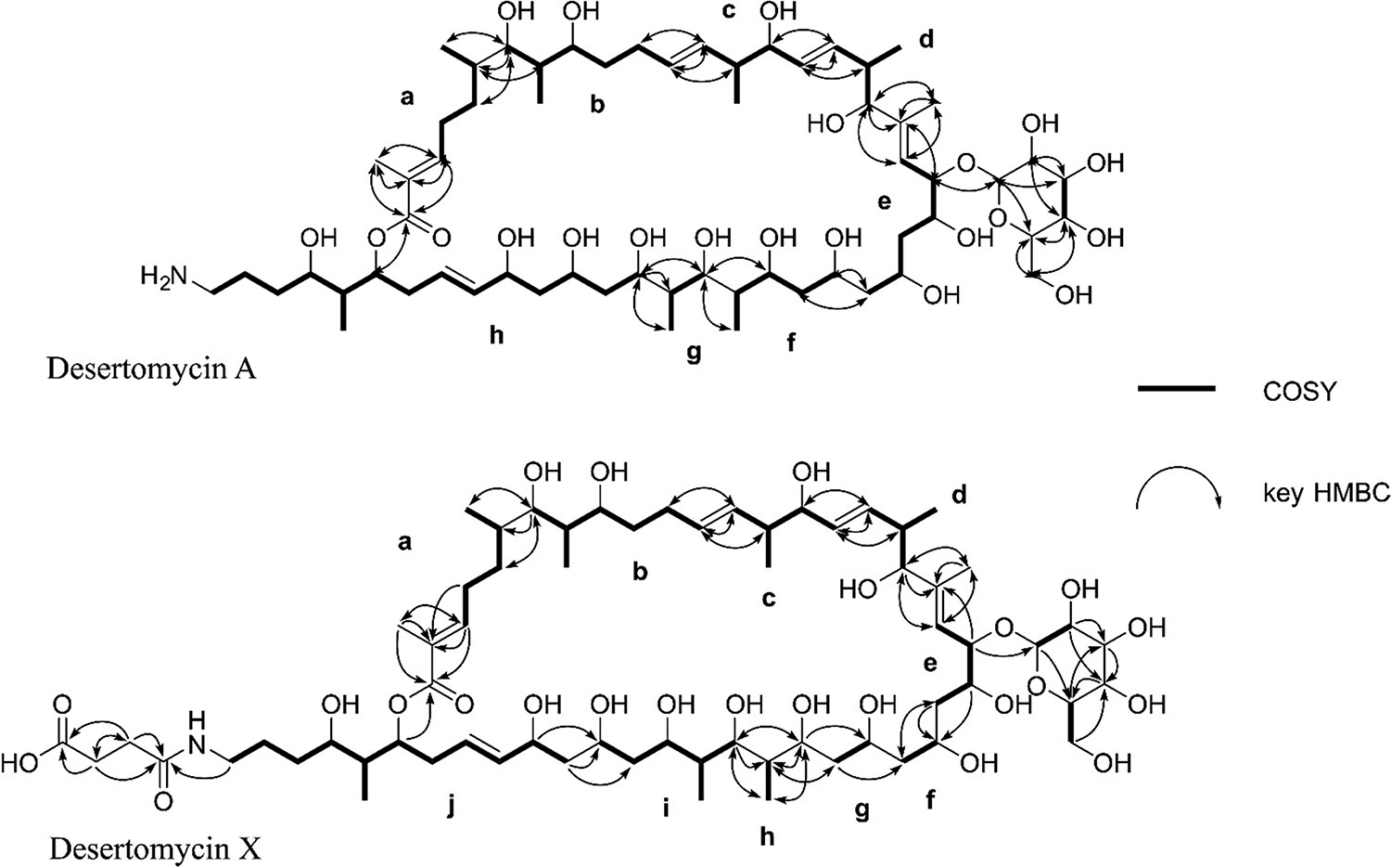
Chemical structures of desertomycin A and its new derivative, desertomycin X, determined by NMR spectroscopy.

The methanolic mycelial extracts from the culture of *Streptomyces* sp. strain YIM 121038 exhibited antibacterial activity. To identify the compound(s) responsible for this activity, the extract was subjected to repetitive reversed-phase high-performance liquid chromatography (HPLC) to afford two compounds associated with fractions containing antibacterial activity, one of which had a predicted molecular formula of C_65_H_113_O_24_N as determined by high-resolution electrospray ionization mass spectrometry (HR-ESIMS) (Fig. S1A). This compound was subjected to structure elucidation by means of nuclear magnetic resonance (NMR). The ^1^H NMR and heteronuclear single quantum coherence (HSQC) spectra suggested that there were 7 doublet methyls, two singlet methyls, 8 protons attached to sp^2^ carbons, and many oxygenated methines (Fig. S1B to E). Correlation spectroscopy (COSY) correlations showed 10 units, a to j (Fig. 1; Fig. S2F and G). heteronuclear multiple-bond correlations (HMBCs) unambiguously revealed the mode of connections of those units (Fig. 1A; Fig. S1H and I). The sugar moiety was attached to C-22, which was determined by HMBCs from H-22 to C-1′. The presence of 46-NH was deduced by COSY correlations between H-46 and 46-NH measured in dimethyl sulfoxide (DMSO)-*d*_6_ (Fig. S1G). Finally, the succinyl moiety was identified by HMBCs from H-2″/3″ to C-1″/C-4″ and shown to be attached to the N terminus of unit j by an HMBC from H-46 to C-1″. The 38E geometry was determined by the large coupling constant (^3^*J*_H38,H39_ = 15.7 Hz). The 2E, 12E, 16E, and 20E geometries were elucidated by nuclear Overhauser effect spectroscopy (NOESY) correlations (Fig. S1J). Compound 1 was found to be identical to the known antibiotic desertomycin A (15), except for the *N*-succinyl moiety, and was named desertomycin X.

10.1128/mbio.01789-22.1 FIG S1 (A) HR-LC-ESIMS data of desertomycin X (compound 1). Shown is the mass spectrum of the peak at 13.66 min (*m*/*z* 1,292.7739 [M + H]^+^, *m*/*z* 1,274.7635 [M + H-H_2_O]^+^, *m*/*z* 1,256.7534 [M + H-2H_2_O]^+^, *m*/*z* 1,309.8000 [M+NH_4_]^+^, and *m*/*z* 1314.7545 [M+Na]^+^). (B) ^1^H NMR spectrum of desertomycin X in methanol-*d*_4_ at 500 MHz; (C) ^1^H NMR spectrum of desertomycin X in DMSO-*d*_6_; (D) HSQC spectrum of desertomycin X in methanol-*d*_4_ at 500 MHz; (E) HSQC spectrum of desertomycin X in DMSO-*d*_6_; (F) COSY spectrum of desertomycin X in methanol-*d*_4_ at 500 MHz; (G) COSY spectrum of desertomycin X in DMSO-*d*_6_; (H) HMBC spectrum of desertomycin X in methanol-*d*_4_ at 500 MHz; (I) HMBC spectrum of desertomycin X in DMSO-*d*_6_; (J) NOESY spectrum of desertomycin X in methanol-*d*_4_ at 500 MHz. Download FIG S1, PDF file, 0.4 MB. Copyright © 2022 Schneider et al. 2022 Schneider et al. https://creativecommons.org/licenses/by/4.0/ This content is distributed under the terms of the Creative Commons Attribution 4.0 International license.

10.1128/mbio.01789-22.2 FIG S2 (A) HR-LC-ESIMS data of desertomycin A (compound 2). Shown is the mass spectrum of the peak at 12.87 min (*m*/*z* 1,192.7561 [M + H]^+^ and *m*/*z* 1,214.7377 [M+Na]^+^). (B) ^1^H NMR spectrum of desertomycin A in methanol-*d*_4_ at 500 MHz; (C) HSQC spectrum of desertomycin A in methanol-*d*_4_ at 500 MHz; (D) COSY spectrum of desertomycin A in methanol-*d*_4_ at 600 MHz; (E) HMBC spectrum of desertomycin A in methanol-*d*_4_ at 600 MHz; (F) NOESY spectrum of desertomycin A in methanol-*d*_4_ at 600 MHz. Download FIG S2, PDF file, 0.3 MB. Copyright © 2022 Schneider et al. 2022 Schneider et al. https://creativecommons.org/licenses/by/4.0/ This content is distributed under the terms of the Creative Commons Attribution 4.0 International license.

The second compound (compound 2) had a predicted molecular formula of C_61_H_109_O_21_N, which was determined by HR-ESIMS (Fig. S2A). The ^1^H NMR spectrum of this compound suggested that the signals at the lower magnetic field were nearly identical to those for compound 1 (Fig. S2B), while the HSQC spectrum suggested the absence of the *N*-succinyl moiety compared to compound 1 (Fig. S2C). The detailed analysis of the two-dimensional (2D) NMR data, including COSY, HMBC, and NOESY spectra, confirmed the structure of compound 2 as that of desertomycin A (Fig. S2D to F). The chemical structures of the two desertomycin congeners are shown in Fig. 1.

### Antimicrobial activity of the isolated compounds.

To test for antimicrobial activity, purified desertomycin A and desertomycin X were initially tested on Gram-positive and Gram-negative bacteria, as well as yeast and filamentous fungi, using a disk assay (see Materials and Methods for details). While desertomycin A inhibited the growth of both Bacillus subtilis and Escherichia coli (data not shown), desertomycin X was antibiotically inactive. This observation suggested that the succinyl group attached to the primary amine is detrimental for the antibacterial activity of desertomycin. In order to properly assess antibiotic activity of desertomycin A, it was tested, along with some clinically relevant antibiotics, on a panel of Gram-positive and Gram-negative bacteria using the broth microdilution method. This test did not reveal any measurable activity of desertomycin A against Gram-negative bacteria, including E. coli, at concentrations below 128 μg/mL. This can be explained by different physiological conditions of bacterial cultures on the solid agar medium (initial disk assay) and in liquid cultures (CLSI method). At the same time, desertomycin A exhibited moderate activity against all of the Gram-positive bacteria tested, including Staphylococcus aureus, Enterococcus faecium, and Streptococcus mutans (Table S2).

10.1128/mbio.01789-22.7 TABLE S2 Antibacterial activity of desertomycin A and desertomycin X against Gram-positive bacteria. Download Table S2, DOCX file, 0.01 MB. Copyright © 2022 Schneider et al. 2022 Schneider et al. https://creativecommons.org/licenses/by/4.0/ This content is distributed under the terms of the Creative Commons Attribution 4.0 International license.

### Identification and characterization of CsbC as an *N*-succinyltransferase.

**FIG 2:**
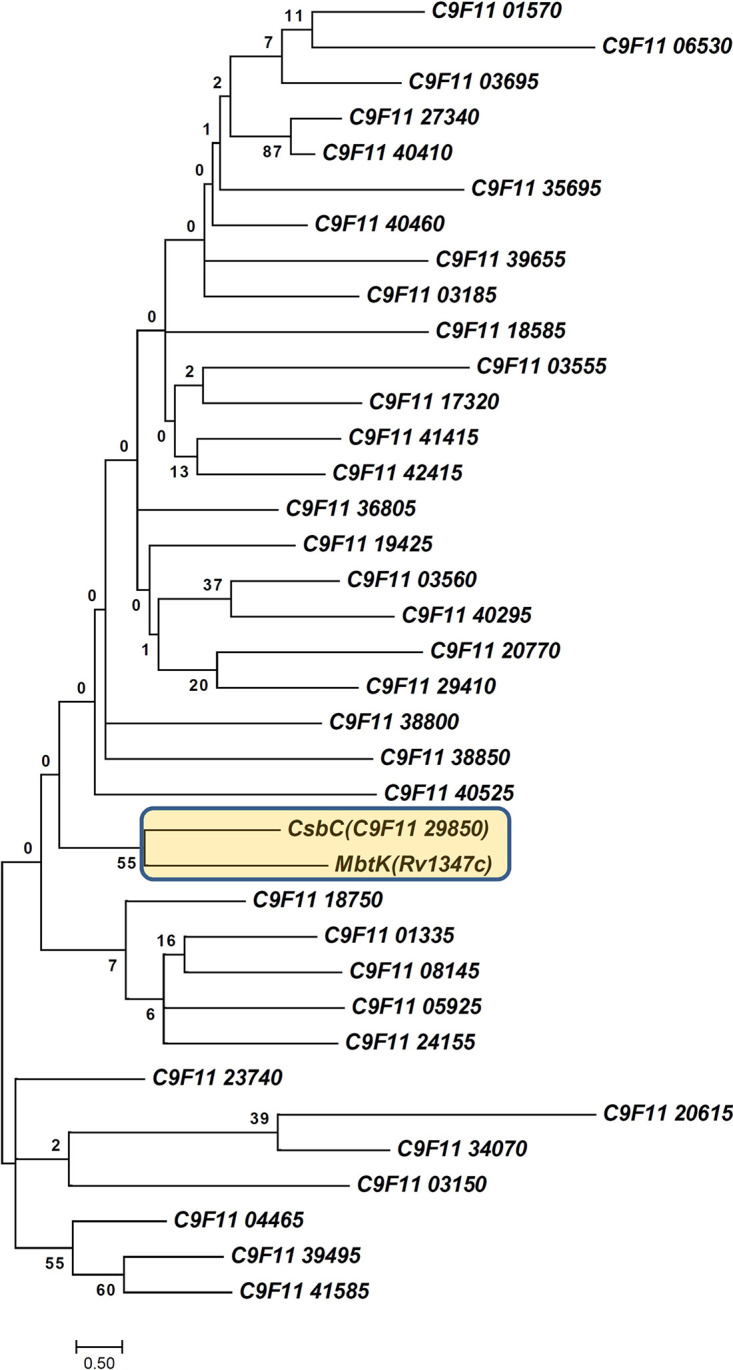
Phylogenetic tree constructed by MEGAX software (31) for the GNAT-like proteins encoded by the *Streptomyces* sp. strain YIM 121038 genome using the maximum likelihood method. The analysis involved 37 amino acid sequences, including a proposed *N*-succinyltransferase of M. tuberculosis.

Since the *N*-succinylated congener desertomycin A was found to be antibiotically inactive, we focused on identification of an enzyme that might be responsible for the transformation of desertomycin A into desertomycin X. The desertomycin BGC (16) could be readily identified in the genome of YIM 121038. Since no genes coding for *N*-acyltransferases that could catalyze the aforementioned reaction were identified within the desertomycin cluster (BGC28) (Table S1), the YIM 121038 genome was manually mined for genes encoding GNAT-like proteins. A total of 36 genes encoding putative GNAT-like enzymes were identified, and their products, along with the MbtK GNAT-like protein from Mycobacterium tuberculosis known to bind succinyl-CoA (17) were used to construct a phylogenetic tree (Fig. 2). One of the GNAT-like proteins, C9F11 29850, was found to be most closely related to MbtK and hence became the focus of further investigation.

**FIG 3:**
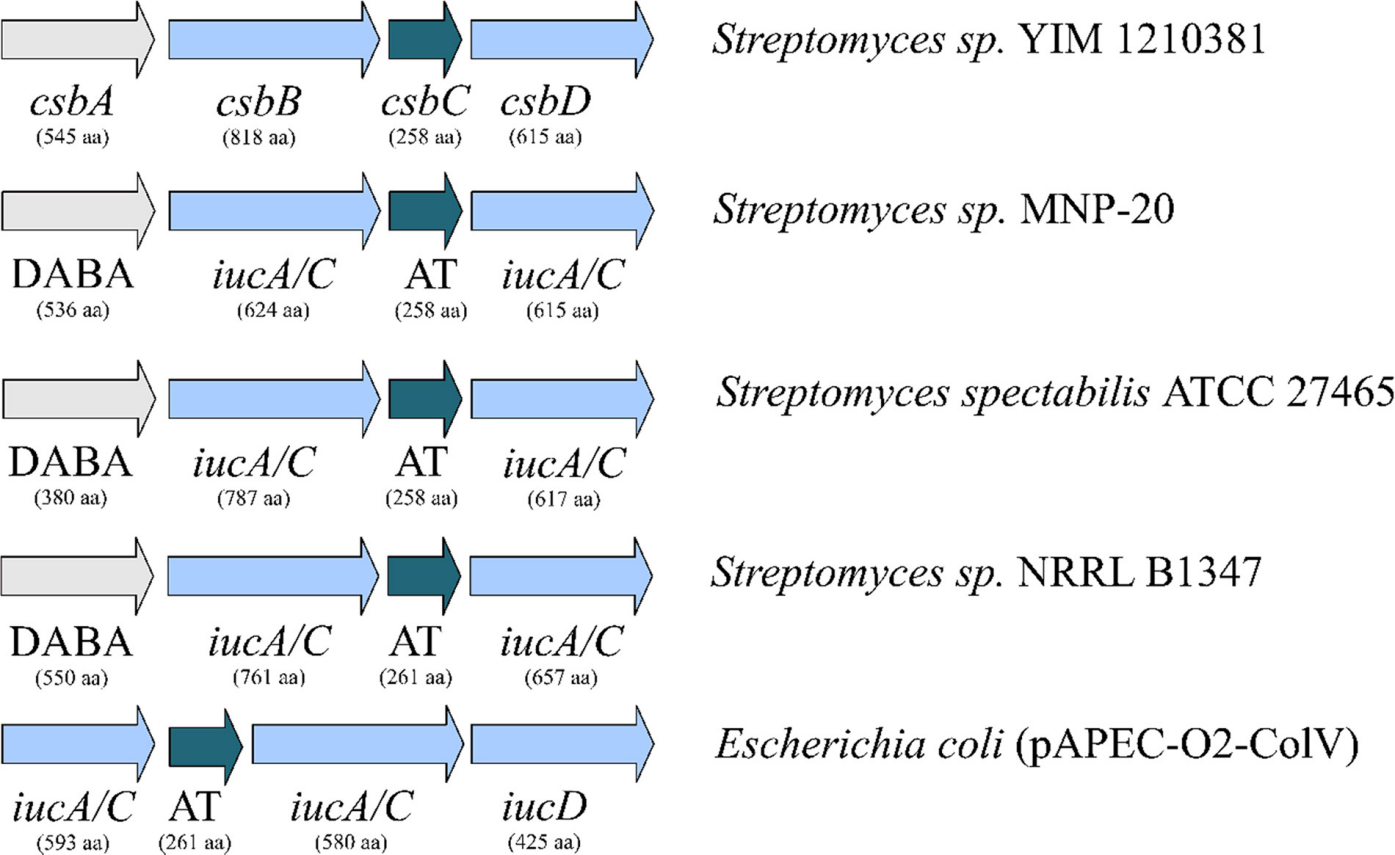
Genetic organization of the cryptic siderophore biosynthesis gene cluster (BGC) *csb* in *Streptomyces* sp. strain YIM 121038, its closest homologues in other *Streptomyces* spp., and the Escherichia coli aerobactin BGC. DABA, diaminobutyrate-2-oxoglutarate transaminase; IucA/C, siderophore synthetase component; AT, *N*-acyltransferase; IucD, NADPH-dependent l-lysine *N*(6)-monooxygenase.

The gene encoding C9F11 29850 was found within BGC20 (Table 1 and Fig. 3), which was predicted to specify the biosynthesis of a putative siderophore. The composition of genes in BGC20, designated *csb*, is similar to that of the *iucABCD* cluster in E. coli for the biosynthesis of the siderophore aerobactin (18), except for the *iucD*-like gene encoding l-lysine 6-monooxygenase, which is absent in the *csb* cluster. Instead, the *csbA* gene, presumably encoding diaminobutyrate-2-oxoglutarate transaminase, was identified, of which homologues are found in several siderophore BGCs in bacteria (19). Applying both a GNPS library search (https://gnps.ucsd.edu) and targeted search for fragment ions typical for hydroxamate siderophores or the succinyl moiety on the liquid, chromatography-mass spectrometry (LC-MS) data of the liquid culture failed to uncover any potential product of *csb* or another siderophore-like compound.

**TABLE 1:** *Streptomyces* sp. strain YIM 121038 *csb* biosynthetic gene cluster

Gene	Product length (aa)	Protein homolog	Accession no.	Identity (%)	Proposed function
*orf1*	443	Hypothetical protein, *Streptomyces* sp. strain MNP-20	WP_172386112.1	99	Unknown
*csbA*	545	Dat2, *Streptomyces* sp. strain MNP-20	WP_172386144.1	98	Diaminobutyrate-2-oxoglutarate transaminase
*csbB*	818	IucA, Streptomyces spectabilis	WP_170316513.1	83	Siderophore synthetase component
*csbC*	258	IucB, *Streptomyces* sp. strain MNP-20	WP_216678075.1	96	*N*-Acyltransferase
*csbD*	615	IucC, *Streptomyces* sp. strain MNP-20	WP_216678074.1	98	Siderophore synthetase component
*orf2*	663	DinG, *Streptomyces* sp. strain MNP-20	WP_172386109.1	99	ATP-dependent DNA helicase
*orf3*	261	LexA, multispecies of *Streptomyces*	WP_138962193.1	100	Transcriptional regulator
*orf4*	184	NrdR, *Streptomyces* sp. strain NRRL B-1347	WP_030680473.1	99	Transcriptional regulator

The CsbC protein encoded by the *csb* cluster is a homolog of the IucB *N*-acetyltransferase and was predicted to catalyze the acetylation of *N′*-hydroxylysine. Several attempts to inactivate the *csbC* gene in YIM121038 via gene disruption failed (data not shown), indicating that the product of *csbC*, or the *csb* cluster-specified siderophore, may be essential for this strain under the conditions used. To test our hypothesis that CsbC can succinylate desertomycin A, a codon-optimized *csbC* gene with an *N*-terminal His_6_ tag was expressed in E. coli BL21(DE3) and purified, and its *N*-succinyltransferase activity was tested with desertomycin A. In the *in vitro* enzyme assay, formation of desertomycin X was confirmed using LC-tandem MS (MS/MS) analysis of the reaction mixture (Fig. S3A).

10.1128/mbio.01789-22.3 FIG S3 (A) CsbC *in vitro* activity on desertomycin A (Des A). Total ion chromatograms (*m*/*z* 140 to 2,000) of the reaction mixture containing CsbC, desertomycin A, and either succinyl (S) coenzyme A (a and b) or acetyl (Ac) coenzyme A (c) obtained by LC-MS in positive-ion mode. The reaction was quenched either before (a) or after (b and c) incubation for 2 h at 30°C—the former as a negative control. Desertomycin X (Des X) is the *N*-succinylated derivative of Des A. (b) CsbC *in vitro* activity on daptomycin. Shown are total ion chromatograms (*m*/*z* 140 to 2,000) of the reaction mixture containing CsbC, succinyl coenzyme A, and daptomycin (Dap) obtained by LC-MS in positive-ion mode. The reaction was quenched either before (a) or after (b) incubation for 2 h at 30°C—the former as a negative control. (C) CsbC *in vitro* activity on ethionamide (Eth). Shown are the extracted ion chromatograms for *m*/*z* 167.0637 ± 0.0017 (black line) and *m*/*z* 267.0798 ± 0.0027 (red line) of the reaction mixture containing CsbC, succinyl coenzyme A, and ethionamide (Eth) obtained by LC-MS in positive-ion mode. The reaction was quenched either before (a) or after (b) incubation for 2 h at 30°C—the former as negative control. (D) CsbC *in vitro* activity on trimethoprim (Tri). Shown are extracted ion chromatograms for *m*/*z* 291.1452 ± 0.0029 (black line) and *m*/*z* 391.1612 ± 0.0039 (red line) of the reaction mixture containing CsbC, succinyl coenzyme A, and trimethoprim obtained by LC-MS in positive-ion mode. The reaction was quenched either before (a) or after (b) incubation for 2 h at 30°C—the former as a negative control. (E) CsbC *in vitro* activity on vancomycin (Van). Shown are extracted ion chromatograms for *m*/*z* 724.7224 ± 0.0072 (black line), *m*/*z* 774.7304 ± 0.0077 (red line), and *m*/*z* 824.7384 ± 0.0082 (green line) of the reaction mixture containing CsbC, succinyl coenzyme A, and vancomycin obtained by LC-MS in positive-ion mode. The reaction was quenched either before (a) or after (b) incubation for 2 h at 30°C—the former as a negative control. (F) CsbC *in vitro* activity on colistin (Col). Shown are extracted ion chromatograms for *m*/*z* 578.3822 ± 0.0058 (black line), *m*/*z* 628.3903 ± 0.0063 (red line), *m*/*z* 678.3983 ± 0.0068 (green line), *m*/*z* 728.4063 ± 0.0073 (blue line), *m*/*z* 778.4143 ± 0.0078 (beige line), and *m*/*z* 828.4224 ± 0.0083 (purple line) of the reaction mixture containing CsbC, succinyl coenzyme A, and colistin obtained by LC-MS in positive-ion mode. The reaction was quenched either before (a) or after (b) incubation for 2 h at 30°C—the former as a negative control. (G) CsbC *in vitro* activity on tetracycline (Tet). Shown are extracted ion chromatograms for *m*/*z* 445.1605 ± 0.0045 (black line), and *m*/*z* 545.1766 ± 0.0055 (red line) of the reaction mixture containing CsbC, succinyl coenzyme A, and tetracycline obtained by LC-MS in positive-ion mode. The reaction was quenched either before (a) or after (b) incubation for 2 h at 30°C—the former as negative control. (H) CsbC *in vitro* activity on gentamicin (Gen). HR-ESI mass spectra (*m*/*z* 200 to 700, retention time [RT] 0.40 to 3.44 min) of the reaction mixture containing CsbC, succinyl coenzyme A, and gentamicin obtained by LC-MS in positive-ion mode. The reaction was quenched either before (a) or after (b) incubation for 2 h at 30°C—the former as a negative control. (I) CsbC *in vitro* acetylation activity on kanamycin (Kan). High-resolution ESI mass spectra (*m*/*z* 450 to 600, RT 0.40 to 4.00 min) of the reaction mixture containing CsbC, acetyl (Ac) coenzyme A, and kanamycin obtained by LC-MS in positive-ion mode. The reaction was quenched either before (a) or after (b) incubation for 2 h at 30°C—the former as a negative control. (J) CsbC *in vitro* activity on ornithine (Orn). Shown are extracted ion chromatograms for *m*/*z* 133.0972 ± 0.0050 (black line), *m*/*z* 233.1132 ± 0.0050 (red line), and *m*/*z* 333.1292 ± 0.0050 (green line) of the reaction mixture containing CscB-S, succinyl coenzyme A, and ornithine obtained by LC-MS in positive-ion mode. The reaction was quenched either before (a) or after (b) incubation for 2 h at 30°C—the former as a negative control. (K) CsbC *in vitro* activity on putrescine (Put). Shown are total ion chromatograms (*m*/*z* 60 to 1,500) of the reaction mixture containing CscB-S, succinyl coenzyme A, and putrescine obtained by LC-MS in positive-ion mode. The reaction was quenched either before (a) or after (b) incubation for 2 h at 30°C—the former as a negative control. Download FIG S3, PDF file, 0.6 MB. Copyright © 2022 Schneider et al. 2022 Schneider et al. https://creativecommons.org/licenses/by/4.0/ This content is distributed under the terms of the Creative Commons Attribution 4.0 International license.

### CsbC-catalyzed inactivation of antibiotics and its substrate specificity *in vitro*.

**FIG 4:**
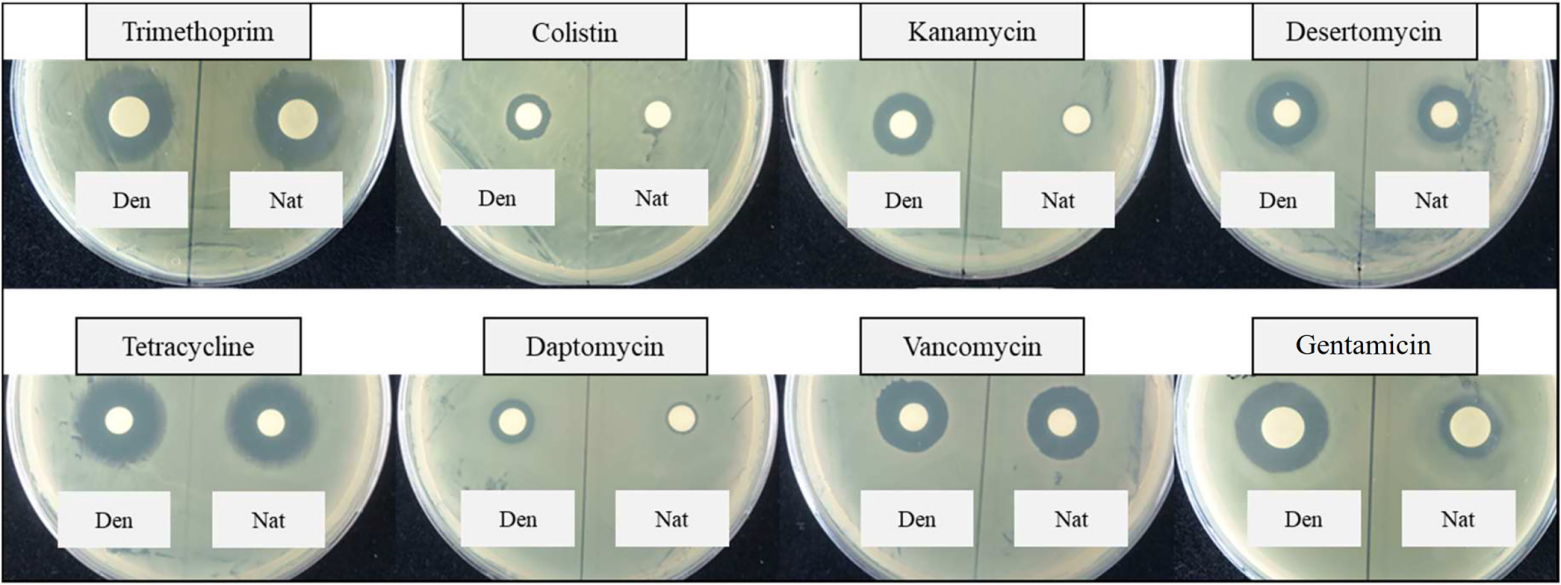
Diffusion disc bioactivity test of various antibiotics after incubation with CsbC and succinyl-CoA. The trimethoprim and colistin samples were tested against E. coli, while the samples with kanamycin, desertomycin, tetracycline, daptomycin, vancomycin, and gentamicin were tested against B. subtilis. “Den” (CsbC denatured) indicates samples with 0.5% formic acid added to inactivate the enzyme before addition of antibiotics, which were used as negative controls. “Nat” (CsbC native) denotes samples with native enzyme where the same amount of formic acid was added after the reaction was finished.

**FIG 5:**
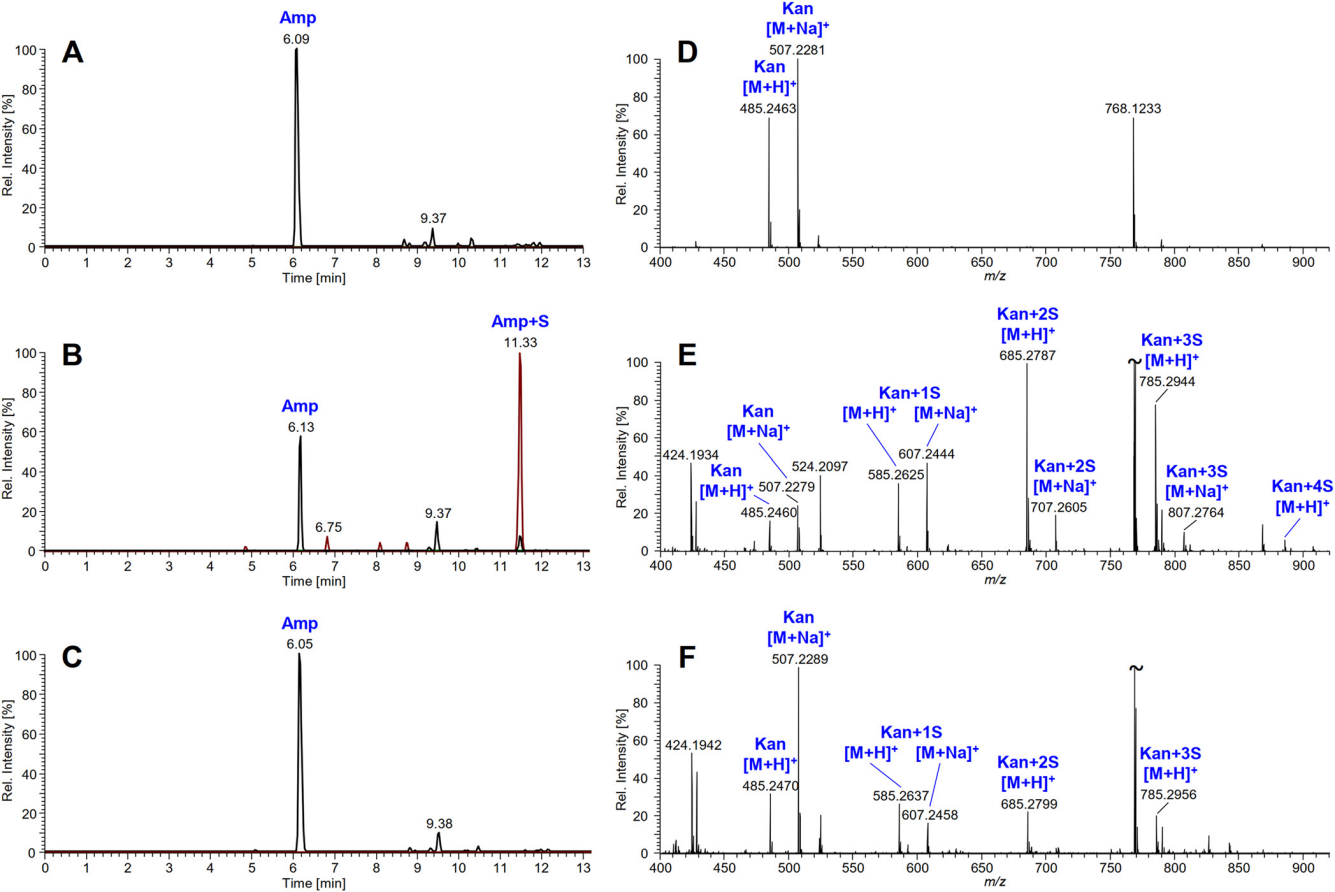
CsbC *in vitro* activity on ampicillin (A–C) and kanamycin (D–F). Extracted ion chromatograms for *m/z* 350.1169 ± 0.0035 (black line, corresponding to Amp) and *m/z* 450.1329 ± 0.0045 (red line, corresponding to succinylated Amp) of the reaction mixture containing CsbC, ampicillin (Amp), and succinyl (S) coenzyme A (A and B) or acetyl (Ac) coenzyme A (C) obtained by LC-MS in positive ion mode. The reaction was quenched either before (A) or after (B and C) incubation for 2 h at 30 °C – the former as negative control. High resolution ESI mass spectra (*m/z* 400-920, average over Rt 0.40-4.00 min) of the reaction mixture containing CsbC, succinyl (S) coenzyme A, and kanamycin (Kan) obtained by LC-MS in positive ion mode. The reaction was quenched either before (A) or after (B and C) incubation for 2 h at 30 °C – the former as negative control. Inhibition of CsbC by pefloxacin was tested by addition of 1 mM pefloxacin mesylate dehydrate to the reaction mixture (C).

**FIG 6:**
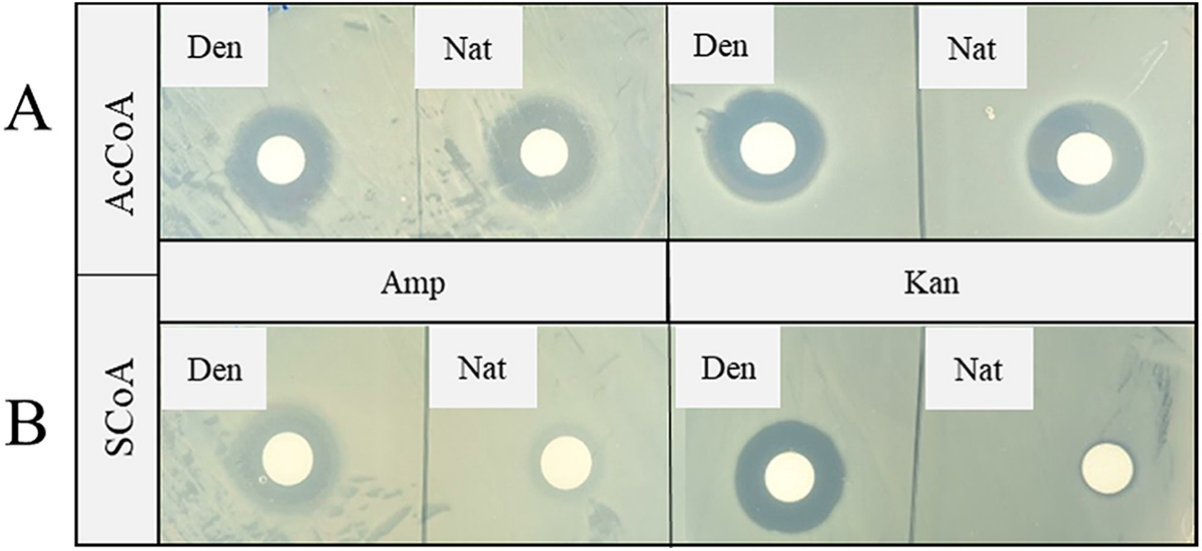
Comparison of CsbC selectivity for the substrates acetyl-CoA (AcCoA) (A) and succinyl-CoA (SCoA) (B) tested in acylation reactions with ampicillin (Amp) and kanamycin (Kan). The diffusion disc assay test was applied with E. coli for Amp and B. subtilis for Kan. Samples with 0.5% formic acid (to inactivate the enzyme before addition of antibiotics, where “Den” represents CsbC-S denatured) were used as negative controls. The same amount of formic acid was added to the samples with native CsbC-S enzyme (Nat) after the reaction was finished.

Based on the fact that desertomycin X did not show any antibacterial activity in the bioassays, we further hypothesized that *N*-succinylation by CsbC might represent a resistance mechanism. We next tested eight structurally diverse antibiotics in the *in vitro* succinylation reactions involving recombinant CsbC, followed by bioassays and LC-MS/MS analyses. Colistin, kanamycin, daptomycin, gentamicin (Fig. 4), and ampicillin (see Fig. 6B) lost their activity after treatment with CsbC, while no significant changes in antibiotic activity were observed for trimethoprim, tetracycline, and vancomycin in these assays (Fig. 4). Some decrease in bioactivity was detected for the reaction mixture with desertomycin A as a substrate. The antimicrobial activity of ethionamide was not tested in the bioassay, and succinylation of this compound was analyzed with LC-MS only (Table 2). Extracted ion chromatograms for ampicillin and mass spectra for kanamycin treated with CbsC and showing efficient conversion of the two antibiotics to succinylated derivatives are shown in Fig. 5.

**TABLE 2:** *N*-Succinylation of the tested antibiotics by recombinant CsbC protein

Tested substrate	No. of:		
	Primary/secondary aliphatic amine groups	Aromatic amine groups	Succinyl groups added by CsbC
Ampicillin	1/0	0	1
Colistin	5/0	0	1–5
Daptomycin	1/0	1	1
Desertomycin A	1/0	0	1
Ethionamide	0/0	0	0
Gentamicin	3/2^*a*^	0	1–3 (4 in traces)
Kanamycin	4/0^*b*^	0	1–4
Tetracycline	0/0^*c*^	0	1
Trimethoprim	0/0	2	1 (traces)
Vancomycin	1/1	0	1 (2 in traces)

a For gentamicin C_1_.

b For kanamycins A and C.

c One tertiary aliphatic amine that cannot be succinylated.

The selectivity of CsbC toward the acyl group donor was tested by substituting for succinyl-CoA with acetyl-CoA in the *in vitro* reactions. The antibiotic activities of the ampicillin and kanamycin that were used in this experiment were not affected when acetyl-CoA was used (Fig. 6A), in contrast to what was observed with succinyl-CoA as cosubstrate (Fig. 6B). Hence, it was concluded that the latter acyl-CoA is the preferred CsbC acyl donor under the conditions tested.

In line with the observed decrease in antibiotic activity, colistin, kanamycin, and ampicillin were found by the LC-MS analysis to be the most favored substrates of CsbC. In all three cases, the signal intensities of the succinylated derivatives were higher than the ones of the remaining nonmodified antibiotics. Desertomycin A, gentamicin, daptomycin, tetracycline, and vancomycin were succinylated to a lesser extent, while ethionamide and trimethoprim were barely or not at all accepted as the substrates (Fig. S3A to H). These results strongly suggest that sterically unhindered aliphatic amine groups are preferred sites of modification by CsbC, while aromatic amine groups are not or only very weak acceptors (Table 2). This activity is not restricted to antibiotics, as was confirmed by the formation of *N*- and *N*,*N′*-succinylated derivatives of the antibiotically inactive primary metabolites putrescine and ornithine (Fig. S3J and K).

According to the MS/MS spectra, the preferred succinylation site in vancomycin is the secondary amine on the glycopeptide’s N terminus (data not shown), presumably because the primary amine is localized on a sterically hindered tertiary carbon. In the case of tetracycline, where we would expect no amide formation to occur, the small amount of detected succinylation is apparently due to the formation of an ester with an nonphenolic OH group, as demonstrated by an [M-C_4_H_6_O_4_+H]^+^ fragment ion in the MS/MS spectrum (data not shown). Presumably, this reaction is also catalyzed by CsbC, since no such product is observed in the reaction mixtures quenched with formic acid (FA) before incubation.

According to the LC-MS analyses, acetyl-CoA can be used by CsbC as acyl-group donor instead of succinyl-CoA for some of the tested antibiotics, but generally with a much lower efficiency. Acetylated products were detected for kanamycin (Fig. S3I), desertomycin A (Fig. S3A), and, to a very low extent, for vancomycin, but not for ampicillin and daptomycin (data not shown).

To test whether the formation of desertomycin X by CsbC is related to self-resistance, its production in relation to desertomycin A was followed over 6 days of cultivation, whereby the culture medium and the cell pellet were separated before extraction. *Streptomyces* sp. strain YIM 121038 was found to be a very prolific producer of desertomycin A, with high levels being detected already after 1 day of cultivation in both the pellet and the supernatant (Fig. S4A to F). Relevant amounts of desertomycin X were only detected at day 2, but at low levels compared to desertomycin A. Furthermore, *N*-acetyl desertomycin A was produced at even higher levels than desertomycin X. The concentrations of all three congeners did not increase much or even decrease again after 2 days of fermentation under the given conditions. Interestingly, the relative abundance of desertomycin X compared to desertomycin A and *N*-acetyl desertomycin A was much higher in the supernatant. None of these findings are in line with *N*-succinylation or *N*-acetylation of desertomycin A being relevant for self-resistance, but fit the low affinity of CsbC for desertomycin A found *in vivo*. The LC-MS data from the whole extracts also revealed the isomerization of all three congeners over time, particularly in the supernatant.

10.1128/mbio.01789-22.4 FIG S4 (A) Monitoring the production of desertomycin congeners in *Streptomyces* sp. strain YIM 121038 over 6 days of cultivation in TSB medium. Base peak chromatograms (*m*/*z* 100 to 2,000) of the cell pellet extract after 1 (a), 2 (b), 3 (c), 4 (d), and 6 (e) days of fermentation. Desertomycin A (Des A), desertomycin X (Des X), *N*-acetyl desertomycin A (Ac-Des A), and uncharacterized isomers of them (Des A′, Des X′, Ac-Des A′) were identified. (B) Monitoring the production of desertomycin congeners in *Streptomyces* sp. strain YIM 121038 over 6 days of cultivation in TSB medium. Shown are extracted ion chromatograms of the cell pellet extract for the [M + H]^+^ ions of desertomycin A (*m*/*z* 1,192.7565 ± 0.0100 [green line]), desertomycin X (*m*/*z* 1,292.7725 ± 0.0100 [dark red line]), and *N*-acetyl desertomycin A (*m*/*z* 1,234.7671 ± 0.0100 [blue line]) after 1 (a), 2 (b), 3 (c), 4 (d), and 6 (e) days of fermentation. (C) Monitoring the production of desertomycin congeners in *Streptomyces* sp. strain YIM 121038 over 6 days of cultivation in TSB medium. Base peak chromatograms (*m*/*z* 100 to 2,000) of the supernatant extract after 1 (a), 2 (b), 3 (c), 4 (d), and 6 (e) days of fermentation. Desertomycin A, desertomycin X, *N*-acetyl desertomycin A, and uncharacterized isomers of them were identified. (D) Monitoring the production of desertomycin congeners in *Streptomyces* sp. strain YIM 121038 over 6 days of cultivation in TSB medium. Shown are extracted ion chromatograms of the supernatant extract for the [M + H]^+^ ions of desertomycin A (*m*/*z* 1,192.7565 ± 0.0100 [green line]), desertomycin X (*m*/*z* 1,292.7725 ± 0.0100 [dark red line]), and *N*-acetyl desertomycin A (*m*/*z* 1,234.7671 ± 0.0100 [blue line]) after 1 (a), 2 (b), 3 (c), 4 (d), and 6 (e) days of fermentation. (E) Monitoring the production of desertomycin congeners in *Streptomyces* sp. strain YIM 121038 over 6 days of cultivation in TSB medium. Peak areas were obtained from the extracted ion chromatograms of the cell pellet extract for the [M + H]^+^ ions of desertomycin A, desertomycin X, *N*-acetyl desertomycin A, and uncharacterized isomers of them. The weight of freeze-dried supernatant was determined before extraction. (F) Monitoring the production of desertomycin congeners in *Streptomyces* sp. strain YIM 121038 over 6 days of cultivation in TSB medium. Peak areas were obtained from the extracted ion chromatograms of the supernatant extract for the [M + H]^+^ ions of desertomycin A, desertomycin X, *N*-acetyl desertomycin A, and the uncharacterized isomers of them. The weight of the freeze-dried pellet was determined before extraction. Download FIG S4, PDF file, 0.7 MB. Copyright © 2022 Schneider et al. 2022 Schneider et al. https://creativecommons.org/licenses/by/4.0/ This content is distributed under the terms of the Creative Commons Attribution 4.0 International license.

### CsbC provides for low-level kanamycin and ampicillin resistance *in vivo*.

The results of the *in vitro* studies supported our hypothesis that CsbC can catalyze the attachment of a succinyl moiety to various antibiotics with amino groups and that, in some cases, this modification can lead to the entire loss of antibiotic activity. Considering this, we tested the kanamycin resistance of an S. lividans TK24/pUWL-*csbC* strain, which expresses the native *csbC* gene. Growth of S. lividans TK24/pUWL-*csbC* in liquid medium and on the agar plates with up to 7 μg/mL of kanamycin (concentration causing complete growth inhibition) was monitored. No differences in growth could be detected compared to the control strain S. lividans TK24/pUWLoriT (empty vector) and the strain expressing *csbC*.

**FIG 7:**
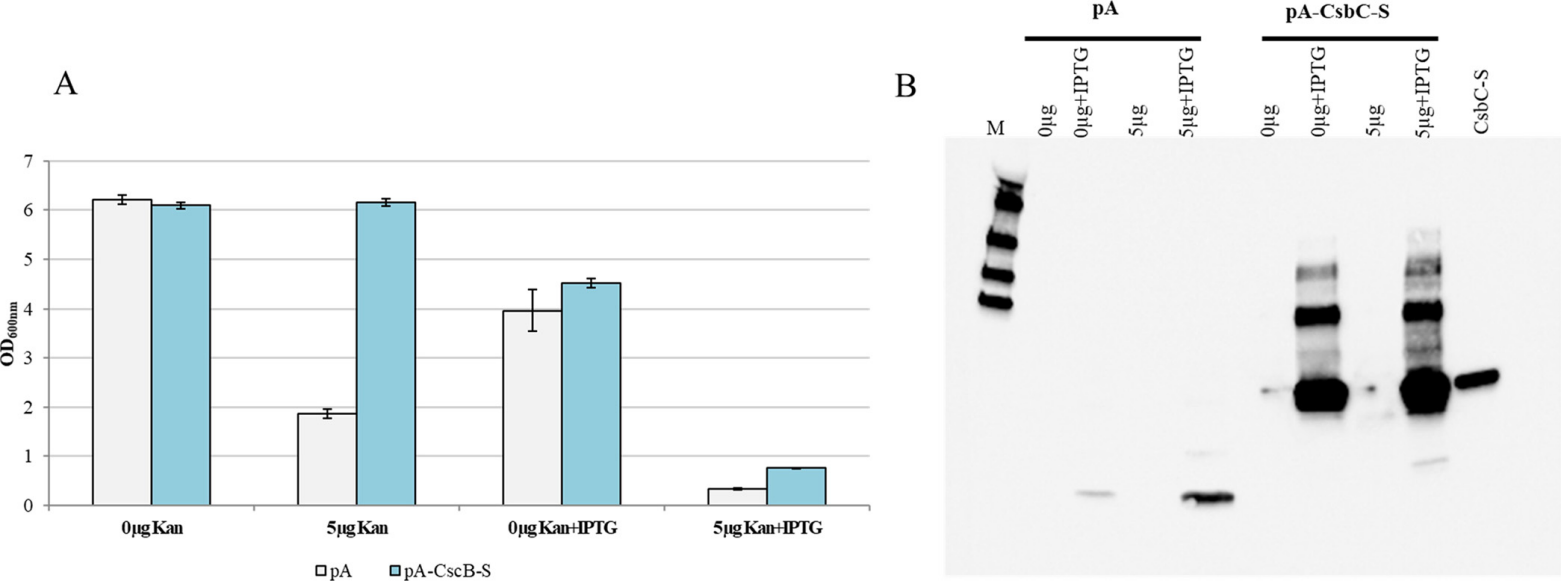
Development of kanamycin resistance in E. coli BL21 after expression of CsbC *in vivo*. (A) Bacterial growth after 24-h cultivation in LB medium with 0 and 5 μg/mL kanamycin (Kan) and with/without IPTG induction; (B) Western blot analysis of crude extracts from panel A and comparison to 2.6 μg of purified CsbC protein. pA, empty vector; pA_CsbC, IPTG-inducible CsbC-expressing vector.

Next, kanamycin resistance of E. coli BL21/pA_CsbC, which harbors a vector with an ampicillin resistance gene and provides for IPTG (isopropyl-β-d-thiogalactopyranoside)-inducible CsbC expression, was tested. It was expected that after addition of IPTG, the production of recombinant CsbC will be induced and the intracellular kanamycin will be inactivated via *N*-succinylation. After 24 h of cultivation in the kanamycin-supplemented medium with and without IPTG induction, the BL21/pA_CsbC strain grew considerably better than the control strain BL21/pA (Fig. 7A). At the same time, it was obvious that induction with IPTG significantly suppresses the bacterial growth of both the CsbC-expressing strain and the control strain. Western blot analysis of samples demonstrated a weak expression of CsbC in BL21/pA_CsbC cultures, even without IPTG addition, indicating T7 promoter leakage (Fig. 7B). Apparently, this weak expression was nevertheless sufficient to enhance resistance of strain BL21/pA_CsbC to kanamycin.

**FIG 8:**
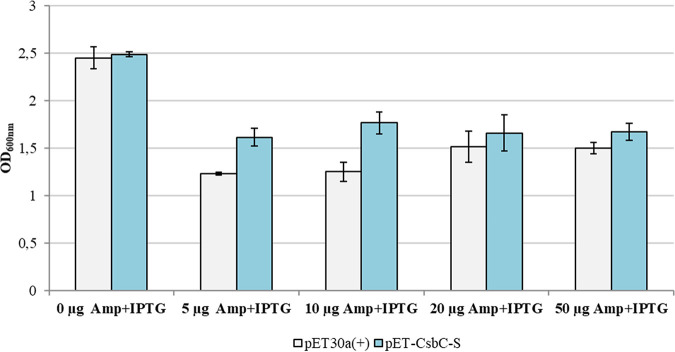
Development of ampicillin resistance in E. coli BL21 after expression of CsbC *in vivo* with the pET30a(+) expression system. Bacterial growth after 24-h cultivation in LB medium with 0 to 50 μg/mL ampicillin (Amp) and with 0.1 mM IPTG induction was monitored via OD_600_ measurements.

The development of a weak resistance in BL21/pET-CsbC cells was also detected in the experiment with ampicillin. Here, we could show that with all tested ampicillin concentrations, the strain carrying the CsbC-expressing vector grew better than the control E. coli BL21 strain with the pET30a(+) vector (Fig. 8). The difference in growth between the two strains could be best seen in medium containing the smallest amounts of Amp (5 and 10 μg/mL). Due to the fact that CsbC showed a broad substrate specificity, we decided to study its kinetics in reaction with ampicillin as the substrate. The initial idea of using kanamycin as a substrate was rejected due to the presence of several amino groups that were apparently modified by this enzyme. Ampicillin, on the contrary, harbors only one amino group, which makes the analysis more precise than that with kanamycin. *V*_max_ and *K_m_* constants were determined based on the formation of *N*-succinylated ampicillin (Amp-S), the concentration of which was calculated from the data from HPLC analysis (Fig. S5). The Microsoft Excel Solver was applied in this calculation to increase the accuracy of data interpretation, which resulted in calculated values for of 0.47 mM for *K_m_* and 0.61 mM/min for *V*_max_.

10.1128/mbio.01789-22.5 FIG S5 Enzyme kinetic analysis of CsbC-S. Plotted are raw data of activity measurements against different ampicillin concentrations and a Solver plot with determined *V*_max_ and *K_m_* values according to the Solver analysis tool. Download FIG S5, PDF file, 0.3 MB. Copyright © 2022 Schneider et al. 2022 Schneider et al. https://creativecommons.org/licenses/by/4.0/ This content is distributed under the terms of the Creative Commons Attribution 4.0 International license.

## DISCUSSION

It is now widely acknowledged that antibiotic resistance genes have most likely originated from antibiotic-producing bacteria, which had to protect themselves from the action of endogenously produced antibiotics (20). In this work, we identified in a *Streptomyces* sp. a new congener of the known antibiotic desertomycin A, designated desertomycin X, which is antibiotically inactive and only differs from the former by the presence of an *N-*succinyl group. This suggested that *N*-succinylation may represent a previously undescribed resistance mechanism. Although resistance genes are in most cases colocalized with the antibiotic biosynthesis genes, no such gene, which would presumably encode an *N-*succinyltransferase, was found in the desertomycin biosynthesis gene cluster. The search for a possible candidate led us to examine a BGC termed *csb* that specifies the biosynthesis of a cryptic siderophore. A putative GNAT family acyltransferase encoded by the *csbC* gene in this cluster distinguished itself from known *N-*acyltransferases that use acetyl-CoA as acyl group donor to modify and inactivate certain antibiotics, such as aminoglycosides (7). In particular, CsbC had an ca. 80-amino-acid-extended N terminus which contained a polyproline signature, a feature found only in homologous uncharacterized proteins encoded by similar BGCs in 3 *Streptomyces* spp. CsbC contains domains with similarity to the AAC(6′) protein Rv1347c, which was suggested by Card et al. to be involved in both antibiotic resistance and mycobactin biosynthesis in Mycobacterium tuberculosis (17). However, neither role of the Rv1347c protein was experimentally verified in enzyme assays with acetyl-CoA, propionyl-CoA, and butyryl-CoA. We hypothesized that CsbC may be responsible for the *N-*succinylation of desertomycin A, which was confirmed via expression of recombinant CsbC in E. coli followed by *N-*acylation activity testing *in vitro*. Moreover, CsbC could efficiently *N-*succinylate structurally distinct antibiotics as well as some primary metabolites. Some of those, in which an amino group important for interaction with the molecular target was blocked via succinylation, lost all or most of their antibiotic activity. Acetyl-CoA, however, could not be used by CsbC as an acyl group donor as efficiently as succinyl-CoA in our experiments.

Next, we tested the ability of CsbC to confer antibiotic resistance *in vivo*. Expression of the native, non-codon-optimized *csbC* in S. lividans did not lead to increased kanamycin resistance in this bacterium. This may be explained either by the lower pool of freely available succinyl-CoA in this bacterium under the conditions tested or by the low expression of the elongation factor P known to be essential for the synthesis of proteins with polyproline motifs (21). However, the fact that antibiotically inactive *N*-succinylated congener of desertomycin A was isolated from *Streptomyces* sp. strain YIM 121038 suggests that CsbC is active *in vivo* and may contribute to the antibiotic resistance in natural environment. Notably, the recombinant CsbC was able to confer kanamycin and ampicillin resistance in E. coli, albeit only to a low level. In the experiment with kanamycin, it was apparent that IPTG-induced CsbC expression detrimentally affected the growth of recombinant E. coli BL21, most likely due to the overexpression of T7 polymerase (22), but maybe also due to the depletion of succinyl-CoA, which is a less abundant cellular metabolite than acetyl-CoA (23). Another explanation could be succinylation of primary metabolites, such as putrescine and ornithine (see the Results section), that would render them unsuitable for anabolic pathways. However, we did not observe such an effect in the experiment with ampicillin, which speaks in favor of the former hypothesis of succinyl-CoA depletion. (Kanamycin can accept four succinyl moieties, while ampicillin accepts only one.)

Concentrations of antibiotics in natural, nonpolluted environmental samples, such as soil and water, are presumed to be very low, and the physiology of bacteria dwelling in these environments must differ significantly from that when bacteria are cultivated in the laboratory (24). Considering this, it seems plausible that *csbC* homologues, while encoding an essential part of the siderophore biosynthesis pathway, may at the same time confer antibiotic resistance in the natural environment. Targeting CsbC-like proteins for inhibition may thus have a dual effect on the viability of bacteria due to both the depletion of essential ions and prevention of the antibiotic resistance phenotype.

## MATERIALS AND METHODS

### Isolation and identification of *Streptomyces* sp. strain YIM 121038.

The soil sample was collected in May 2012 in the tropical rain forest in Xishuangbanna, Yunnan Province, People’s Republic of China, at an altitude of 488 m and latitude and longitude N21°30′, E100°46′. *Streptomyces* sp. strain YIM 121038 was isolated by using YIM 171 medium (raffinose, 5.0 g/L; histidine, 1.0 g/L; K_2_HPO_4_, 1.0 g/L; MgSO_4_·7H_2_O, 0.5 g/L; agar, 15.0 g/L; water to 1 L [pH 7.2 to 7.4]) and incubation temperature of 28°C. Genomic DNA of YIM 121038 was isolated as described below and was used as a template to PCR amplify a 16S rRNA gene fragment using a previously described protocol (25). The resulting DNA fragment was sequenced, and the sequence was deposited in GenBank.

### Genome sequencing and analyses.

For genome sequencing, chromosomal DNA was used to generate a TruSeq PCR-free library that was sequenced on an Illumina MiSeq system in a 2× 300-nucleotide (nt) run. A total of 559.3 Mbp of sequence materials (52.4× coverage) was assembled using Newbler version 2.8 (Roche), resulting in 117 scaffolds containing 214 contigs. Subsequently, 400 ng of genomic DNA was used to generate a second shotgun library for sequencing on the MinION system (Oxford Nanopore Technologies). Using the rapid barcoding kit (SQK-RBK004), a one-dimensional (1D) sequencing library was created and sequenced on an R9.5 flow cell in a 24-h run on the MinION sequencer. Base calling and sequence material conversion were performed using Albacore version 2.1.7 (Oxford Nanopore Technologies), yielding 11,928 reads with a total length of 166.6 Mbp (13,964-nt average read length, 15× coverage). The assembly was performed with Canu version 1.6 (26). After assembly, the resulting three contigs were polished with the short Illumina reads using Pilon (27). The final assembly and polishing were done manually using Consed (28) to assemble the two contigs making up the chromosome and to resolve polishing artifacts in repetitive regions. This resulted in two contigs representing the complete linear chromosome and the complete linear plasmid pSSP121038. Gene prediction and annotation were performed applying Prokka (29).

Phylogenetic analyses of 16S rRNA gene sequence and protein sequences of CsbB and its homologues were performed with MEGA7 (30).

### Generation of recombinant bacterial strains, plasmids, and general growth conditions.

The genomic DNA from *Streptomyces* was isolated using the Wizard Genomic DNA purification kit (Promega, USA), as described previously (31). The culture was prepared by inoculation of 50 mL of 3% tryptic soy broth (TSB) medium (Oxoid, United Kingdom) with 50 μL of fresh prepared spore suspension (20% [vol/vol] glycerol) and incubation in 250-mL baffled flasks at 28°C at 250 rpm. All routine DNA standard techniques, cloning methods, and plasmid transformation into Escherichia coli were performed as described by Sambrook et al. (32). PCR amplifications were done with Q5 high-fidelity DNA polymerase (New England Biolabs) using oligonucleotides listed in Table S3A in the supplemental material. Plasmids and bacterial strains used or constructed during this study are represented in Table S3B.

10.1128/mbio.01789-22.8 TABLE S3 (A) Oligonucleotides used in this study. Italic indicates an endonuclease restriction enzyme site. (B) Plasmids and bacterial strains used in this work. Download Table S3, DOCX file, 0.02 MB. Copyright © 2022 Schneider et al. 2022 Schneider et al. https://creativecommons.org/licenses/by/4.0/ This content is distributed under the terms of the Creative Commons Attribution 4.0 International license.

Escherichia coli strains were grown in Luria-Bertani (LB) broth or on LB agar supplemented with chloramphenicol (25 μg mL^−1^), apramycin (100 μg mL^−1^), and kanamycin (25 μg mL^−1^). XL1-Blue was used for general cloning, and ET12567(pUZ8002) was used for intergeneric conjugative transfer of plasmids to *Streptomyces*, as described previously (33).

### Identification, purification, and structure elucidation of new compounds.

*Streptomyces* sp. strain YIM 121038 was cultured in 500 mL TSB medium plus apramycin at 30°C for 4 days on the shaker. The culture was centrifuged and the pellet was extracted with acetone. The extract was dried and separated by RP-HPLC (Phenomenex Luna 5-μm C_18_, 10 by 250 mm, 2.0 mL/min, 220 nm) with a gradient elution from 5% MeCN plus 0.1% formic acid (FA) to 100% MeCN plus 0.1% FA. The fraction containing compound 1 was further separated by RP-HPLC (Phenomenex Luna 5-μm phenyl-hexyl, 10 by 250 mm, 2.0 mL/min, 200 nm) with 30% MeCN plus 0.1% FA to obtain desertomycin X (compound 1) (1.0 mg). Fraction containing compound 2 was separated by RP-HPLC (Phenomenex Kinetex 5-μm C_18_, 10 by 250 mm, 2.0 mL/min, 200 nm) with 27% MeCN plus 0.1% FA to obtain desertomycin A (compound 2) (3.9 mg).

LC-ESIMS was performed on a Thermo Scientific Q Exactive mass spectrometer coupled to a Dionex Ultimate 3000 ultraperformance liquid chromatography (UPLC) system. NMR spectra were recorded on a Bruker Avance III spectrometer equipped with a cold probe at 500 MHz and 600 MHz for ^1^H NMR and 125 MHz and 150 MHz for ^13^C NMR at 298 K. Chemical shifts were referenced to the solvent peaks at δH 2.50 and δC 39.51 for DMSO-*d*_6_ and δH 3.31 and δC 49.15 for methanol-*d*_4_. NMR data are presented in Table S4A and B.

10.1128/mbio.01789-22.9 TABLE S4 (A) NMR data of desertomycin X (compound 1); (B) NMR data of desertomycin A (compound 2). Download Table S4, DOCX file, 0.02 MB. Copyright © 2022 Schneider et al. 2022 Schneider et al. https://creativecommons.org/licenses/by/4.0/ This content is distributed under the terms of the Creative Commons Attribution 4.0 International license.

To analyze the time-dependent production of desertomycin A and desertomycin X, *Streptomyces* sp. strain YIM 121038 was cultured in 20 mL TSB medium supplied with apramycin at 30°C for 6 days on the shaker. Two milliliters of culture was sampled daily with further separation of supernatant and pellet by centrifugation and freeze drying. Next, the supernatant was extracted with methanol and the pellet with 80% acetone.

### Antimicrobial activity testing.

Testing of antimicrobial activities of desertomycin A and X was done by disk diffusion assay using Bacillus subtilis 164, Escherichia coli DH5a, and Saccharomyces cerevisiae BY4742 as test organisms. Desertomycins were dissolved in DMSO to a stock solution of 8 mg/mL and tested in triplicate at final assay concentrations from 128 to 0.25 μg/mL. *In vitro* susceptibility for desertomycin A was determined at Medina Foundation (Granada, Spain) by the broth microdilution method following the Clinical and Laboratory Standards Institute protocol (34) using bacterial strains from the company’s strain collection (Table S4).

### Construction of the CsbC expression vectors.

Expression of codon-optimized gene *csbC* was carried out using the pET-30a(+) vector (Novagen). The synthetic *csbC* gene encoded the protein with 6×His tag on its N terminus, and was cloned into the pET30a(+) vector as a NdeI-BamHI fragment, yielding the pET-CsbC-S vector. For the experiments aimed at verifying enzyme activity *in vivo*, the vector pET-CsbC-S was modified by replacement of the gene Kan^r^ gene by the Amp^r^ gene. For this purpose, the Amp^r^ gene cassette (1,150 bp) was amplified from the pGEM-3Zf(+) vector with the primer set pGEM_Amp_fwd/pGEM_Amp_rev (supplemental material), and the PCR product was introduced into the 5.3-kb SmaI/PsiI fragment of the vector pET-CsbC-S and 4.7-kb SmaI/PsiI fragment of the vector pET30a(+) via Gibson assembly, yielding plasmids pA_CsbC-S and pA (negative control in assays). To prove the activity of CsbC *in vivo Streptomyces* cells, the pUWL_CsbCwt vector was generated; its construction was based on ligation of the amplified native gene *csbC* from YIM 121038 genomic DNA (gDNA) (designated below as *csbCwt*) and the pUWLoriT vector, over restriction enzymes MfeI/HindIII and EcoRI/HindIII, respectively.

### Heterologous expression of recombinant CsbC.

To determine the activity of the CsbC enzyme *in vitro*, it was expressed in Escherichia coli BL21(DE3) (Invitrogen, Carlsbad, CA, USA) cells harboring pET-CsbC vector. After overnight incubation at 37°C with kanamycin, a 5-mL overnight culture was used to inoculate 200 mL triconcentrated LB medium supplemented with kanamycin. The cultivation was carried out at 37°C. To initiate overexpression, the culture was induced with 0.5 mM final concentration of isopropyl-β-d-thiogalactopyranoside (IPTG) when it reached an optical density at 600 nm (OD_600_) of 1. After induction, the incubation was continued for 2 h at 25°C and overnight at 15°C at 220 rpm. Cells were harvested by centrifugation (15 min at 4,000 × rpm at 4°C).

The cell pellet was resuspended in 10 mL lysis buffer (50 mM potassium phosphate buffer [pH 8], 300 mM NaCl, 10 mM imidazole, 1 mM dithiothreitol [DTT], 100 μg/mL lysozyme), and the cells were disrupted using a Bioruptor Plus (amplitude, 30%; cycle 3) for 10 min. The sonicated samples were centrifuged for 30 min at 10,000 rpm at 4°C, and the supernatant was filtrated through a 2-μm-pore filter to remove cell debris. CsbC with a fused 6×His tag was purified via Ni-nitrilotriacetic acid (NTA) affinity chromatography following the manufacturer’s protocol (Qiagen), with the following exceptions for the buffers: washing buffer (50 mM potassium phosphate buffer [pH 8], 300 mM NaCl, 20 mM imidazole, 1 mM DTT), elution buffer (50 mM potassium phosphate buffer [pH 8]), and dialysis buffer (50 mM Tris/HCl [pH 8], 50 mM NaCl). The purity of recombinant CsbC and its concentration were determined by SDS-PAGE and the Bradford assay, respectively.

### Assays for CsbC activity *in vitro*.

The assay for CsbC activity was carried out in 50 mM Tris-HCl (pH 8) containing 25 mM succinyl-CoA sodium salt (Sigma-Aldrich) and various compounds as acyl group acceptors. The following acceptor compounds were used: 4 mM putrescine, 4 mM ornithine, 0.4 mM desertomycin A, ethionamide, colistin, vancomycin, gentamicin, daptomycin, tetracycline, kanamycin, ampicillin, and 0.2 mM trimethoprim. Activity of CsbC with substrates kanamycin, ampicillin, and acetyl-CoA was also investigated. The total reaction mixture of 100 μL was incubated 2 h at 30°C, and the reaction was terminated by addition of 0.5 μL formic acid. Negative-control samples were prepared in the same way, except that formic acid was added to the mixture before substrate to deactivate CsbC. After the reaction was terminated, 50 μL of reaction mixture was applied directly onto the disc for bioassays, and the remaining 50 μL was analyzed with LC-MS/MS.

### Generation and analysis of enzyme kinetics data.

To study the kinetics of CsbC, a 50-μL reaction mixture was prepared, containing 50 mM Tris-HCl, 25 mM succinyl-CoA, 2 mg/mL CsbC, and various ampicillin (Amp) concentrations (0 to 8.1 mM). Reaction mixtures were incubated at 30°C and terminated at different time points (0, 5, 10, 30, 60, 90, and 120 min) by addition of 0.5% formic acid. Denatured protein was removed by centrifugation (13,000 rpm, 15 min), and formation of succinylated ampicillin (Amp-S) was monitored by HPLC (with the same column and conditions as for LC-MS/MS analysis) by injection of a 5-μL sample. The peak area for Amp-S at ca. 18 min and 190 nm (Area_unknown_) was identified with LabSolutions version 5.97 software (Shimadzu Corporations). As a standard concentration (*C*_known_), the sample with 0.2 mmol Amp was used. In this reaction, according to the HPLC chromatogram, all Amp was succinylated after 30 min of incubation. Thus, the measured area in HPLC at 190 nm (Area_known_) and known standard concentrations (0.2 mmol) were used in the following equation to determine *C*_unknown_: *C*_unknown_ = (Area_unknown_/Area_known_) × *C*_known_.

### LC-MS analyses.

LC-MS/MS analyses of the reaction mixtures were performed on a Vanquish Horizon UHPLC system (Thermo Fisher Scientific) coupled to the ESI source of an LTQ Orbitrap Velos mass spectrometer (Thermo Fisher Scientific). Separation was carried out on an Acclaim 120 C_18_ column (2.1 by 150 mm, 3 μm) (Thermo Fisher Scientific) using water and acetonitrile, both modified with 0.1% formic acid, as mobile phases A and B, respectively. Depending on the polarity of the substrate, the sample components were separated and eluted with either of the following two gradient programs: (i) a linear gradient from 5% to 95% B in 45 min, followed by an isocratic column cleaning (9.5 min at 95% B) and reequilibration step (10 min at 5% B); or (ii) a gradient starting with an isocratic step at 3% B for 1 min, followed by a linear gradient from 3% to 30% B in 9 min, an increase to 95% B in 0.5 min, and finally an isocratic column cleaning (5 min at 95% B) and reequilibration step (9 min at 3% B). The flow rates were 0.45 and 0.50 mL/min, respectively, and the column oven temperature was set to 25°C. High-resolution ESI-MS (HR-ESIMS) spectra were recorded in positive-ion mode with an FT resolution of 60.000. High- or low-resolution ESIMS/MS spectra of the three most intense precursor ions in each MS^1^ spectrum were obtained in automated materials-dependent acquisition mode using helium as the collision gas and the following settings: activation type, CID; isolation width, Δ*m*/*z *=* *3; normalized collision energy, 35.0; activation Q, 0.250; and activation time, 30 ms.

LC-MS/MS data for the cell pellet and supernatant extracts of the *Streptomyces* sp. strain YIM 121038 cultures were obtained on a Vanquish Horizon UHPLC system coupled to a timsTOF fleX mass spectrometer (Bruker Daltonics) under similar conditions to those described above.

### CsbC activity *in vivo*.

To test an *in vivo* activity of CsbC, the experiments were carried with E. coli BL21(DE3) and S. lividans TK24 strains. Single colonies of E. coli BL21 strains carrying *csbC*-expressing vectors pET-CsbC or pA-CsbC and control vector pET30a(+) or pA were inoculated in LB containing Kan for pET constructs or Amp for pA constructs and incubated for 18 h at 37°C. One milliliter of overnight cultures was added into the 250 mL LB supplemented with Kan/Amp and incubated at 37°C up to an OD_600_ of 0.25. Next, samples were divided, and some were induced with 0.1 mM IPTG and incubated at 20°C for 1 h (pET constructs) or at 30°C for 30 min (pA vectors). After induction, some samples containing pA vectors were supplemented with 5 μg/mL Kan. After 24 h of incubation, 2 mL of the cultures was harvested, the obtained cell pellet was resuspended in 1 mL lysis buffer, and the cells were disrupted by sonication. The production of CsbC was determined by Western blotting with 15 μL filtered extract using an antibody specific for the 6×His tag.

After induction, different concentrations of Amp (0 to 50 μg/mL) were added to the cultures harboring pET30a(+) and pET-CsbC vectors with subsequent incubation for 18 h at 20°C and determination of the final OD_600_.

Fifty microliters each of spore suspension of S. lividans/pUWL-*csbC* and S. lividans/pUWLoriT strains was inoculated in YEME medium (35) supplemented with 30 μg/mL thiostrepton and kanamycin (0, 1, 2.5, 5, and 7 μg/mL) and incubated for 3 days at 28°C at 220 rpm. In parallel, 50 μL of spore suspension were plated on SFM agar plates with the same antibiotic concentrations as for liquid YEME medium and incubated at 28°C.

### Data availability.

This genome project has been deposited in DDBJ/ENA/GenBank under accession no. CP030771.1 (chromosome) and CP030772.1 (plasmid pSSP121038). The sequence of the 16S rRNA gene fragment of genomic DNA from YIM 121038 has been deposited in GenBank under accession no. MH429786.

## References

[1] Baltz RH. 2021. Genome mining for drug discovery: progress at the front end. J Ind Microbiol Biotechnol 48:kuab044. doi:10.1093/jimb/kuab044. 34279640 PMC8788784

[2] Al-Shaibani MM, Radin Mohamed RMS, Sidik NM, Enshasy HAE, Al-Gheethi A, Noman E, Al-Mekhlafi NA, Zin NM. 2021. Biodiversity of secondary metabolites compounds isolated from phylum Actinobacteria and its therapeutic applications. Molecules 26:4504. doi:10.3390/molecules26154504. 34361657 PMC8347454

[3] Cundliffe E. 1989. How antibiotic-producing organisms avoid suicide. Annu Rev Microbiol 43:207–233. (1989). doi:10.1146/annurev.mi.43.100189.001231. 2679354

[4] Graham MY, Weisblum B. 1979. 23S ribosomal ribonucleic acid of macrolide-producing streptomycetes contains methylated adenine. J Bacteriol 137:1464–1467. doi:10.1128/jb.137.3.1464-1467.1979. 438126 PMC218341

[5] Ohnuki T, Katoh T, Imanaka T, Aiba S. 1985. Molecular cloning of tetracycline resistance genes from *Streptomyces rimosus* in *Streptomyces griseus* and characterization of the cloned genes. J Bacteriol 161:1010–1016. doi:10.1128/jb.161.3.1010-1016.1985. 2982781 PMC214999

[6] Nguyen F, Starosta AL, Arenz S, Sohmen D, Dönhöfer A, Wilson DN. 2014. Tetracycline antibiotics and resistance mechanisms. Biol Chem 395:559–575. doi:10.1515/hsz-2013-0292. 24497223

[7] Favrot L, Blanchard JS, Vergnolle O. 2016. Bacterial GCN5-related N-acetyltransferases: from resistance to regulation. Biochemistry 55:989–1002. doi:10.1021/acs.biochem.5b01269. 26818562 PMC4795176

[8] Farazi TA, Waksman G, Gordon JI. 2001. Structures of *Saccharomyces cerevisiae N*-myristoyltransferase with bound myristoyl CoA and peptide provide insights about substrate recognition and catalysis. Biochemistry 40:6335–6343. doi:10.1021/bi0101401. 11371195

[9] Vetting MW, Errey JC, Blanchard JS. 2008. Rv0802c from *Mycobacterium tuberculosis*: the first structure of a succinyltransferase with the GNAT fold. Acta Crystallogr Sect F Struct Biol Cryst Commun 64:978–985. doi:10.1107/S1744309108031679. PMC2581710 18997321

[10] Anand C, Santoshi M, Singh PR, Nagaraja V. 2021. Rv0802c is an acyltransferase that succinylates and acetylates *Mycobacterium tuberculosis* nucleoid-associated protein HU. Microbiology (Reading). doi:10.1099/mic.0.001058. 34224344

[11] Davies J. 1994. Inactivation of antibiotics and the dissemination of resistance genes. Science 264:375–382. doi:10.1126/science.8153624. 8153624

[12] Matsuhashi Y, Murakami T, Nojiri C, Toyama H, Anzai H, Nagaoka K. 1985. Mechanisms of aminoglycosideresistance of Streptomyces harboring resistant genes obtained from antibiotic-producers. J Antibiot (Tokyo) 38:279–282. doi:10.7164/antibiotics.38.279. 3838980

[13] Kharel MK, Subba B, Basnet DB, Woo JS, Lee HC, Liou K, Sohng JK. 2004. A gene cluster for biosynthesis of kanamycin from *Streptomyces kanamyceticus*: comparison with gentamicin biosynthetic gene cluster. Arch Biochem Biophys 429:204–214. doi:10.1016/j.abb.2004.06.009. 15313224

[14] Blin K, Shaw S, Kloosterman AM, Charlop-Powers Z, van Wezel GP, Medema MH, Weber T. 2021. antiSMASH 6.0: improving cluster detection and comparison capabilities. Nucleic Acids Res 49:W29–W35. doi:10.1093/nar/gkab335. 33978755 PMC8262755

[15] Bax A, Aszalos A, Dinya Z, Sudo K. 1986. Structure elucidation of the antibiotic desertomycin through the use of new two-dimensional NMR techniques. J Am Chem Soc 108:8056–8063. doi:10.1021/ja00285a029.

[16] Hashimoto T, Kozone I, Hashimoto J, Suenaga H, Fujie M, Satoh N, Ikeda H, Shin-ya K. 2020. Identification, cloning and heterologous expression of biosynthetic gene cluster for desertomycin. J Antibiot 73:650–654. doi:10.1038/s41429-020-0319-0. 32457441

[17] Card GL, Peterson NA, Smith CA, Rupp B, Schick BM, Baker EN. 2005. The crystal structure of Rv1347c, a putative antibiotic resistance protein from *Mycobacterium tuberculosis*, reveals a GCN5-related fold and suggests an alternative function in siderophore biosynthesis. J Biol Chem 280:13978–13986. doi:10.1074/jbc.M413904200. 15695811

[18] Lorenzo VD, Neilands JB. 1986. Characterization of *iucA* and *iucC* genes of the aerobactin system of plasmid Co1V-K30 in *Escherichia coli*. J Bacteriol 167:350–355. doi:10.1128/jb.167.1.350-355.1986. 3087960 PMC212882

[19] Burrell M, Hanfrey CC, Kinch LN, Elliott KA, Michael AJ. 2012. Evolution of a novel lysine decarboxylase in siderophore biosynthesis: a novel lysine decarboxylase. Mol Microbiol 86:485–499. doi:10.1111/j.1365-2958.2012.08208.x. 22906379

[20] Waglechner N, Culp EJ, Wright GD. 17 March 2021, posting date. Ancient antibiotics, ancient resistance. EcoSal Plus 2021. doi:10.1128/ecosalplus.ESP-0027-2020. PMC11163840 33734062

[21] Pinheiro B, Petrov DP, Guo L, Martins GB, Bramkamp M, Jung K. 2021. Elongation factor P is required for EIIGlc translation in *Corynebacterium glutamicum* due to an essential polyproline motif. Mol Microbiol 115:320–331. doi:10.1111/mmi.14618. 33012080

[22] Miroux B, Walker JE. 1996. Over-production of proteins in *Escherichia coli*: mutant hosts that allow synthesis of some membrane proteins and globular proteins at high levels. J Mol Biol 260:289–298. doi:10.1006/jmbi.1996.0399. 8757792

[23] Jackowski S, Rock CO. 1986. Consequences of reduced intracellular coenzyme A content in *Escherichia coli*. J Bacteriol 166:866–871. doi:10.1128/jb.166.3.866-871.1986. 3519582 PMC215206

[24] Traxler MF, Kolter R. 2015. Natural products in soil microbe interactions and evolution. Nat Prod Rep 32:956–970. doi:10.1039/c5np00013k. 26000872

[25] Bredholdt H, Galatenko OA, Engelhardt K, Fjaervik E, Terekhova LP, Zotchev SB. 2007. Rare actinomycete bacteria from the shallow water sediments of the Trondheim fjord, Norway: isolation, diversity and biological activity. Environ Microbiol 9:2756–2764. doi:10.1111/j.1462-2920.2007.01387.x. 17922759

[26] Koren S, Walenz BP, Berlin K, Miller JR, Bergman NH, Phillippy AM. 2017. Canu: scalable and accurate long-read assembly via adaptive *k*-mer weighting and repeat separation. Genome Res 27:722–736. doi:10.1101/gr.215087.116. 28298431 PMC5411767

[27] Walker BJ, Abeel T, Shea T, Priest M, Abouelliel A, Sakthikumar S, Cuomo CA, Zeng Q, Wortman J, Young SK, Earl AM. 2014. Pilon: an integrated tool for comprehensive microbial variant detection and genome assembly improvement. PLoS One 9:e112963. doi:10.1371/journal.pone.0112963. 25409509 PMC4237348

[28] Gordon D, Green P. 2013. Consed: a graphical editor for next-generation sequencing. Bioinformatics 29:2936–2937. doi:10.1093/bioinformatics/btt515. 23995391 PMC3810858

[29] Seemann T. 2014. Prokka: rapid prokaryotic genome annotation. Bioinformatics 30:2068–2069. doi:10.1093/bioinformatics/btu153. 24642063

[30] Kumar S, Stecher G, Li M, Knyaz C, Tamura K. 2018. MEGA X: molecular evolutionary genetics analysis across computing platforms. Mol Biol Evol 35:1547–1549. doi:10.1093/molbev/msy096. 29722887 PMC5967553

[31] Schaffert L, Albersmeier A, Winkler A, Kalinowski J, Zotchev SB, Rückert C. 2016. Complete genome sequence of the actinomycete *Actinoalloteichus hymeniacidonis* type strain HPA 177T isolated from a marine sponge. Stand Genomic Sci 11:91. doi:10.1186/s40793-016-0213-3. 28031775 PMC5168871

[32] Sambrook J, Fritsch EF, Maniatis T. 1989. Molecular cloning: a laboratory manual, 2nd ed. Cold Spring Harbor Laboratory Press, Cold Spring Harbor, NY.

[33] Flett F, Mersinias V, Smith CP. 1997. High efficiency intergeneric conjugal transfer of plasmid DNA from *Escherichia coli* to methyl DNA-restricting streptomycetes. FEMS Microbiol Lett 155:223–229. doi:10.1111/j.1574-6968.1997.tb13882.x. 9351205

[34] Clinical and Laboratory Standards Institute. 2012. Methods for dilution antimicrobial susceptibility tests for bacteria that grow aerobically. M07-A9. Approved standard, 9th ed, vol 32. Clinical and Laboratory Standards Institute, Wayne, PA.

[35] Hopwood DA, Bibb MJ, Chater KF, Kieser T, Bruton CJ, Kieser HM, Lydiate DJ, Smith CP, Ward JM, Schrempf H. 1985. Genetic manipulation of *Streptomyces*: a laboratory manual. John Innes Foundation, Norwich, United Kingdom.

