# Supplementary material for: N-Succinyltransferase Encoded by a Cryptic Siderophore Biosynthesis Gene Cluster in Streptomyces Modifies Structurally Distinct Antibiotics
Source: mBio. 2022 Aug 30;13(5):e01789-22. doi: 10.1128/mbio.01789-22 (PMC9600172; doi:10.1128/mbio.01789-22)
Supplement: TABLE S1 [file mbio.01789-22-s0001.docx]

**Table S1**

| BGC no. | Cluster type | Best database cluster hit | Putative product |
| --- | --- | --- | --- |
| 1 | Nrps  T1pks  Blactam | *Streptomyces* sp. MNP-20  *Streptomyces* sp. MNP-20  Many *Streptomyces* spp. | NRS peptide  Polyketide  Clavams |
| 2 | Ladderane | *Streptomyces* sp. NRRL B-1347 | Metatricycloene |
| 3 | T3pks-T1pks  Terpene | *Streptomyces* sp. NRRL B-1347 | Polyketide |
| 4 | T1pks | *Streptomyces* sp. NRRL B-1347 | Polyketide |
| 5 | Lantipeptide | *Streptomyces* sp. NRRL B-1347 | Lantipeptide, Class III |
| 6 | Nrps-Transatpks-Otherks | *Streptomyces spectabilis* | NRS peptide-polyketide hybrid |
| 7 | T1pks  T1pks | *Streptomyces* sp. SM14  *Streptomyces* sp. MNP-20 | Bafilomycin (or its analogue)  Polyene macrolide |
| 8 | Nrps-Pks | *Streptomyces* sp. MNP-20 | NRS peptide-polyketide hybrid |
| 9 | Nrps | *Streptomyces bicolor* NRRL B-5348 | Complestatin |
| 10 | T3pks | *Streptomyces* sp. NRRL B-1347 | α-pyrone-like polyketide |
| 11 | Nrps | *Streptomyces* sp. MNP-20 | NRS peptide |
| 12 | Ectoine | *Streptomyces* sp. MNP-20 | Ectoine |
| 13 | Terpene | *Streptomyces* sp. MNP-20 | Terpenoid |
| 14 | T1pks | *Streptomyces* sp. MNP-20 | Polyketide |
| 15 | Nrps | *Streptomyces sp.* MNP-20 | Diketopiperazine |
| 16 | Melanin | *Streptomyces sp.* MNP-20 | Melanin |
| 17 | Lantipeptide | Actinobacteria bacterium OK074 | Lantipeptide, class I |
| 18 | Melanin | *Streptomyces sp.* MNP-20 | Melanin |
| 19 | Terpene | *Streptomyces sp.* MNP-20 | Albaflavenone |
| 20 | Siderophore | *Streptomyces sp.* MNP-20 | Siderophore |
| 21 | RiPP | *Streptomyces sp.* MNP-20 | RiPP |
| 22 | Lantipeptide  Nrps | *Streptomyces sp.* MNP-20  *Streptomyces sp.* MNP-20 | Lantipeptide, class III  kutzneride analogue |
| 23 | Redox-cofactor | *Streptomyces sp.* MNP-20 | Likely false |
| 24 | Terpene | *Streptomyces sp.* MNP-20 | Geosmin |
| 25 | Terpene | *Streptomyces sp.* MNP-20 | Hopanoids |
| 26 | T1pks | *Streptomyces sp.* MNP-20 | Undecylprodigiosin |
| 27 | T1pks | *Streptomyces sp.* MNP-20 | Neoaureothin |
| 28 | T1pks | *Streptomyces sp.* MNP-20 | Desertomycin |
| 29 | T1pks | *Streptomyces spectabilis* ATCC 27465:: *Streptomyces sp.* MNP-20 (hybrid cluster) | Streptovaricin |
| 30 | T3pks-Nrps | *Streptomyces* sp. WAC1420 | Balhimycin-like glycopeptide |
| 31 | Nrps | *Streptomyces sp.* MNP-20 | Himastatin analogues |
| 32 | Amglyccyclitol  Nrps | *Streptomyces sp.* MNP-20  *Streptomyces sp.* MNP-20 | Actinospectacin    NRS peptide |
| 33 | T1pks-Nrps  Lantipeptide | *Streptomyces sp.* MNP-20  *Streptomyces sp.* ISL-44 | NRS peptide-polyketide hybrid  Lantipeptide, class I |
| 34 | Nrps | *Streptomyces sp.* MNP-20 | Mirubactin-like siderophore |
| 35 | Lassopeptide | *Streptomyces sp.* MNP-20 | putative Class II lasso peptide |
| 36 | Nrps | *Streptomyces sp.* MNP-20 | Coelichelin |
| 37 | RiPP | *Streptomyces* sp. ND90 | Likely false |
| 38 | Terpene | *Streptomyces* sp. ND90 | 2-Methylisoborneol |
| 39 | Lantipeptide | *Streptomyces sp.* MNP-20 | Lantipeptide, class II |
| 40 | Nrps | *Streptomyces sp.* MNP-20 | NRS peptide |
| 41 | Nrps  Pks2 | *Streptomyces sp.* MNP-20  *Streptomyces sp.* MNP-20 | NRS peptide  Unusual fatty acids |
| 42 | Terpene | *Streptomyces triticisoli* | Isorenieratene-like carotenoid |
| 43 | Lassopeptide | Actinobacteria bacterium OK006 | putative class II lasso peptide |
| 44 | Terpene | No plausible hits | Terpenoid |
| 45 | Terpene | No plausible hits | Terpenoid |
| 46 | Nrps  PKSI | *Streptomyces sp.* NRRL B-1347  *Streptomyces sp.* 769 | Broken by transposases |
| 47 | T1pks  Transatpks  Terpene | PKSI – No plausible hits  Trans-AT PKS: *Streptomyces* sp. 769  TS1 – No plausible hits  TS2 – No plausible hits | 2 terpene synthases, Trans-AT PKS, PKSI clusters, transposases |
| 48 | PKSI  Terpene | PKS - No plausible hits  TS1 – Streptomyces  TS2 - cyanobacteria | - |
| 49 | Indole | No plausible hits | Indole |
| 50 | Terpene | No plausible hits | Terpenoid |
| 51 | Lantipeptide | *Streptomyces chartreusis* | SapB/AmfS family lantipeptide |
| 52 | Terpene | No plausible hits | Terpenoid |
| 53 | T3pks  PKSI  Blactam-T2pks | *Streptomyces sahachiroi*  No plausible hits  No plausible hits | Polyketide-clavaminate hybrid |

NRS – non-ribosomally synthesized
