# Supplementary material for: N-Succinyltransferase Encoded by a Cryptic Siderophore Biosynthesis Gene Cluster in Streptomyces Modifies Structurally Distinct Antibiotics
Source: mBio. 2022 Aug 30;13(5):e01789-22. doi: 10.1128/mbio.01789-22 (PMC9600172; doi:10.1128/mbio.01789-22)
Supplement: FIG S1 [file mbio.01789-22-s0005.pdf]

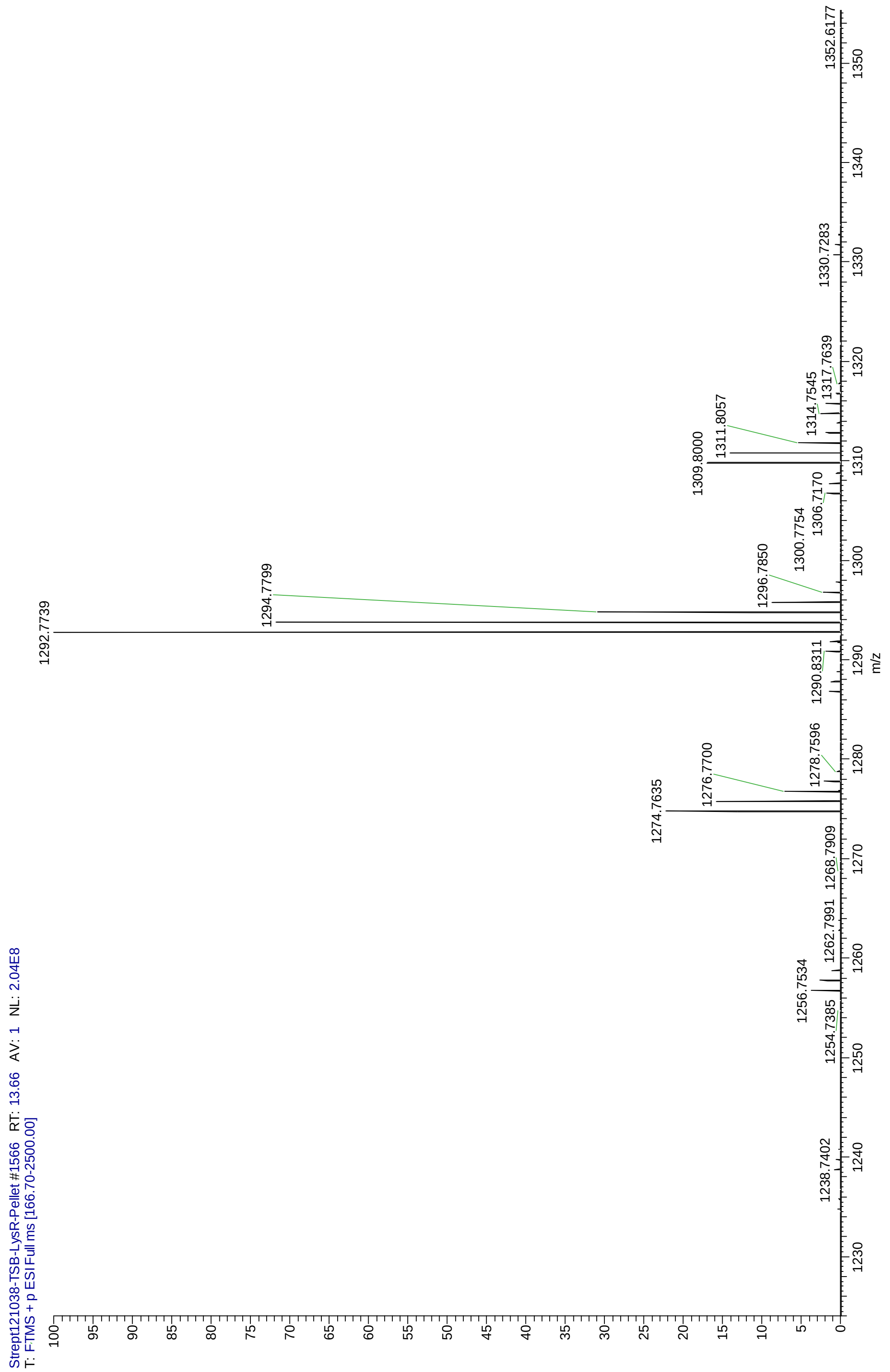
Strept121038-TSB-LysR-Pellet #1566 RT: 13.66 AV: 1 NL: 2.04E8
T: FTMS + p ESI Full ms [166.70-2500.00]
1292.7739
1294.7799
1296.7850
1300.7754
1290.8311
1274.7635
1276.7700
1278.7596
1268.7909
1262.7991
1256.7534
1254.7385
1238.7402
1309.8000
1311.8057
1306.7170
1314.7545
1317.7639
1330.7283
1352.6177
m/z

FIG S1A.

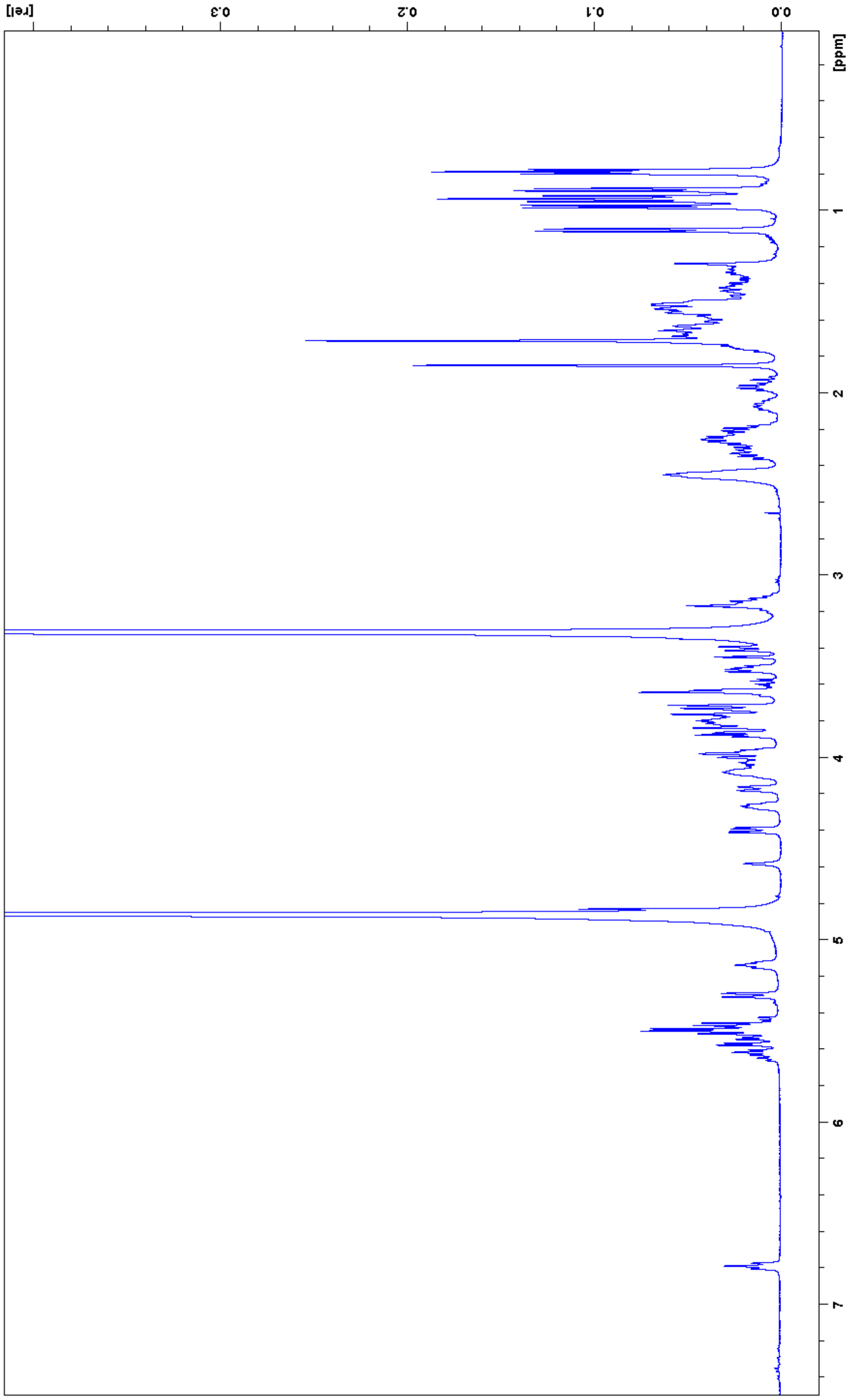

FIG S1B

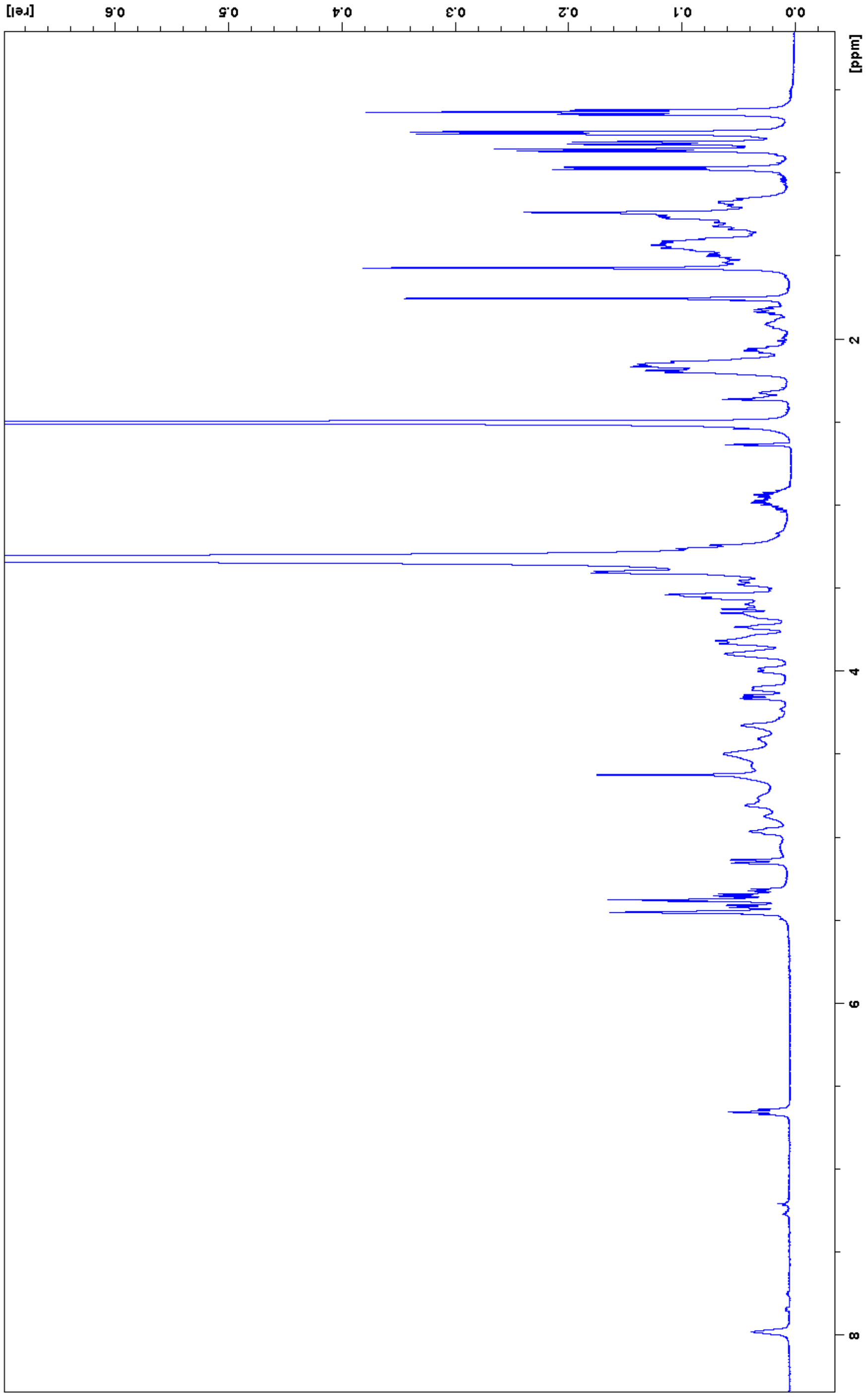

FIG S1C

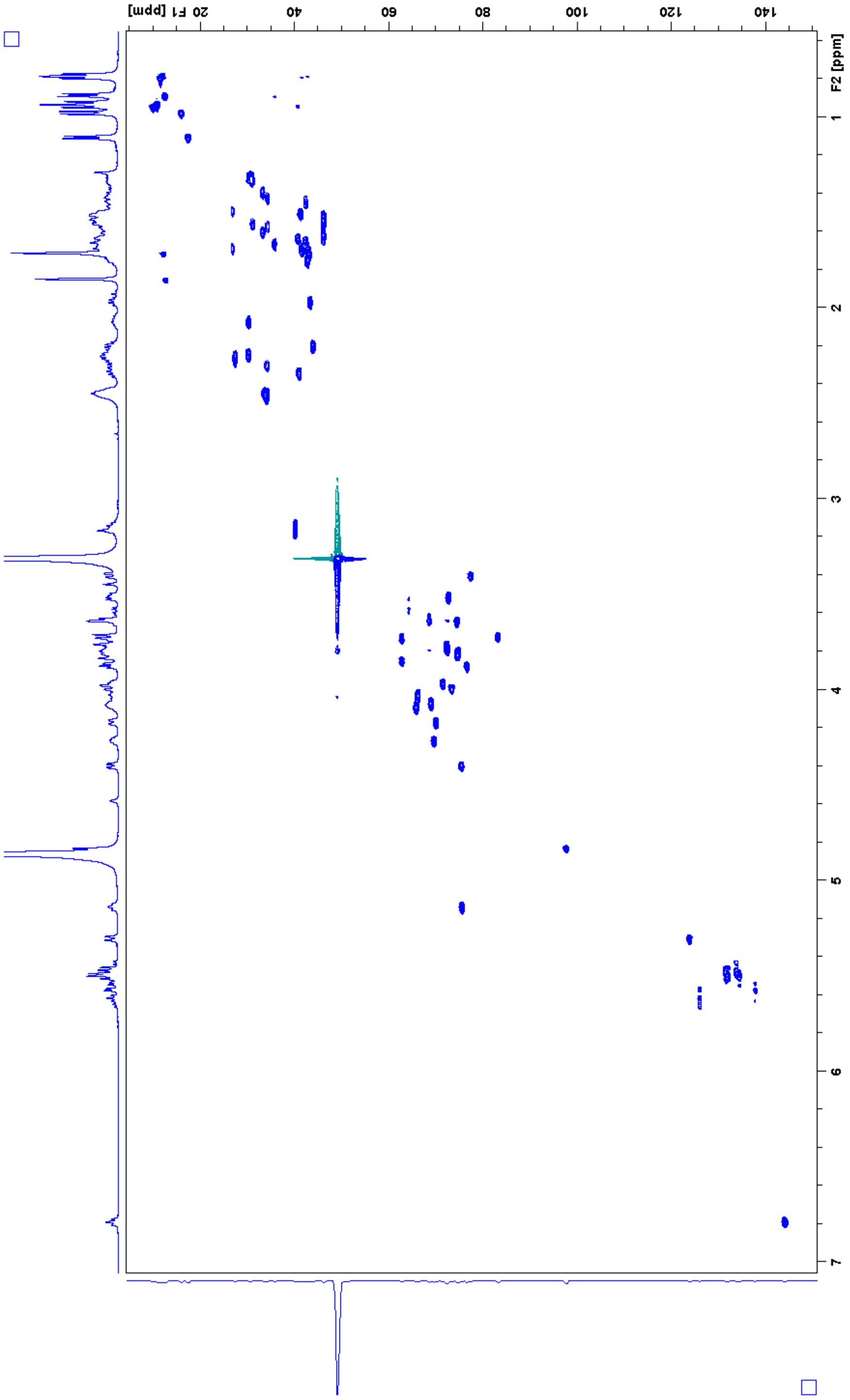

FIG S1D

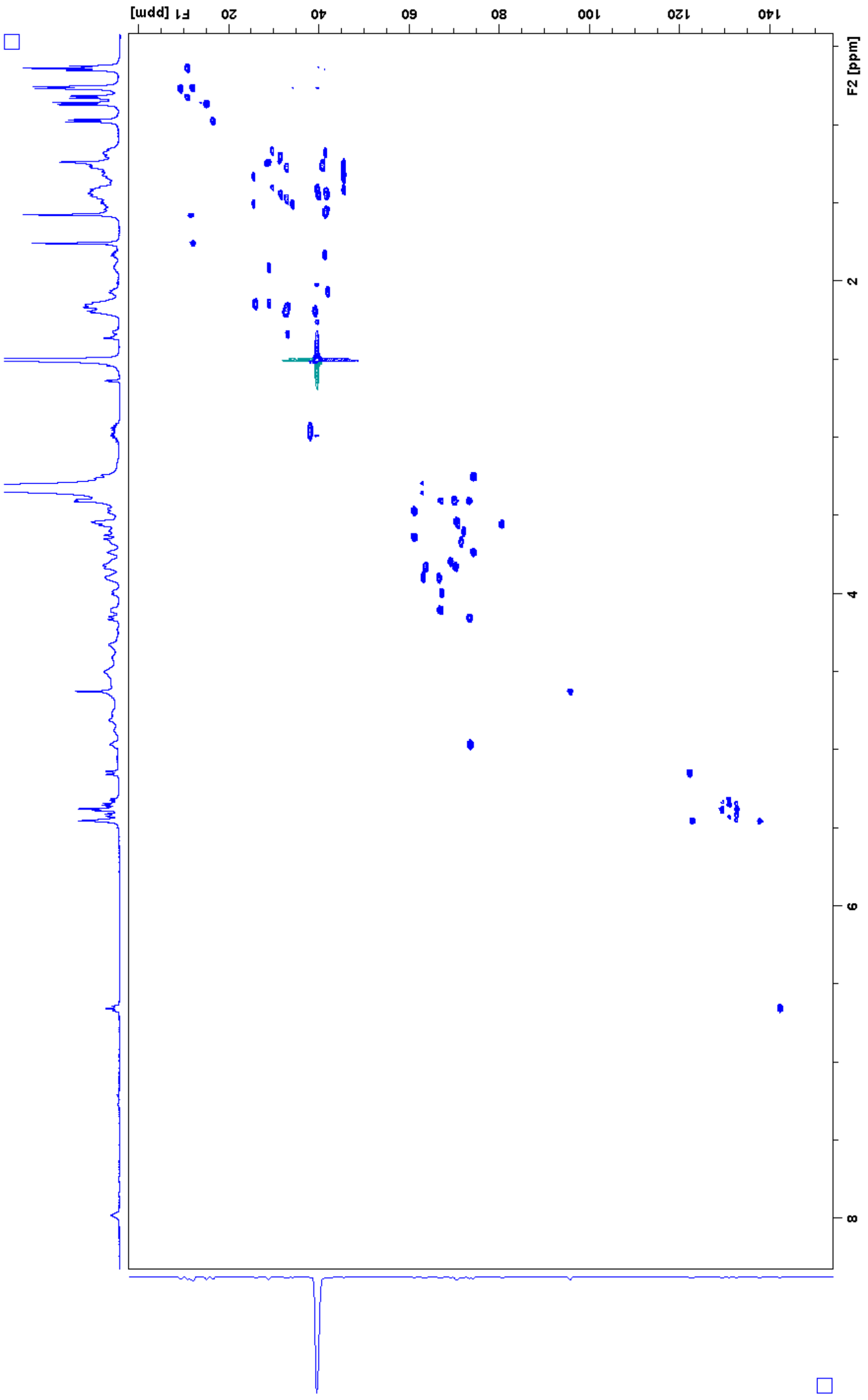

FIG S1E

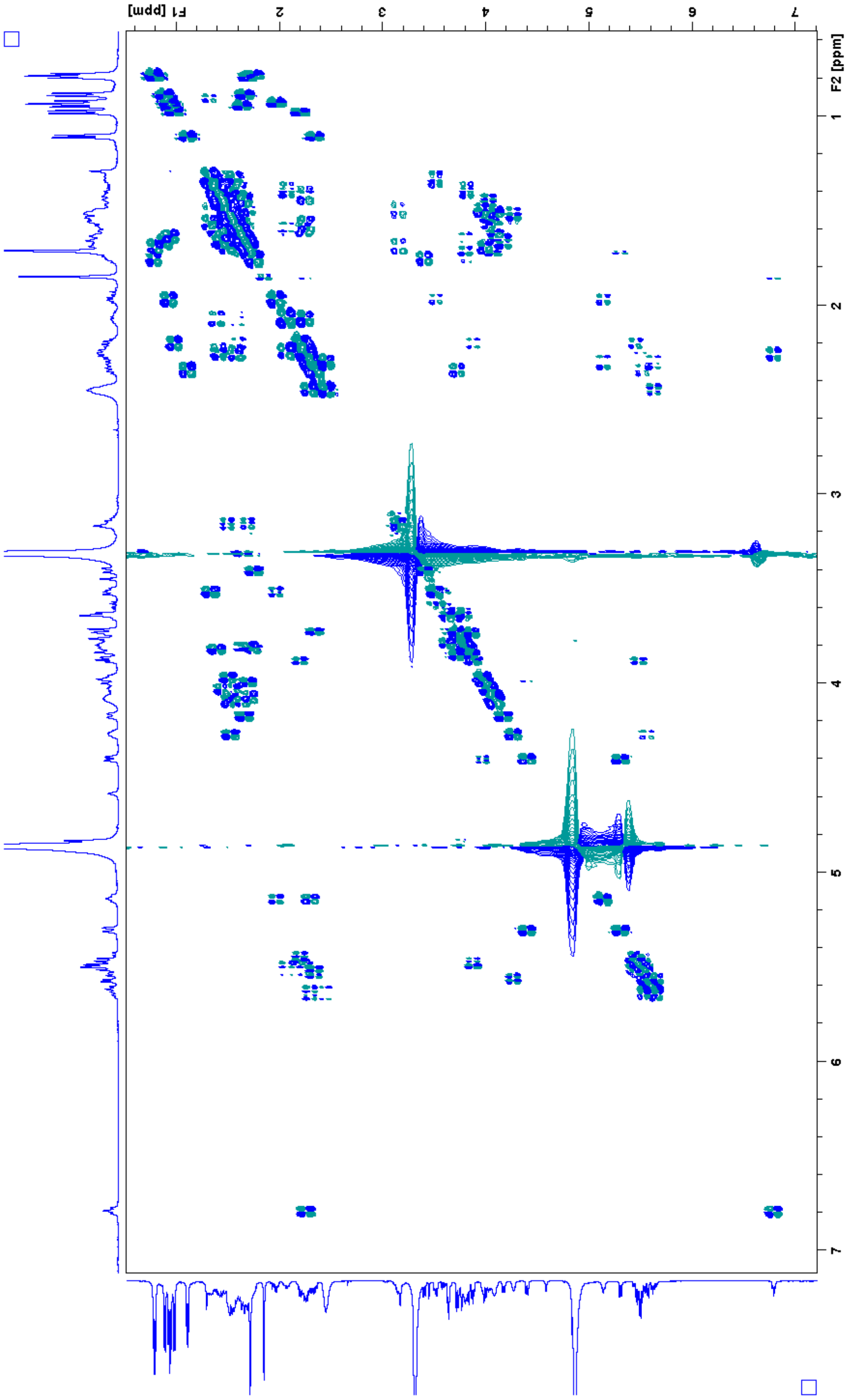

FIG S1F

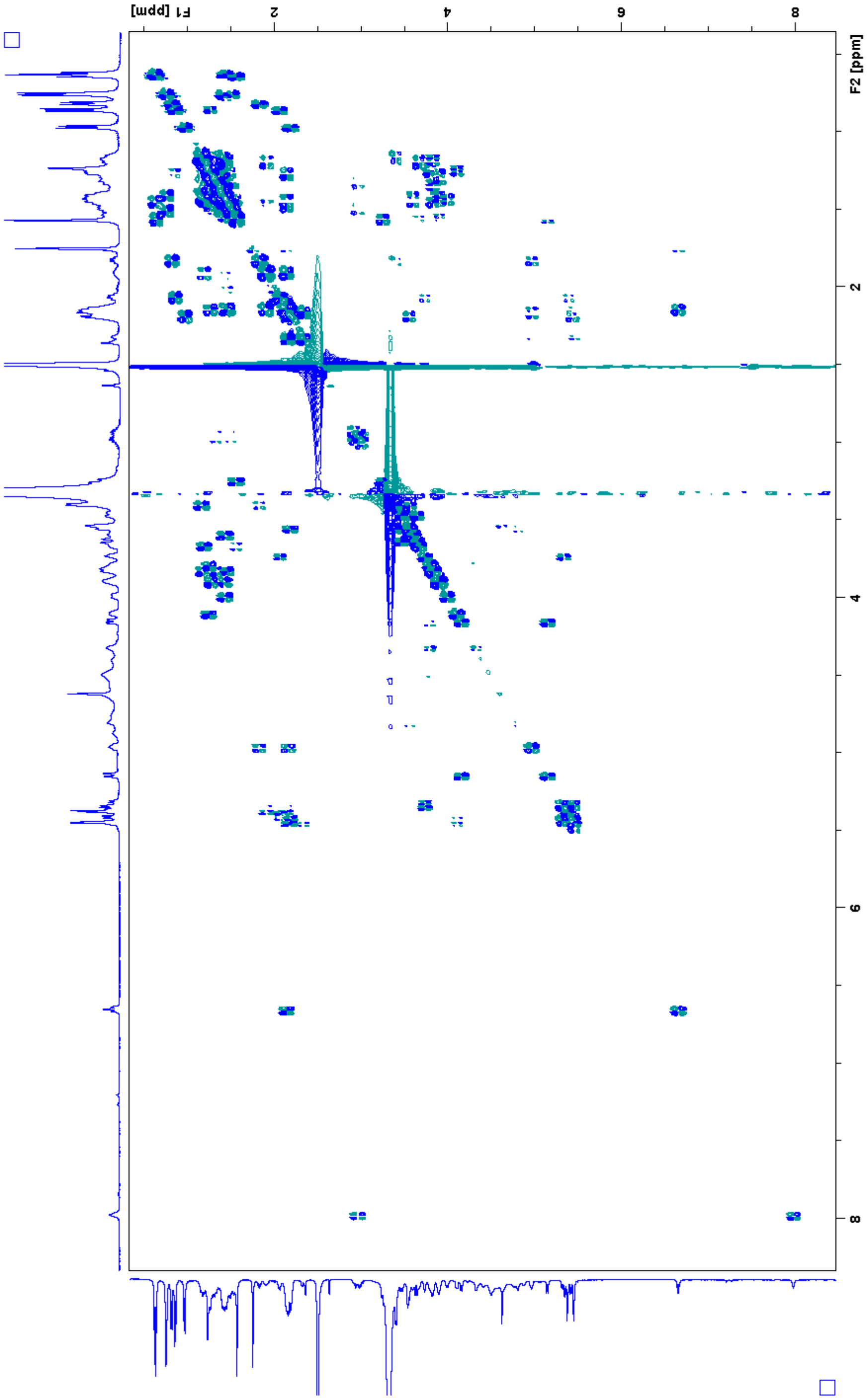

FIG S1G

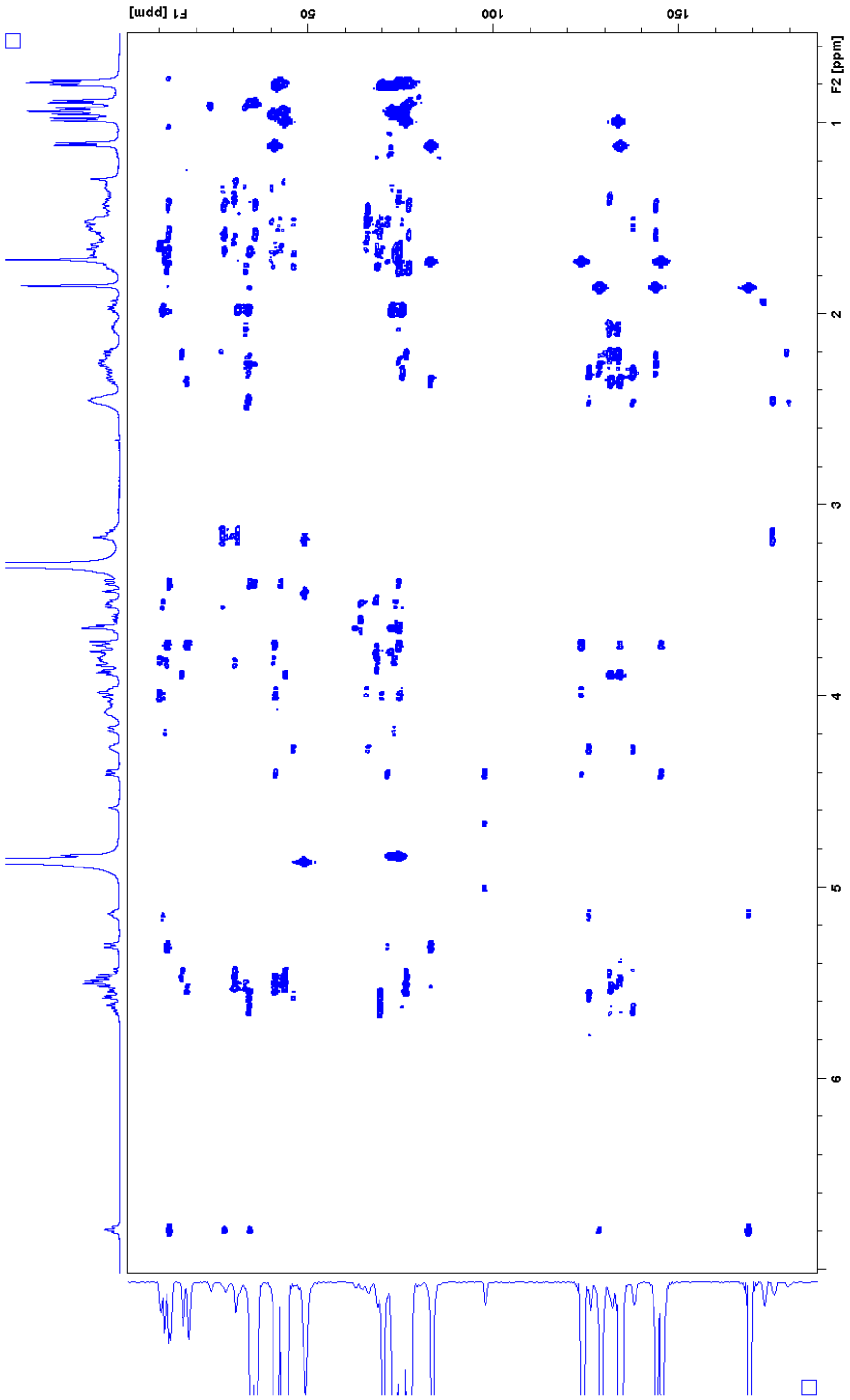

FIG S1H

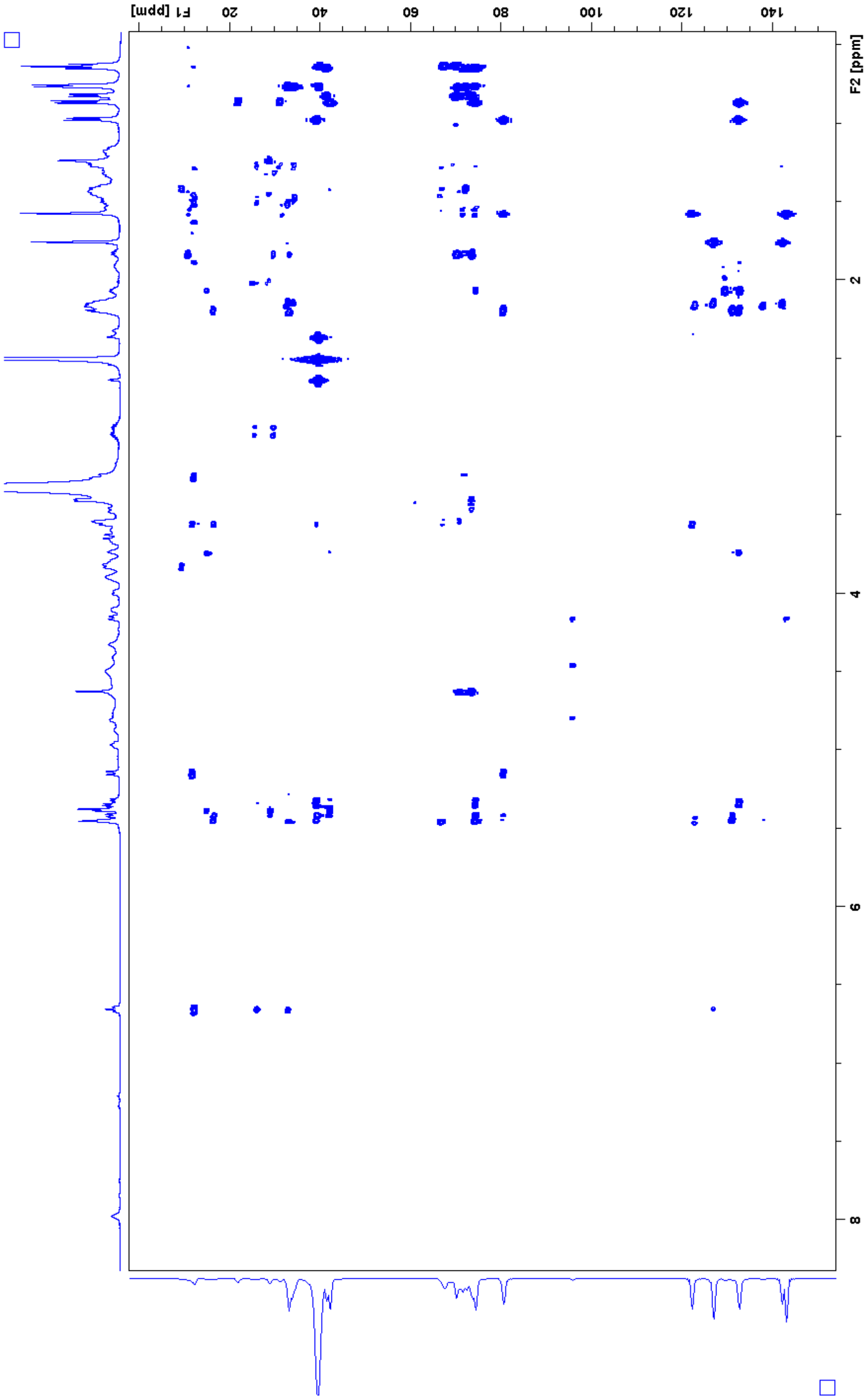

FIG S1I

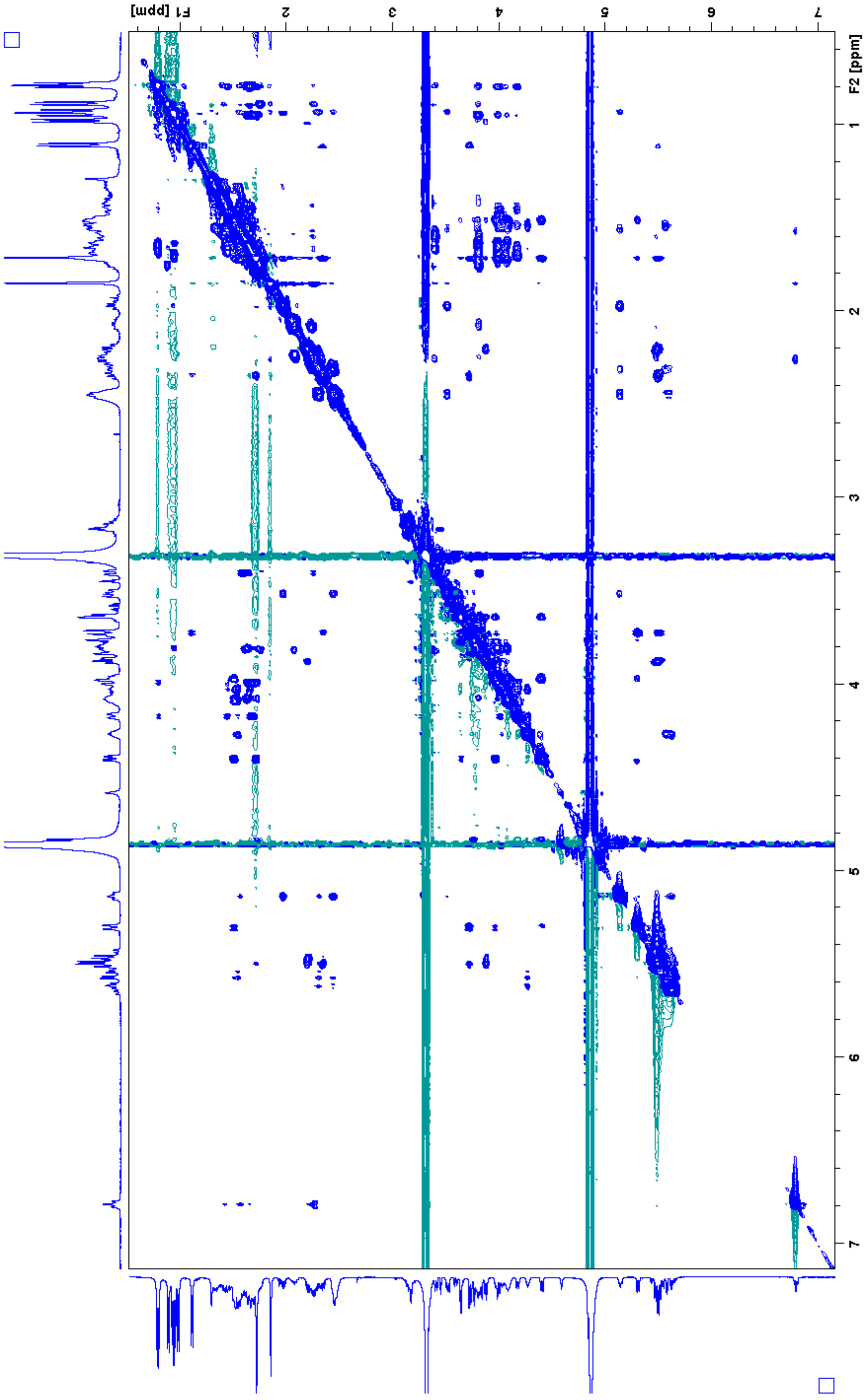

FIG S1J
