# Supplementary figures and images for: N-Succinyltransferase Encoded by a Cryptic Siderophore Biosynthesis Gene Cluster in Streptomyces Modifies Structurally Distinct Antibiotics
Source: mBio. 2022 Aug 30;13(5):e01789-22. doi: 10.1128/mbio.01789-22 (PMC9600172; doi:10.1128/mbio.01789-22)

LyR-Gra45-1 #1476 RT: 12.87 AV: 1 NL: 9.02E6  
T: FTMS + pESI Full ms [166.70-2500.00]

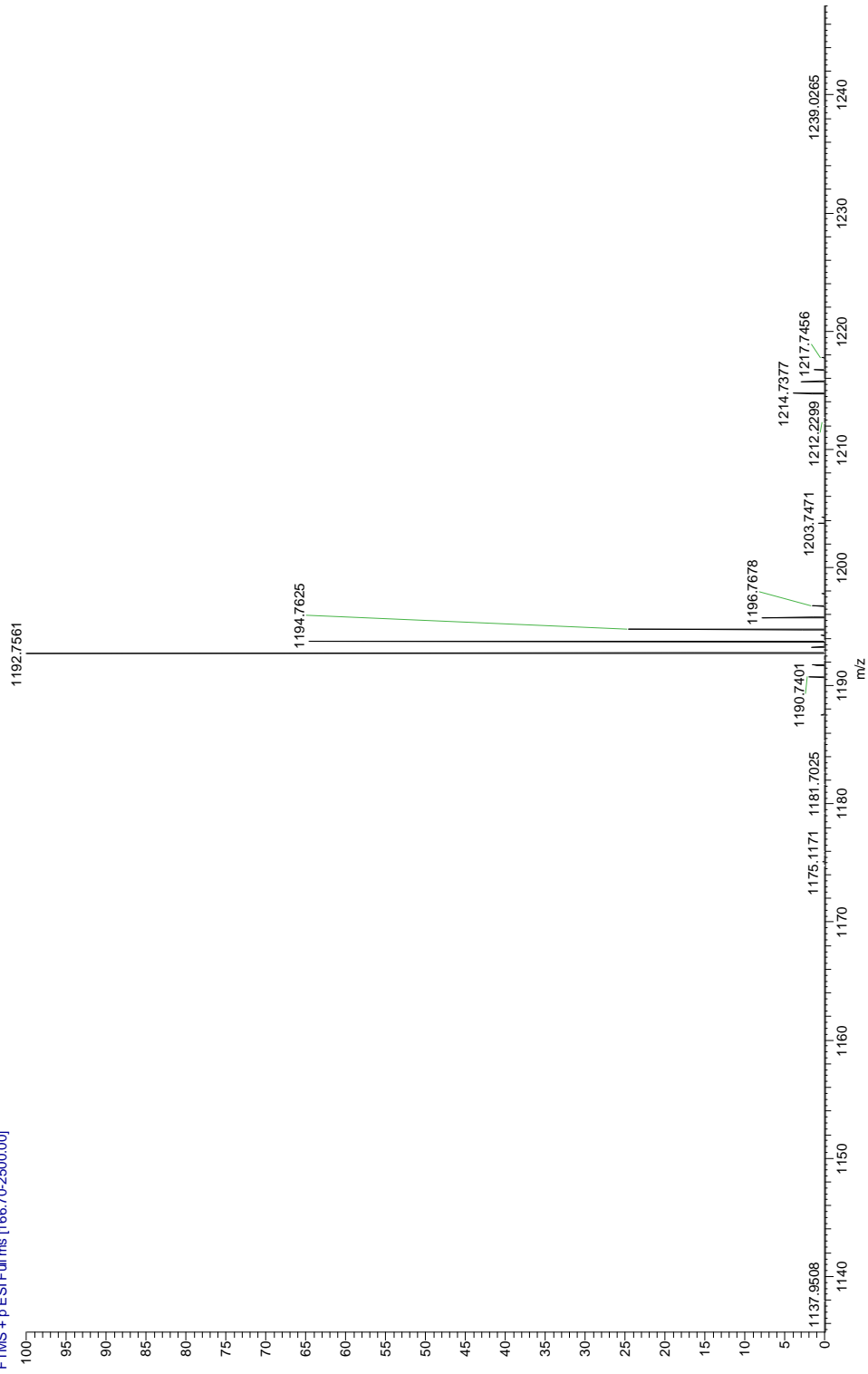

FIG S2A

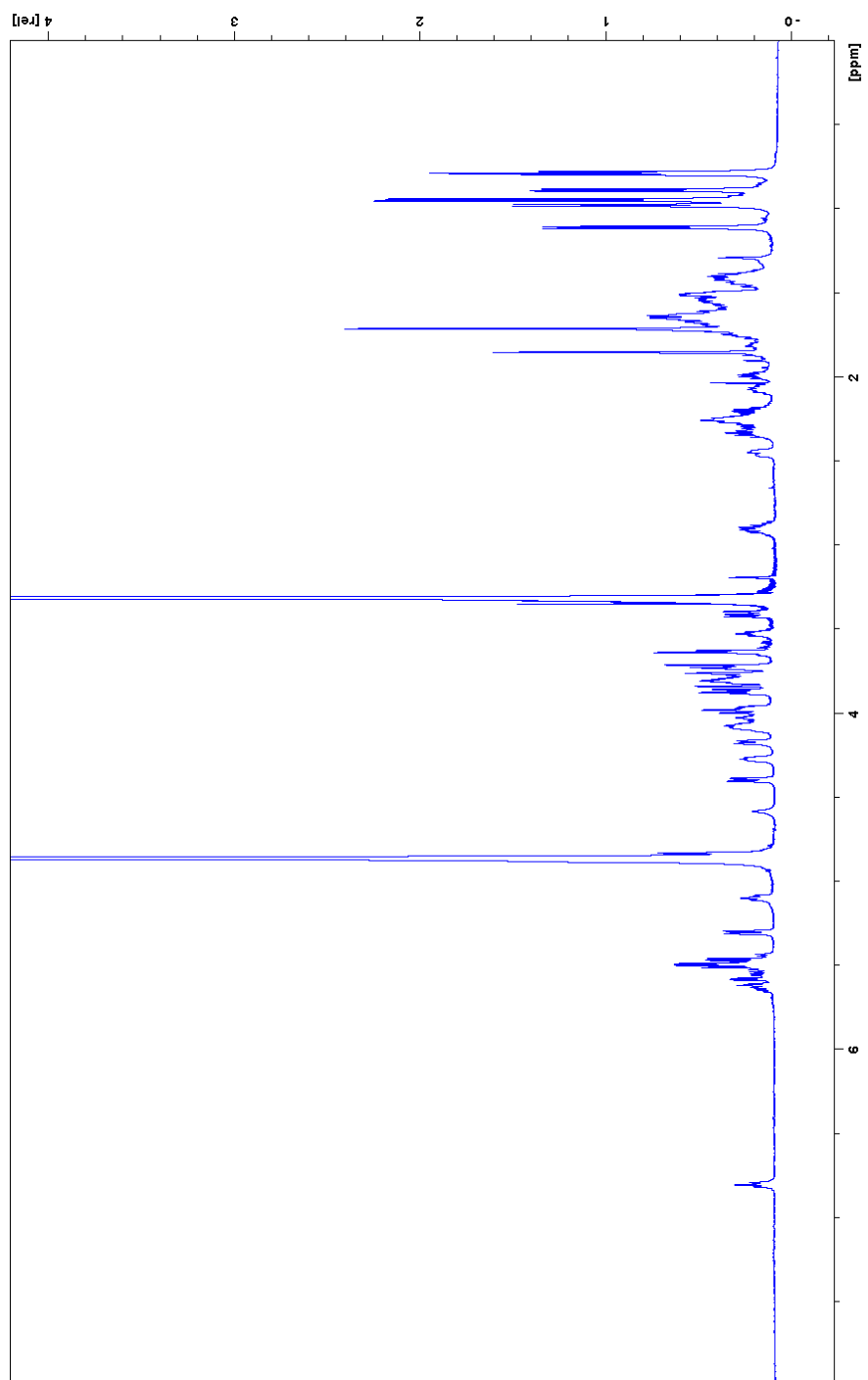

**FIG S2B**

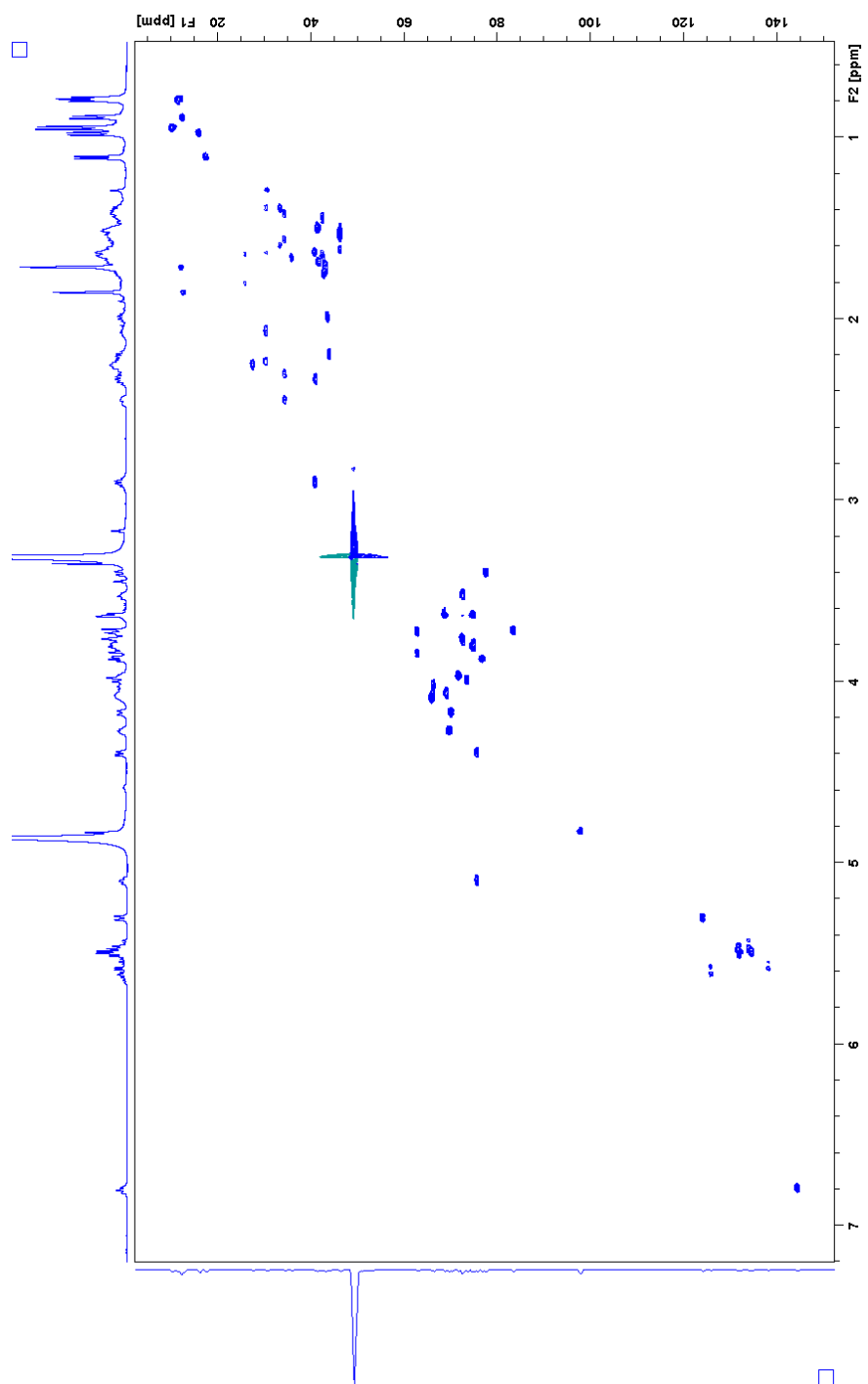

**FIG S2C**

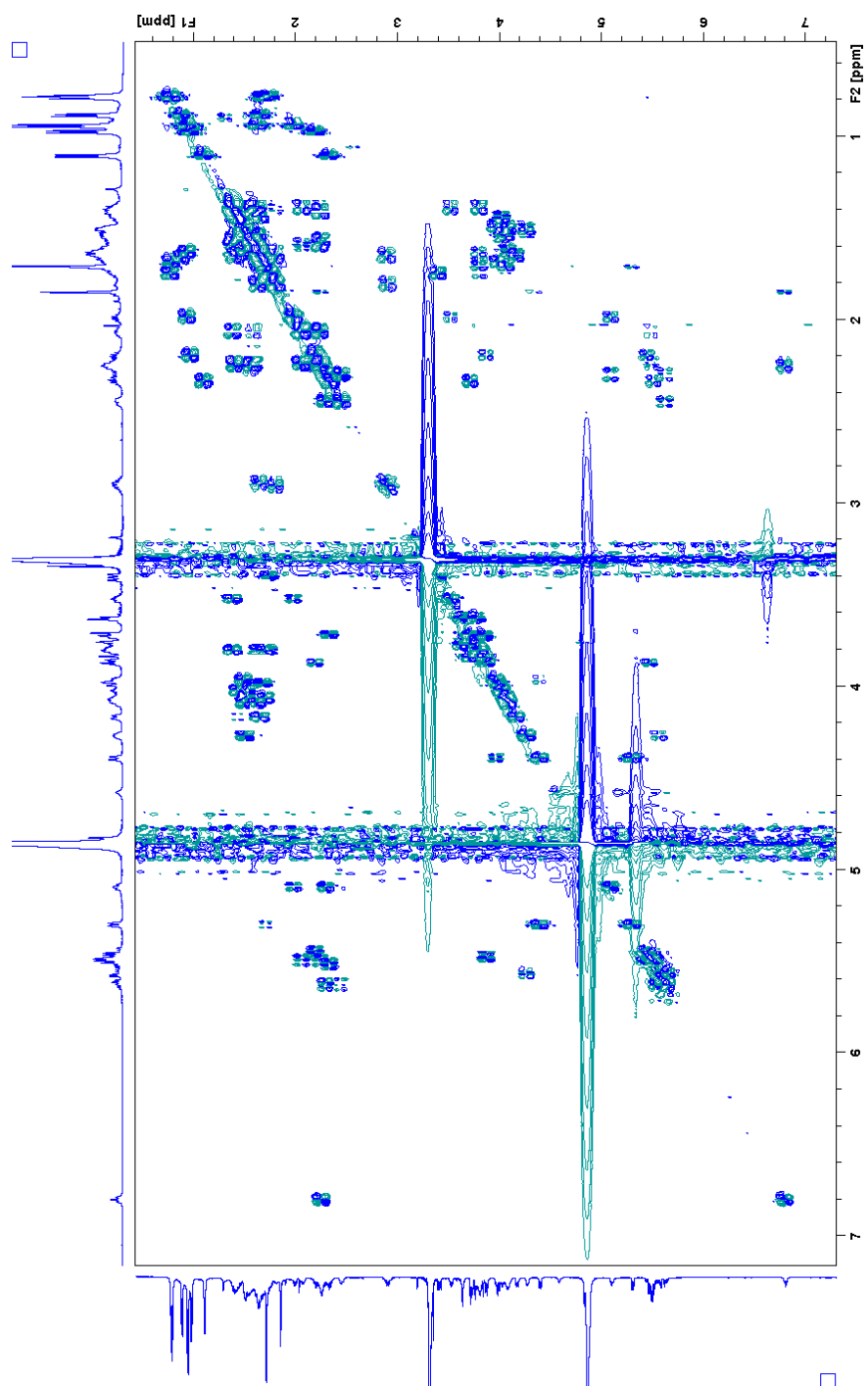

**FIG S2D**

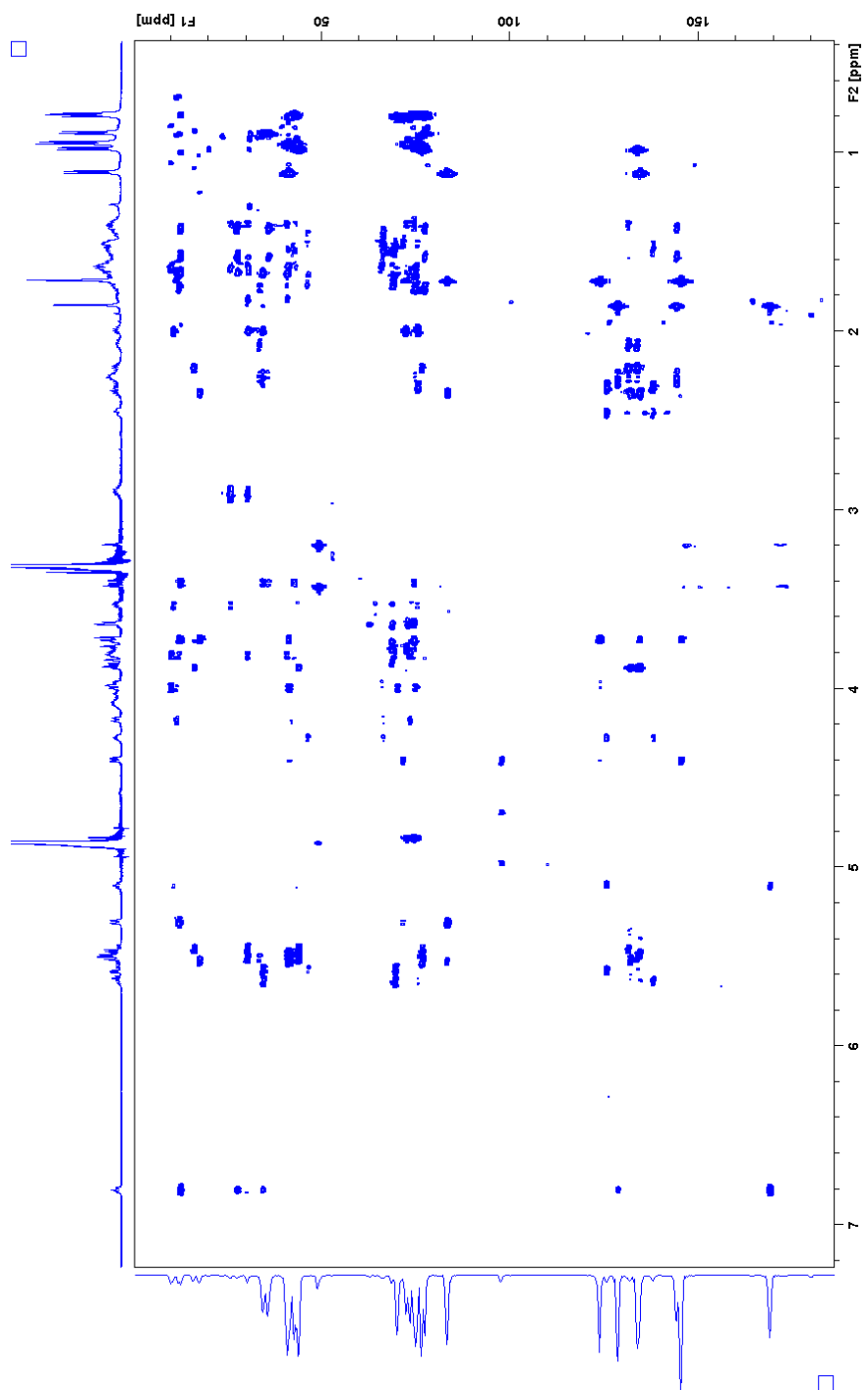

**FIG S2E**

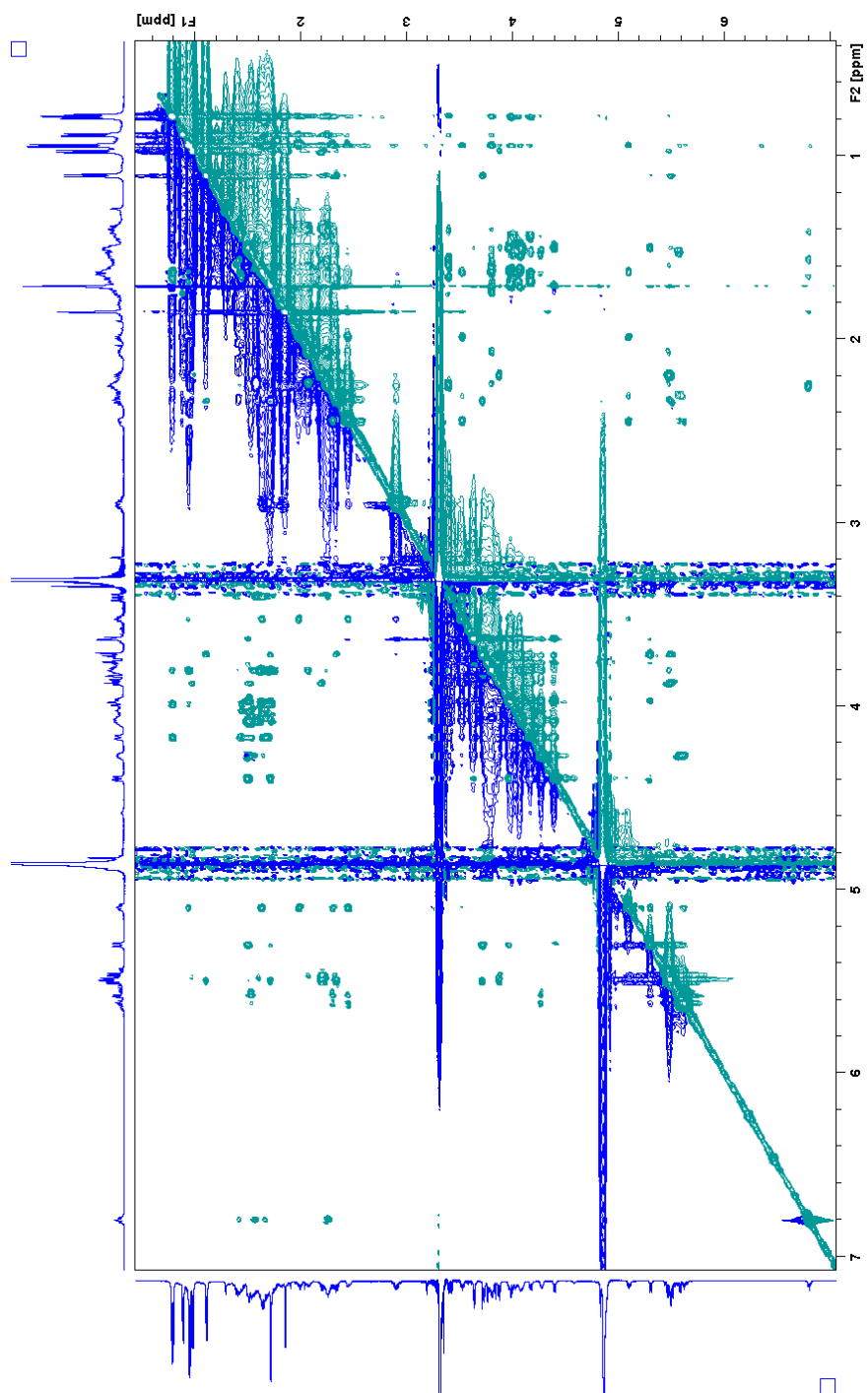

**FIG S2F**

Supplement: FIG S2 [file mbio.01789-22-s0006.pdf]

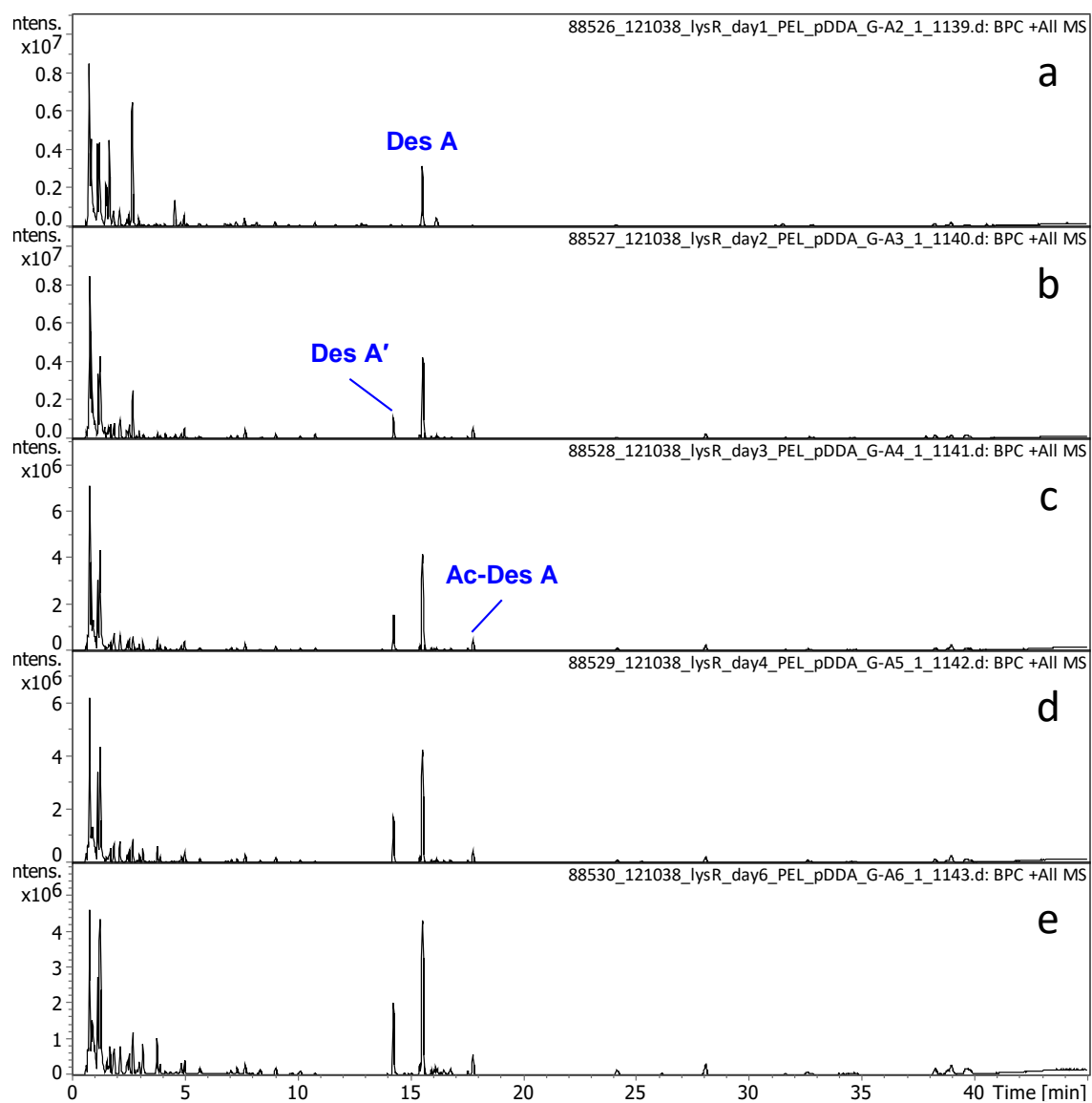

**FIG S4A**

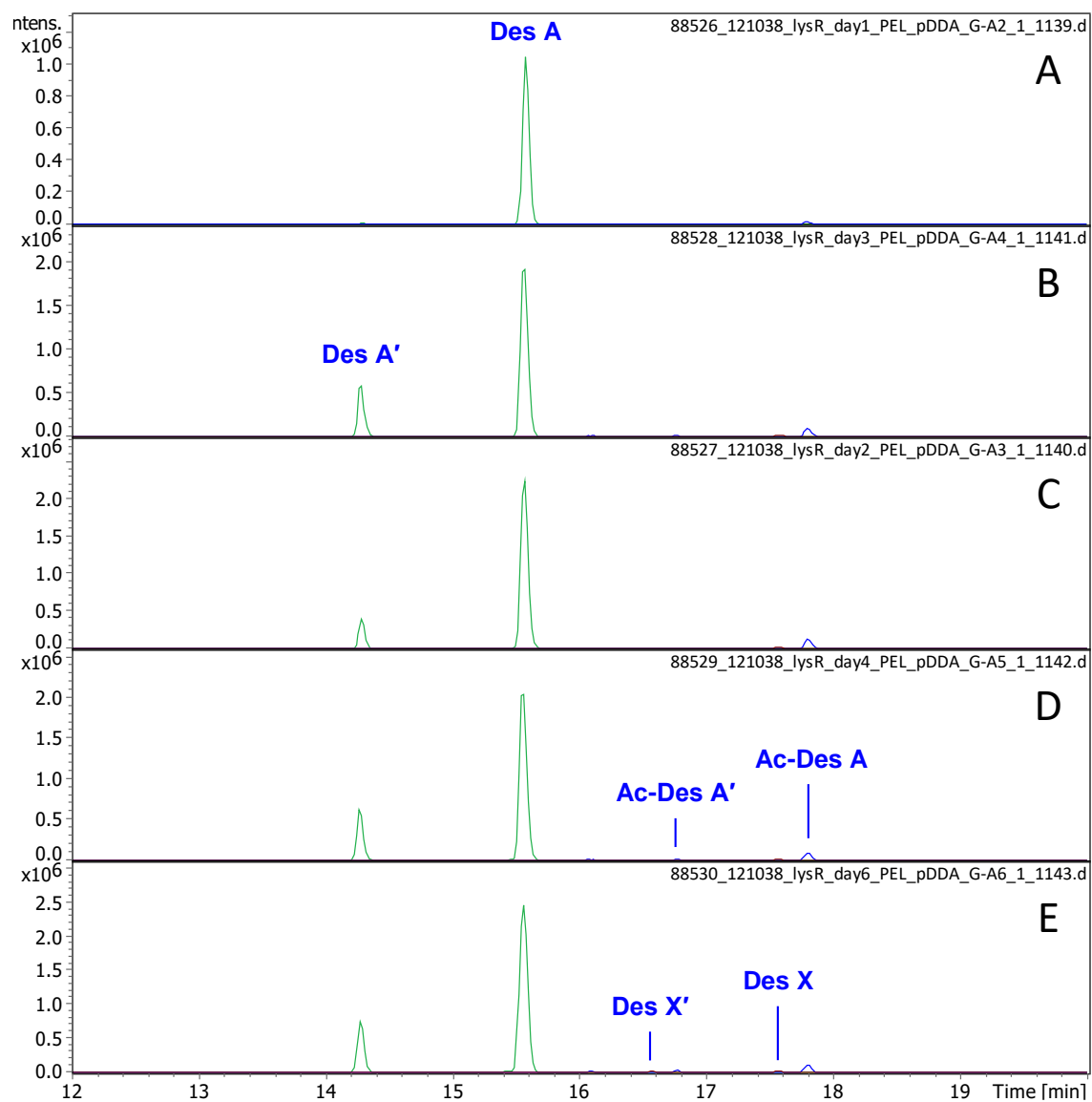

**FIG S4B**

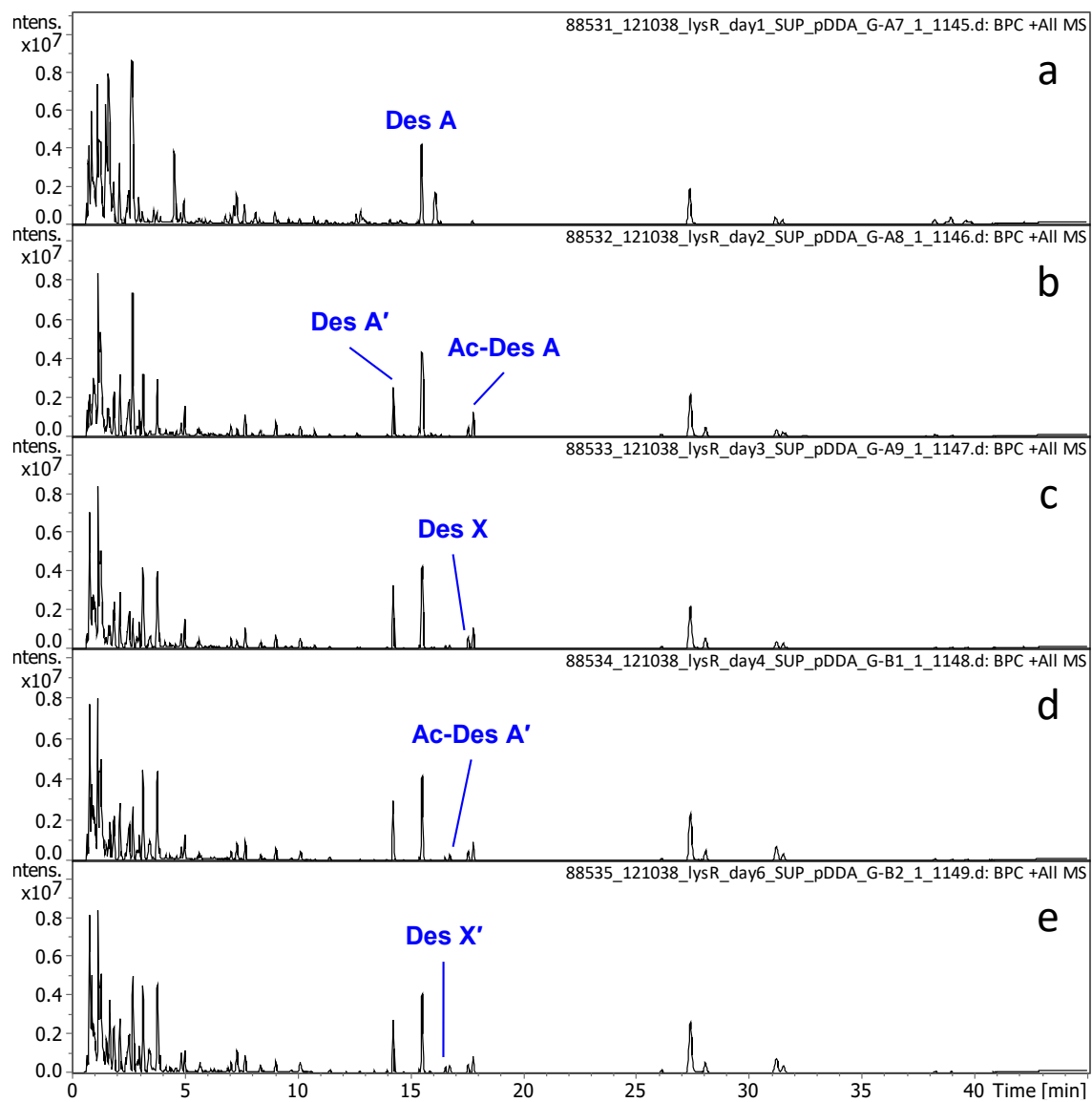

**FIG S4C**

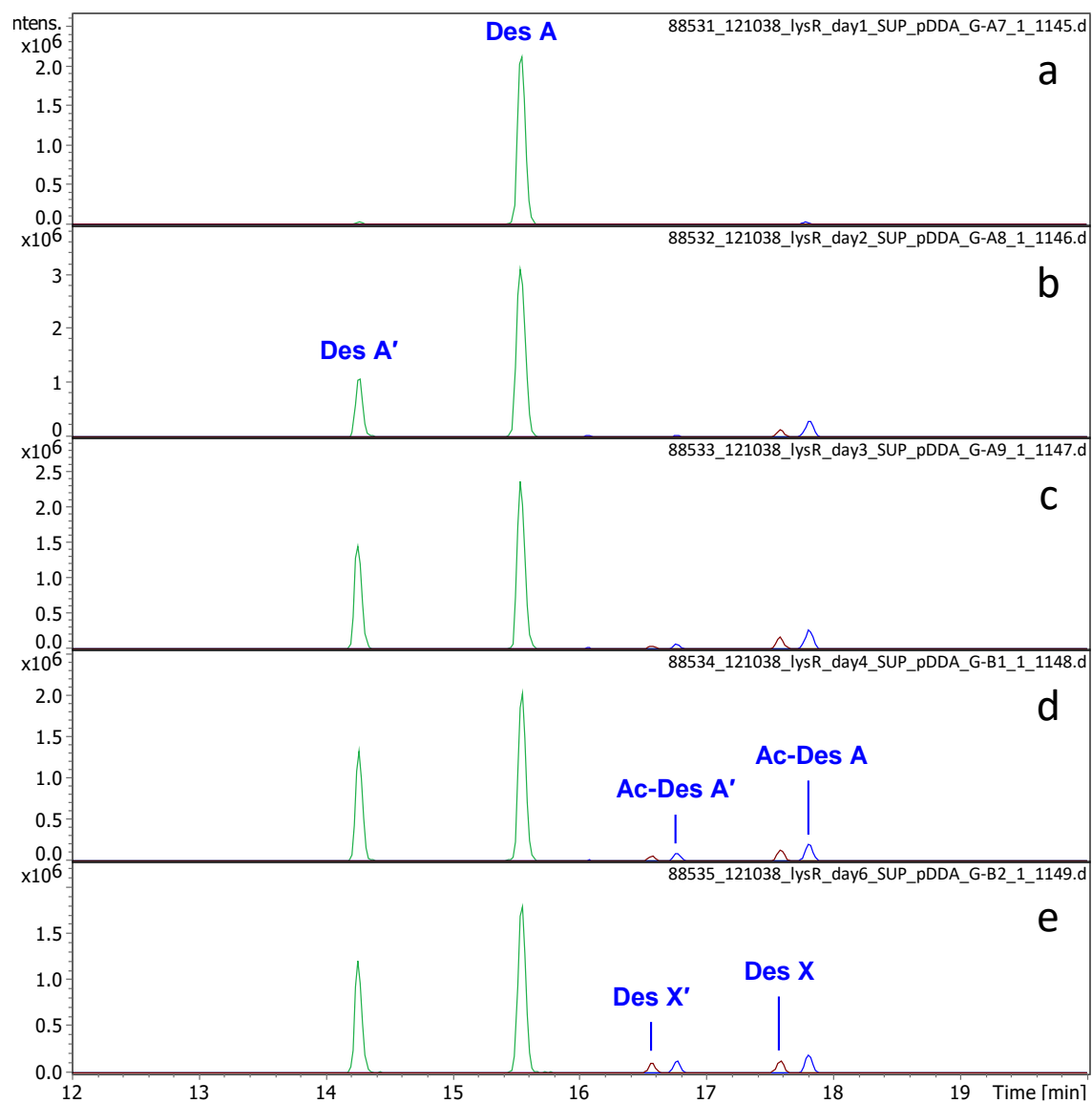

**FIG S4D**

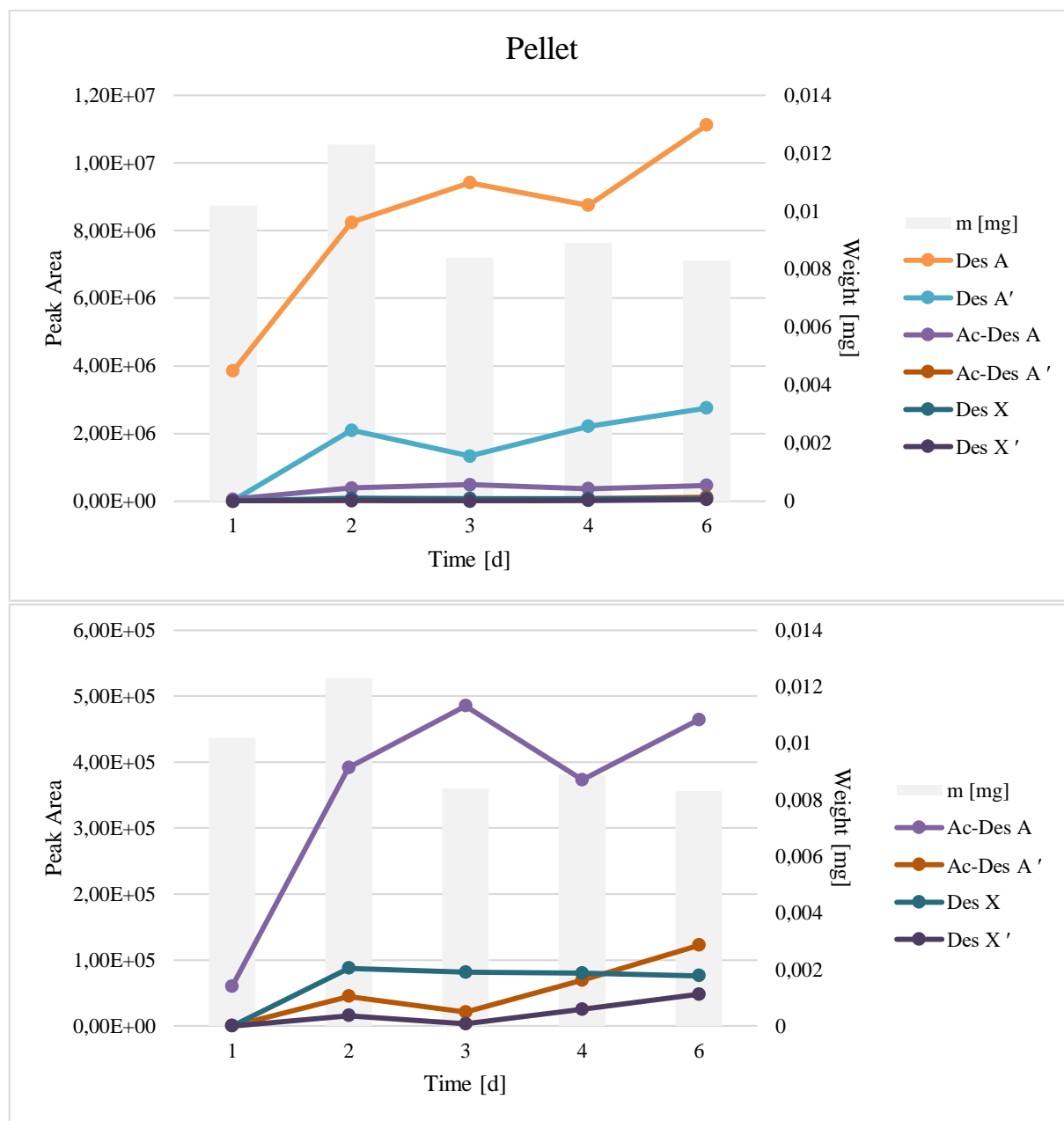

**FIG S4E**

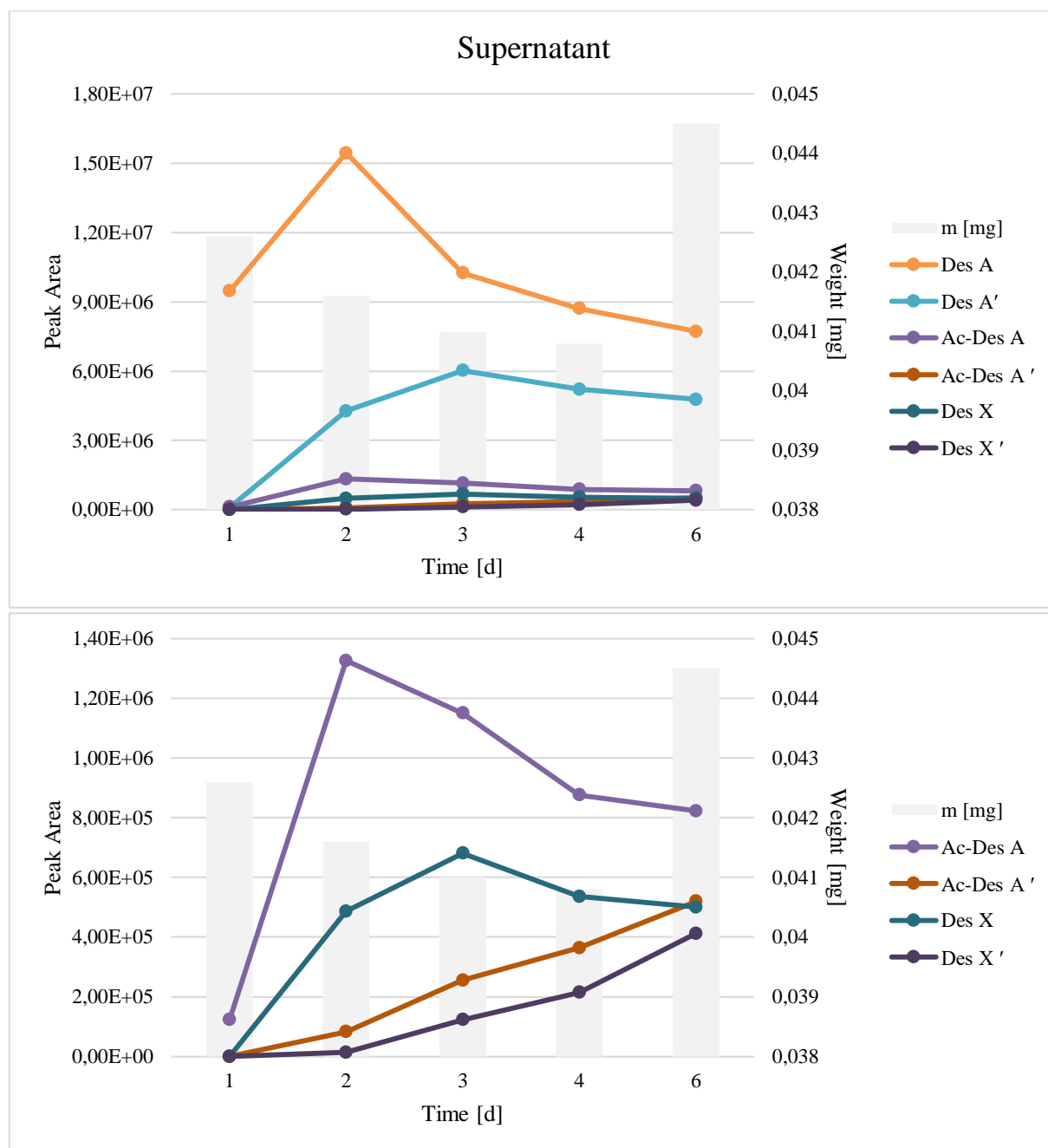

**FIG S4F**

Supplement: FIG S4 [file mbio.01789-22-s0008.pdf]

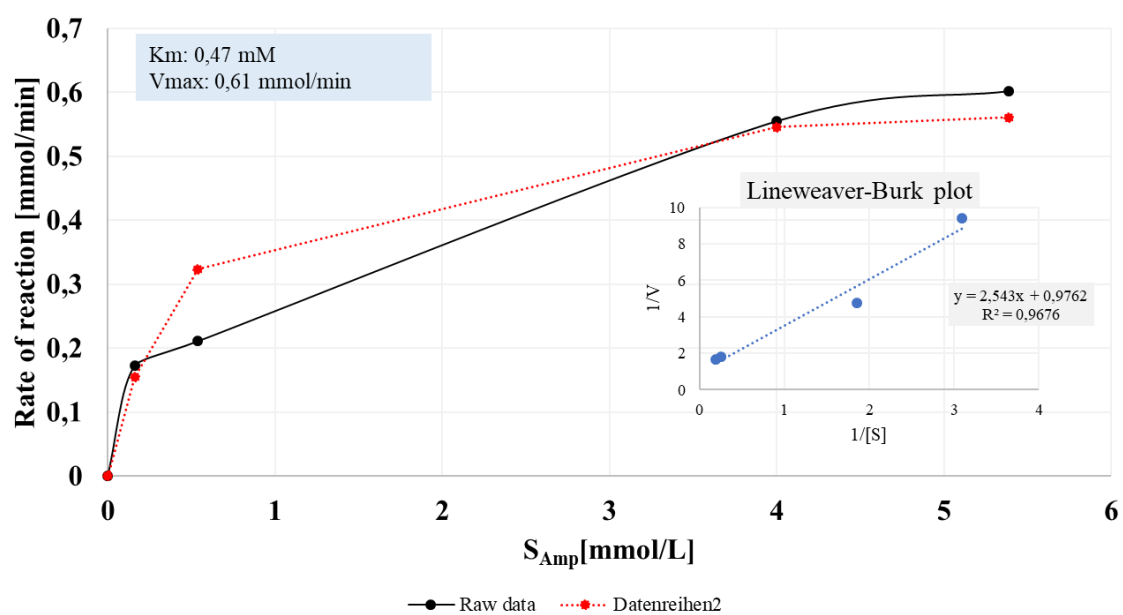

FIG S5

Supplement: FIG S5 [file mbio.01789-22-s0009.pdf]
