# Supplementary material for: N-Succinyltransferase Encoded by a Cryptic Siderophore Biosynthesis Gene Cluster in Streptomyces Modifies Structurally Distinct Antibiotics
Source: mBio. 2022 Aug 30;13(5):e01789-22. doi: 10.1128/mbio.01789-22 (PMC9600172; doi:10.1128/mbio.01789-22)
Supplement: TABLE S2 [file mbio.01789-22-s0002.docx]

**Table S2**

| Compound | MSSA  MB2865 | MRSA  MB5393 | *E. faecium*VR | *E. faecium*VS | *S. mutans* ATCC25175 |
| --- | --- | --- | --- | --- | --- |
|  | MIC, μg/mL | | | | |
| Aztreonam | >128 | >128 | >128 | >128 | NT |
| Ciprofloxacin | <0.25 | 0.5 | 16 | >128 | 4 |
| Gentamicin | 1 | <0.25 | 32 | >128 | NT |
| Imipenem | <0.25 | 8 | >128 | >128 | NT |
| Meropenem | <0.25 | 8 | >128 | >128 | NT |
| Penicillin G | 16 | 16 | >128 | >128 | NT |
| Rifampicin | 0.5 | 0.5 | 16 | 8 | NT |
| Vancomycin | 2 | 8 | >128 | 16 | NT |
| **Desertomycin A** | **32** | **64** | **128** | **64** | **32** |
| **Desertomycin X** | **>128** | **>128** | **>128** | **>128** | **>128** |

NT – not tested; MSSA - ; MRSA – Multiresistant *Staphylococcus aureus*; *E. faecium*VR - ;

*E. faecium*VS - ; *S. mutans* ATCC 25175 - .
