# Supplementary material for: N-Succinyltransferase Encoded by a Cryptic Siderophore Biosynthesis Gene Cluster in Streptomyces Modifies Structurally Distinct Antibiotics
Source: mBio. 2022 Aug 30;13(5):e01789-22. doi: 10.1128/mbio.01789-22 (PMC9600172; doi:10.1128/mbio.01789-22)
Supplement: FIG S3 [file mbio.01789-22-s0007.pdf]

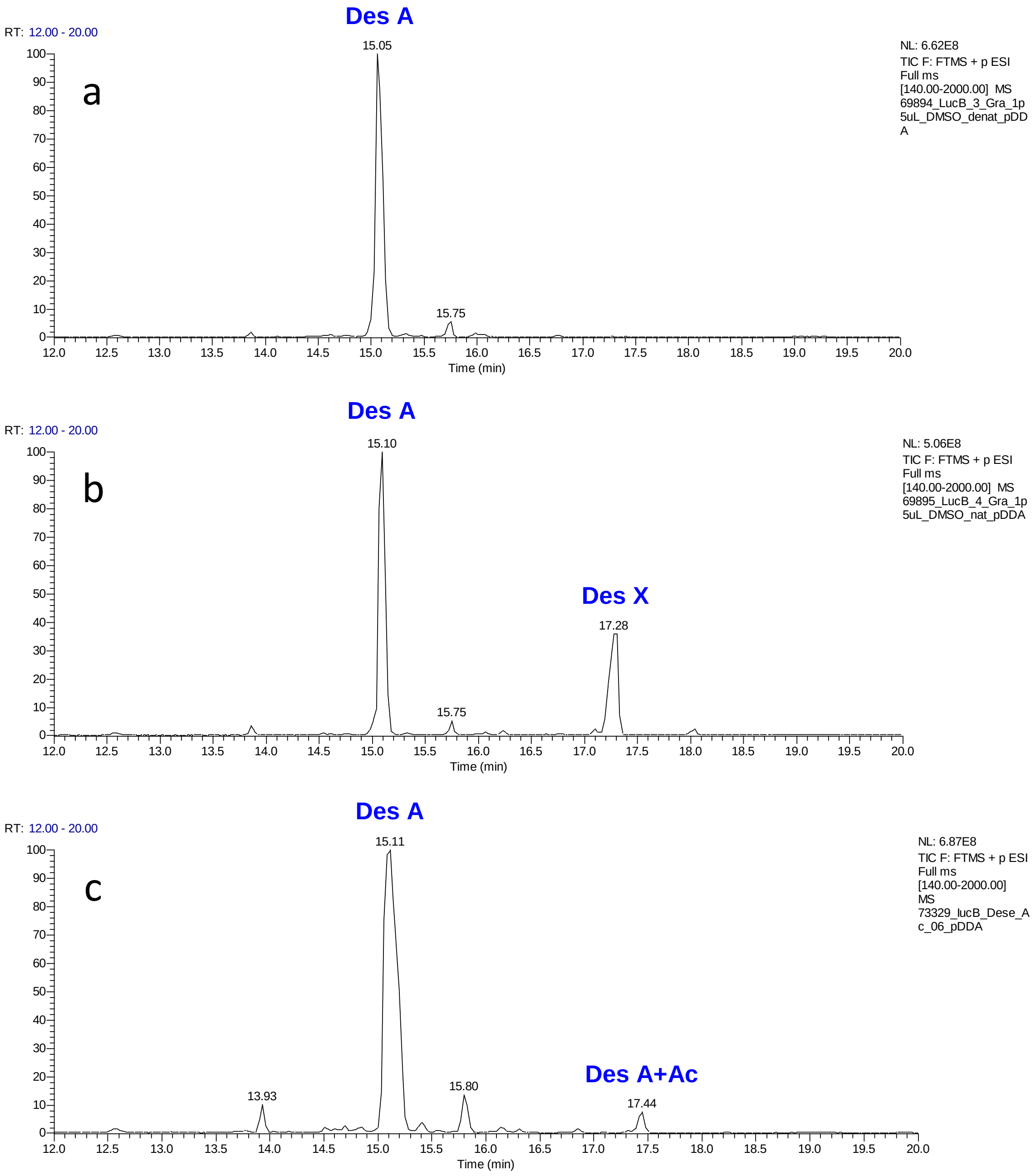

Des A
RT: 12.00 - 20.00
15.05
15.75
a
NL: 6.62E8
TIC F: FTMS + p ESI
Full ms
[140.00-2000.00] MS
69894_LucB_3_Gra_1p
5uL_DMSO_denat_pDD
A
Time (min)
Des A
RT: 12.00 - 20.00
15.10
b
Des X
17.28
15.75
NL: 5.06E8
TIC F: FTMS + p ESI
Full ms
[140.00-2000.00] MS
69895_LucB_4_Gra_1p
5uL_DMSO_nat_pDDA
Time (min)
Des A
RT: 12.00 - 20.00
15.11
c
13.93
15.80
Des A+Ac
17.44
NL: 6.87E8
TIC F: FTMS + p ESI
Full ms
[140.00-2000.00]
MS
73329_lucB_Dese_A
c_06_pDDA
Time (min)

FIG S3A

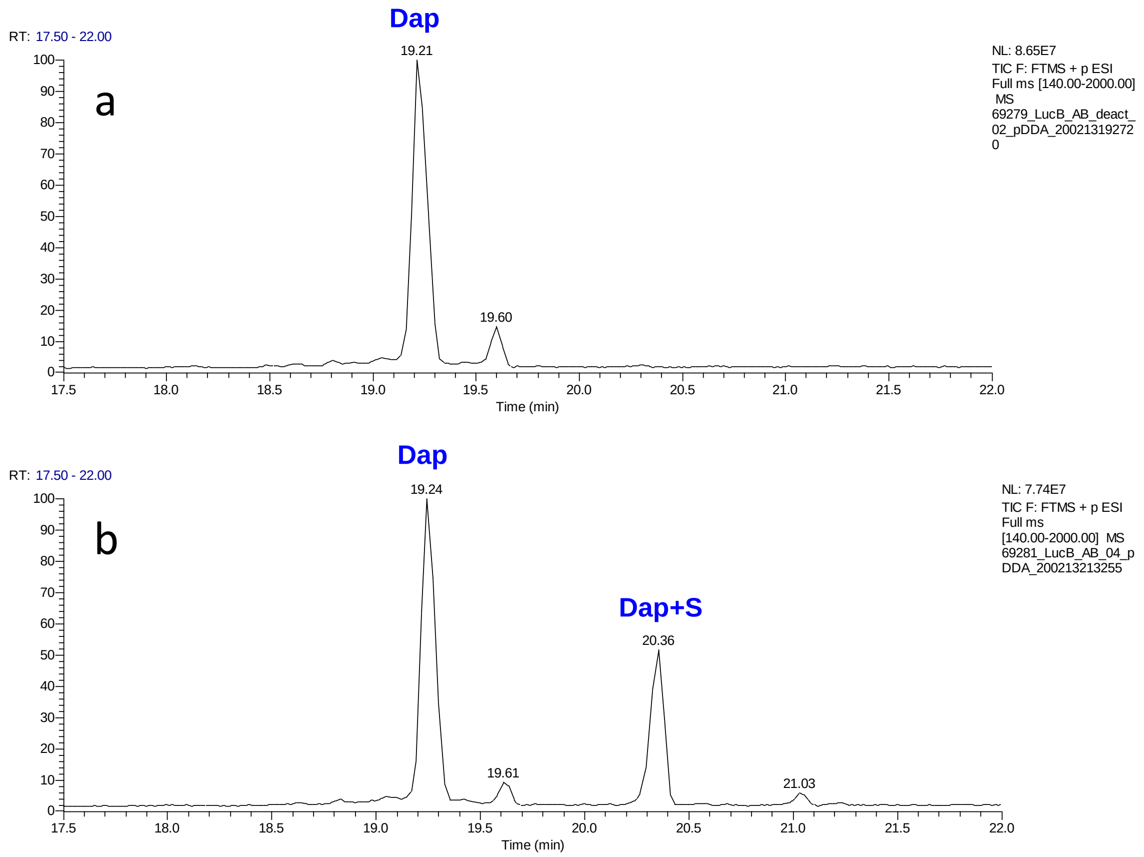

Dap
RT: 17.50 - 22.00
19.21
a
19.60
NL: 8.65E7
TIC F: FTMS + p ESI
Full ms [140.00-2000.00]
MS
69279_LucB_AB_deact_
02_pDDA_20021319272
0
Time (min)
Dap
RT: 17.50 - 22.00
19.24
b
Dap+S
20.36
19.61
21.03
NL: 7.74E7
TIC F: FTMS + p ESI
Full ms
[140.00-2000.00] MS
69281_LucB_AB_04_p
DDA_200213213255
Time (min)

FIG S3B

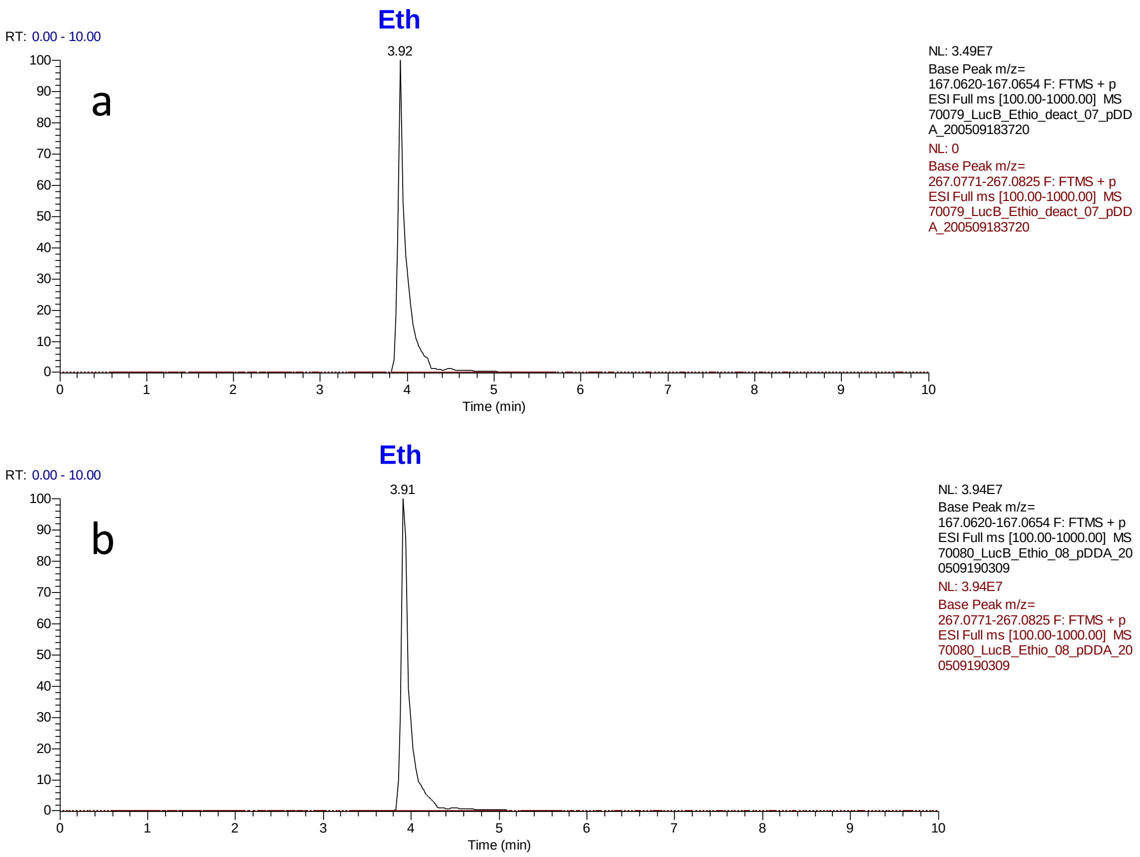

Eth
RT: 0.00 - 10.00
3.92
a
NL: 3.49E7
Base Peak m/z=
167.0620-167.0654 F: FTMS + p
ESI Full ms [100.00-1000.00] MS
70079_LucB_Ethio_deact_07_pDD
A_200509183720
NL: 0
Base Peak m/z=
267.0771-267.0825 F: FTMS + p
ESI Full ms [100.00-1000.00] MS
70079_LucB_Ethio_deact_07_pDD
A_200509183720
Time (min)
Eth
RT: 0.00 - 10.00
3.91
b
NL: 3.94E7
Base Peak m/z=
167.0620-167.0654 F: FTMS + p
ESI Full ms [100.00-1000.00] MS
70080_LucB_Ethio_08_pDDA_20
0509190309
NL: 3.94E7
Base Peak m/z=
267.0771-267.0825 F: FTMS + p
ESI Full ms [100.00-1000.00] MS
70080_LucB_Ethio_08_pDDA_20
0509190309
Time (min)

FIG S3C

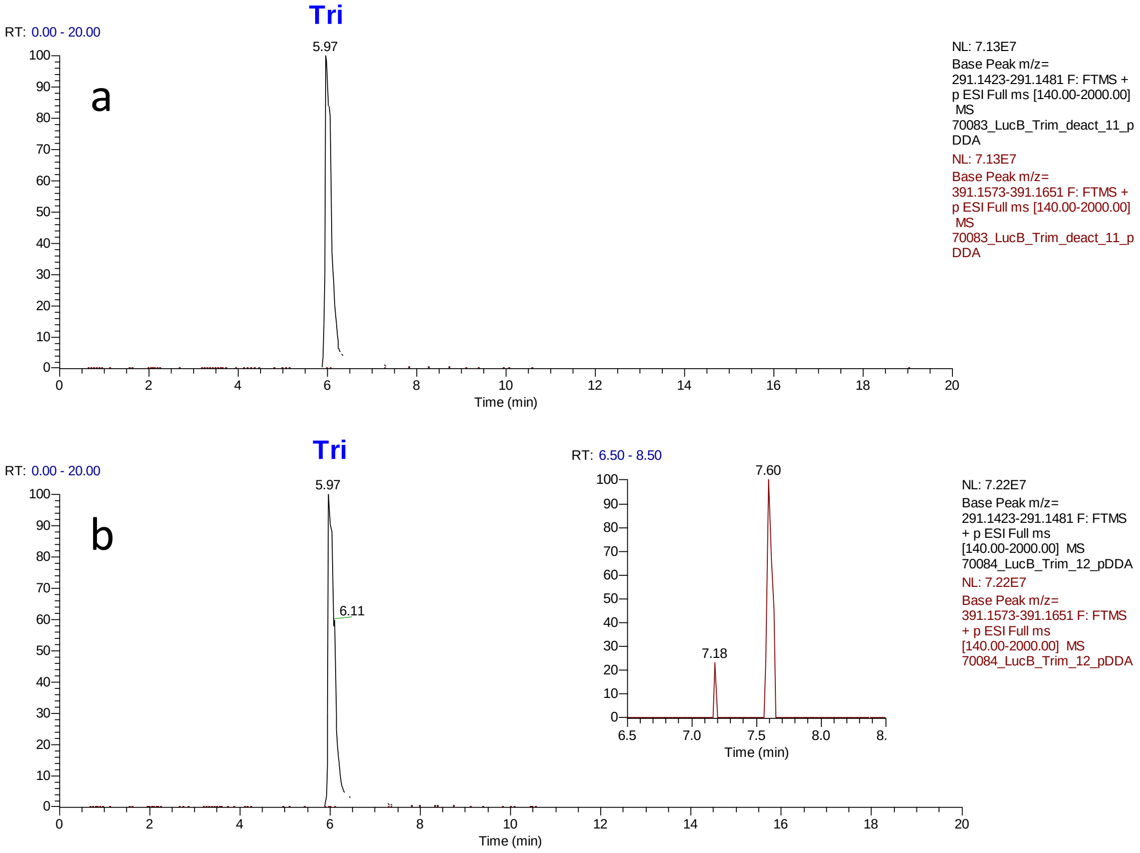
Tri
RT: 0.00 - 20.00
a
5.97
NL: 7.13E7
Base Peak m/z= 291.1423-291.1481 F: FTMS + p ESI Full ms [140.00-2000.00] MS 70083_LucB_Trim_deact_11_pDDA
NL: 7.13E7
Base Peak m/z= 391.1573-391.1651 F: FTMS + p ESI Full ms [140.00-2000.00] MS 70083_LucB_Trim_deact_11_pDDA
Time (min)
Tri
RT: 0.00 - 20.00
b
5.97
6.11
RT: 6.50 - 8.50
7.60
7.18
Time (min)
NL: 7.22E7
Base Peak m/z= 291.1423-291.1481 F: FTMS + p ESI Full ms [140.00-2000.00] MS 70084_LucB_Trim_12_pDDA
NL: 7.22E7
Base Peak m/z= 391.1573-391.1651 F: FTMS + p ESI Full ms [140.00-2000.00] MS 70084_LucB_Trim_12_pDDA
Time (min)

FIG S3D

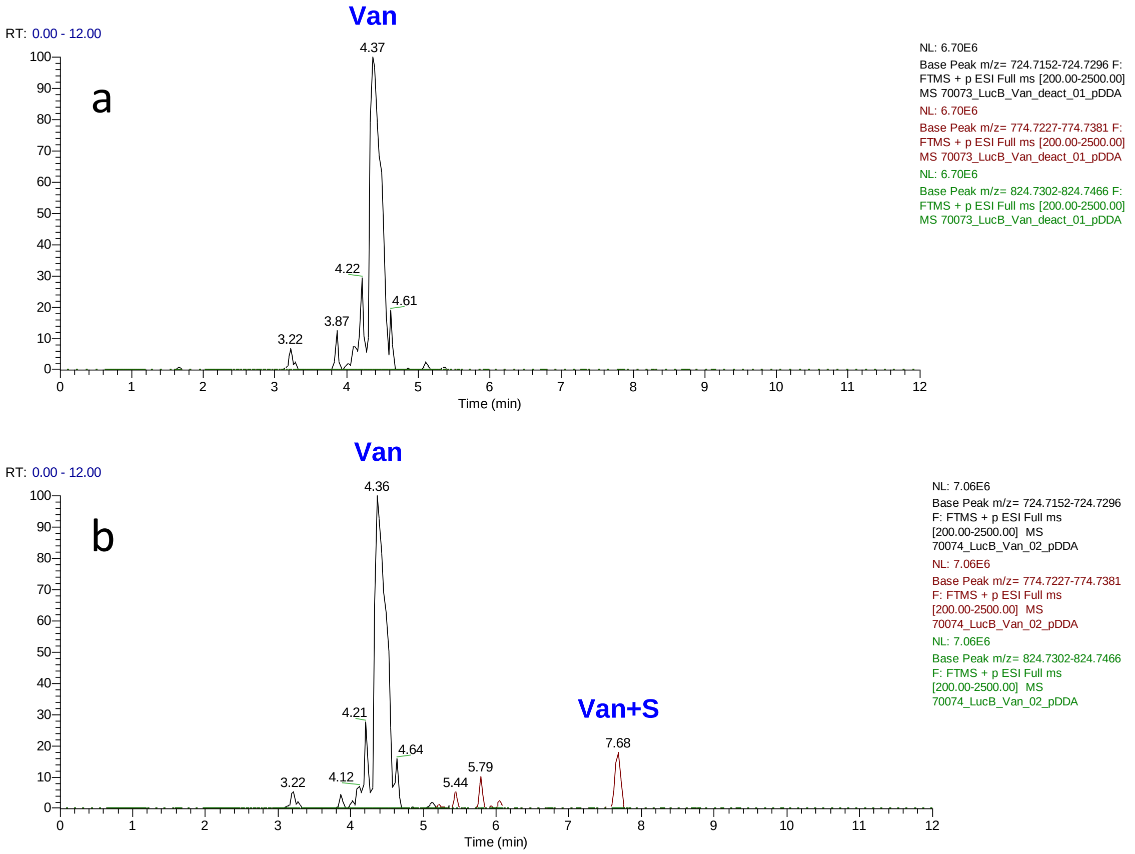

Van
RT: 0.00 - 12.00
a
4.37
4.22
4.61
3.87
3.22
Time (min)
NL: 6.70E6
Base Peak m/z= 724.7152-724.7296 F: FTMS + p ESI Full ms [200.00-2500.00] MS 70073_LucB_Van_deact_01_pDDA
NL: 6.70E6
Base Peak m/z= 774.7227-774.7381 F: FTMS + p ESI Full ms [200.00-2500.00] MS 70073_LucB_Van_deact_01_pDDA
NL: 6.70E6
Base Peak m/z= 824.7302-824.7466 F: FTMS + p ESI Full ms [200.00-2500.00] MS 70073_LucB_Van_deact_01_pDDA
Van
RT: 0.00 - 12.00
b
4.36
4.21
4.64
4.12
3.22
5.44
5.79
Van+S
7.68
Time (min)
NL: 7.06E6
Base Peak m/z= 724.7152-724.7296 F: FTMS + p ESI Full ms [200.00-2500.00] MS 70074_LucB_Van_02_pDDA
NL: 7.06E6
Base Peak m/z= 774.7227-774.7381 F: FTMS + p ESI Full ms [200.00-2500.00] MS 70074_LucB_Van_02_pDDA
NL: 7.06E6
Base Peak m/z= 824.7302-824.7466 F: FTMS + p ESI Full ms [200.00-2500.00] MS 70074_LucB_Van_02_pDDA

FIG S3E

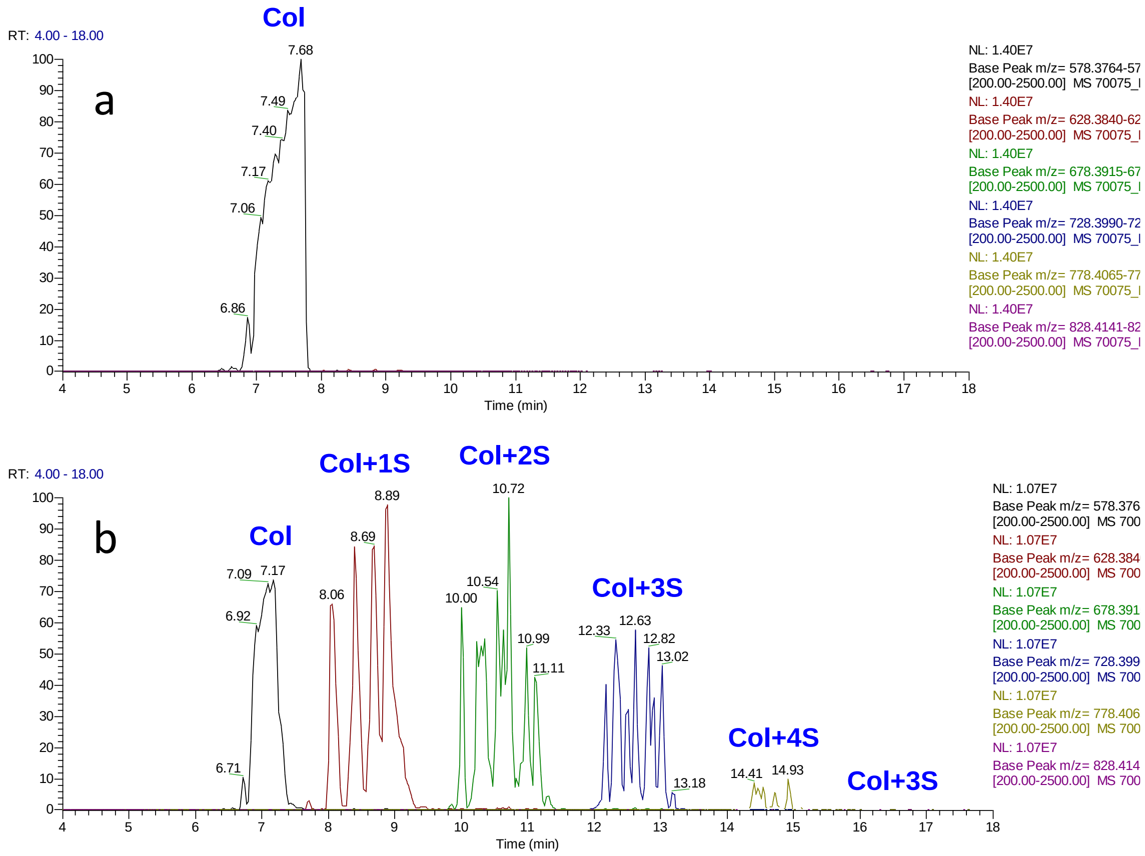

Col
RT: 4.00 - 18.00
a
7.68
7.49
7.40
7.17
7.06
6.86
NL: 1.40E7
Base Peak m/z= 578.3764-57
[200.00-2500.00] MS 70075_I
NL: 1.40E7
Base Peak m/z= 628.3840-62
[200.00-2500.00] MS 70075_I
NL: 1.40E7
Base Peak m/z= 678.3915-67
[200.00-2500.00] MS 70075_I
NL: 1.40E7
Base Peak m/z= 728.3990-72
[200.00-2500.00] MS 70075_I
NL: 1.40E7
Base Peak m/z= 778.4065-77
[200.00-2500.00] MS 70075_I
NL: 1.40E7
Base Peak m/z= 828.4141-82
[200.00-2500.00] MS 70075_I
Time (min)
Col+1S
Col+2S
RT: 4.00 - 18.00
b
Col
Col+3S
Col+4S
Col+3S
NL: 1.07E7
Base Peak m/z= 578.376
[200.00-2500.00] MS 700
NL: 1.07E7
Base Peak m/z= 628.384
[200.00-2500.00] MS 700
NL: 1.07E7
Base Peak m/z= 678.391
[200.00-2500.00] MS 700
NL: 1.07E7
Base Peak m/z= 728.399
[200.00-2500.00] MS 700
NL: 1.07E7
Base Peak m/z= 778.406
[200.00-2500.00] MS 700
NL: 1.07E7
Base Peak m/z= 828.414
[200.00-2500.00] MS 700
Time (min)

FIG S3F

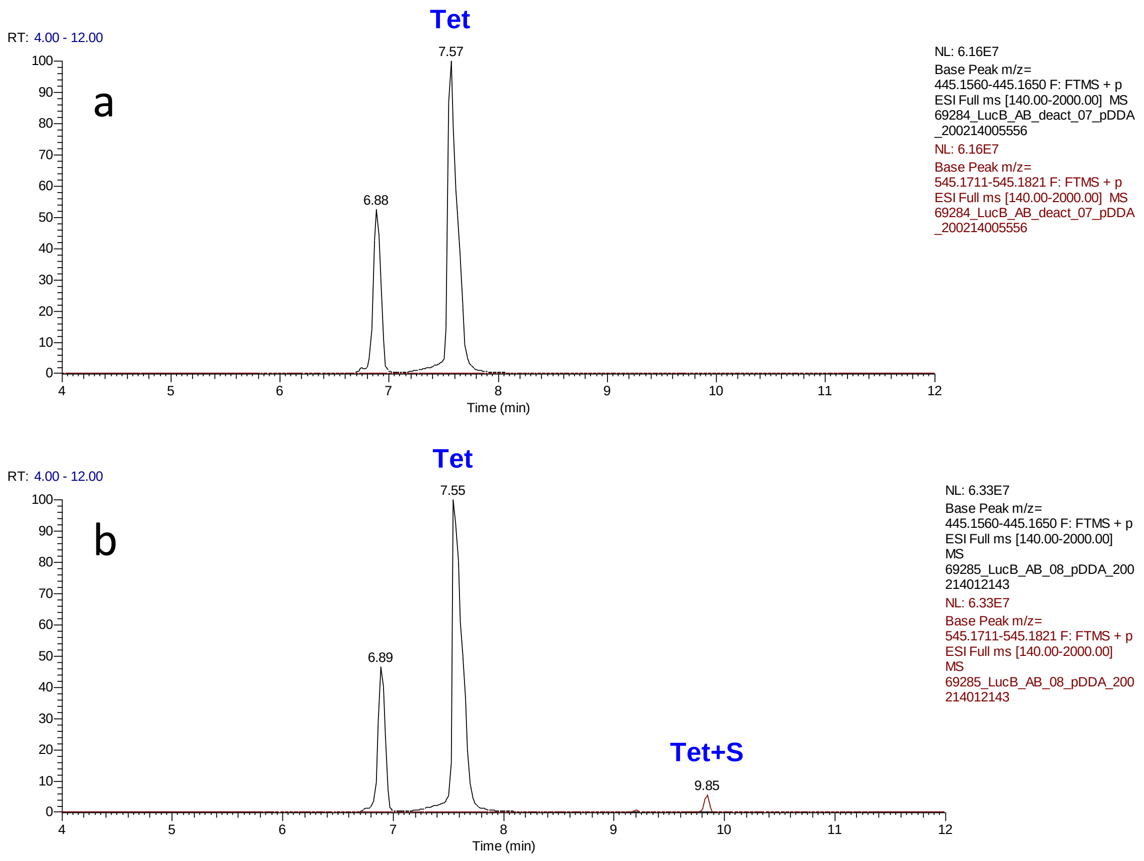

Tet
RT: 4.00 - 12.00
a
7.57
6.88
NL: 6.16E7
Base Peak m/z=
445.1560-445.1650 F: FTMS + p
ESI Full ms [140.00-2000.00] MS
69284_LucB_AB_deact_07_pDDA
_200214005556
NL: 6.16E7
Base Peak m/z=
545.1711-545.1821 F: FTMS + p
ESI Full ms [140.00-2000.00] MS
69284_LucB_AB_deact_07_pDDA
_200214005556
Time (min)
Tet
RT: 4.00 - 12.00
b
7.55
6.89
Tet+S
9.85
NL: 6.33E7
Base Peak m/z=
445.1560-445.1650 F: FTMS + p
ESI Full ms [140.00-2000.00]
MS
69285_LucB_AB_08_pDDA_200
214012143
NL: 6.33E7
Base Peak m/z=
545.1711-545.1821 F: FTMS + p
ESI Full ms [140.00-2000.00]
MS
69285_LucB_AB_08_pDDA_200
214012143
Time (min)

FIG S3G

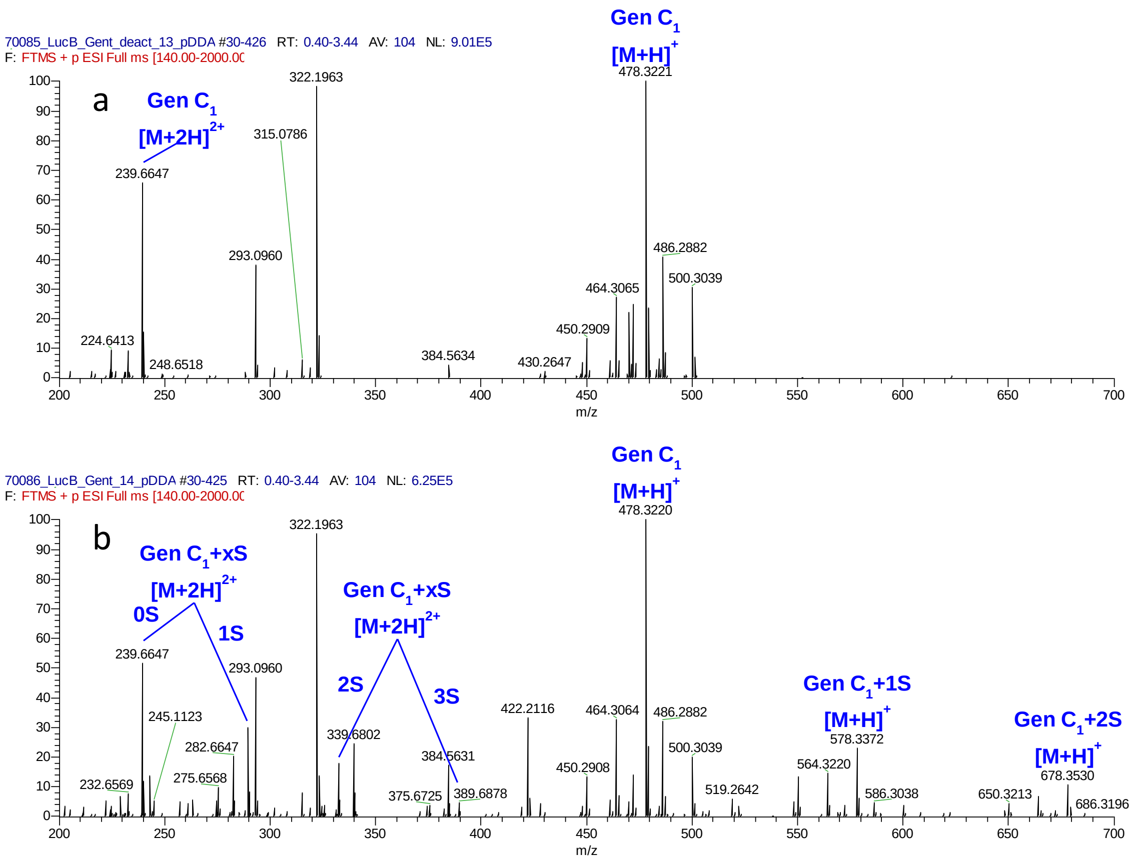

70085_LucB_Gent_deact_13_pDDA #30-426 RT: 0.40-3.44 AV: 104 NL: 9.01E5
F: FTMS + p ESI Full ms [140.00-2000.00
a
Gen C1 [M+2H]2+
Gen C1 [M+H]+
478.3221
322.1963
315.0786
239.6647
293.0960
486.2882
500.3039
464.3065
450.2909
224.6413
248.6518
384.5634
430.2647
m/z
70086_LucB_Gent_14_pDDA #30-425 RT: 0.40-3.44 AV: 104 NL: 6.25E5
F: FTMS + p ESI Full ms [140.00-2000.00
b
Gen C1+xS [M+2H]2+
0S
1S
2S
3S
Gen C1 [M+H]+
478.3220
322.1963
239.6647
293.0960
245.1123
282.6647
275.6568
232.6569
339.6802
384.5631
375.6725
389.6878
422.2116
464.3064
486.2882
500.3039
450.2908
519.2642
Gen C1+1S [M+H]+
578.3372
564.3220
586.3038
Gen C1+2S [M+H]+
678.3530
650.3213
686.3196
m/z

FIG S3H

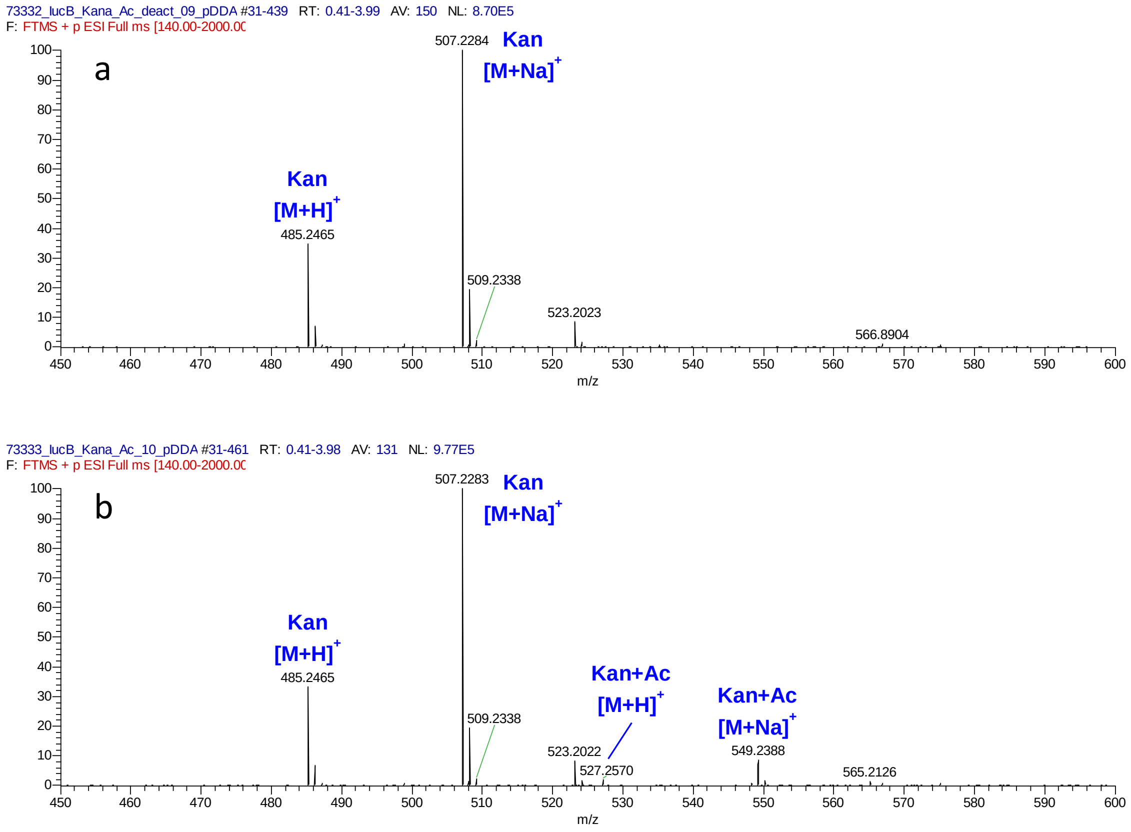

73332_lucB_Kana_Ac_deact_09_pDDA #31-439 RT: 0.41-3.99 AV: 150 NL: 8.70E5
F: FTMS + p ESI Full ms [140.00-2000.00
a
Kan
[M+Na]+
507.2284
Kan
[M+H]+
485.2465
509.2338
523.2023
566.8904
m/z
73333_lucB_Kana_Ac_10_pDDA #31-461 RT: 0.41-3.98 AV: 131 NL: 9.77E5
F: FTMS + p ESI Full ms [140.00-2000.00
b
Kan
[M+Na]+
507.2283
Kan
[M+H]+
485.2465
509.2338
Kan+Ac
[M+H]+
523.2022
527.2570
Kan+Ac
[M+Na]+
549.2388
565.2126
m/z

FIG S3I

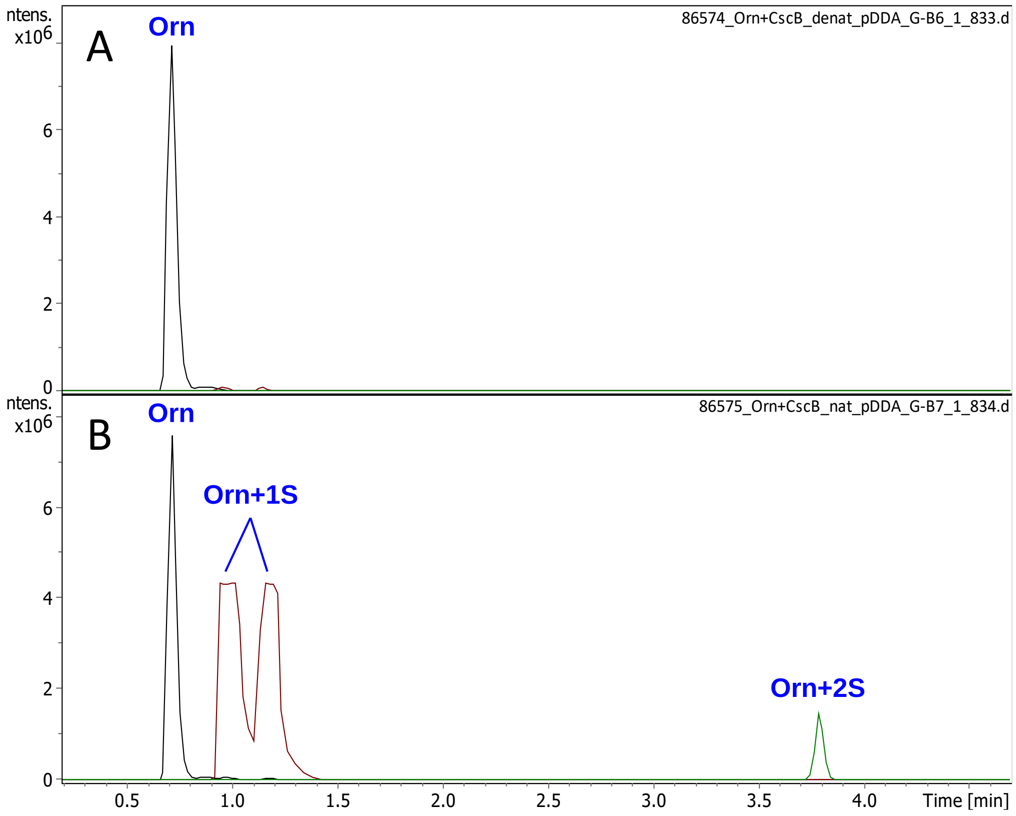

Intens.
x10^6
A
Orn
86574_Orn+CscB_denat_pDDA_G-B6_1_833.d
0
2
4
6
Intens.
x10^6
B
Orn
Orn+1S
Orn+2S
86575_Orn+CscB_nat_pDDA_G-B7_1_834.d
0
2
4
6
0.5
1.0
1.5
2.0
2.5
3.0
3.5
4.0
Time [min]

FIG S3J

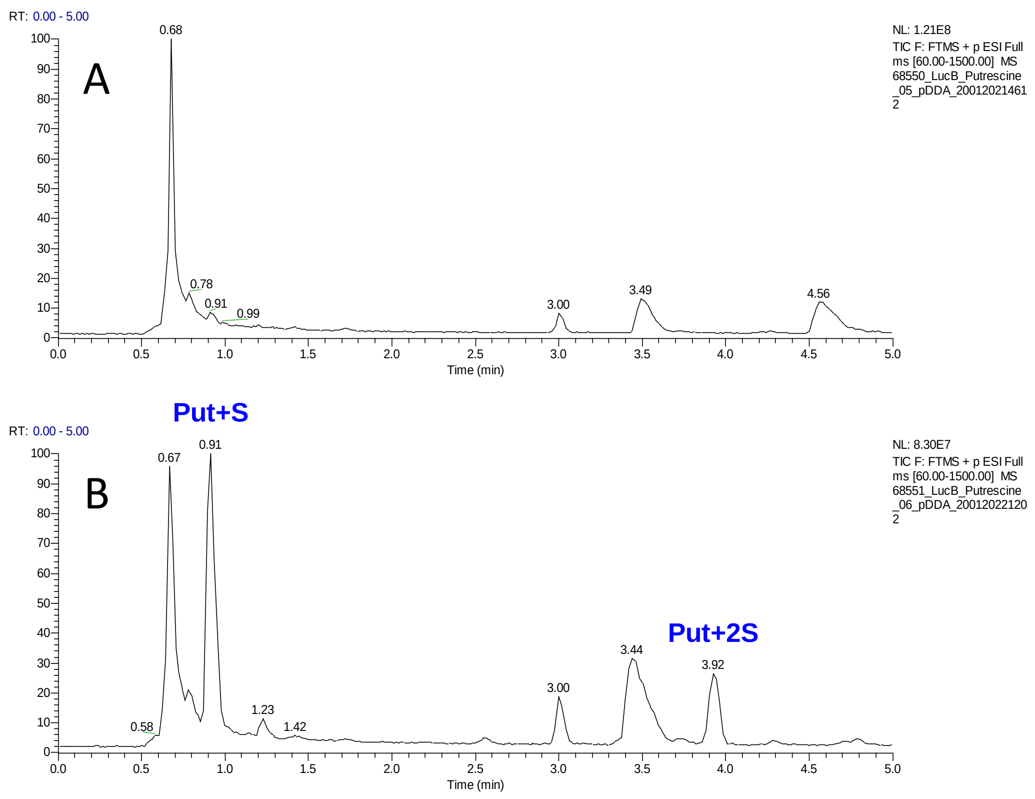

RT: 0.00 - 5.00
A
0.68
0.78
0.91
0.99
3.00
3.49
4.56
NL: 1.21E8
TIC F: FTMS + p ESI Full ms [60.00-1500.00] MS 68550_LucB_Putrescine_05_pDDA_200120214612
Time (min)
RT: 0.00 - 5.00
Put+S
B
0.67
0.91
0.58
1.23
1.42
3.00
Put+2S
3.44
3.92
NL: 8.30E7
TIC F: FTMS + p ESI Full ms [60.00-1500.00] MS 68551_LucB_Putrescine_06_pDDA_200120221202
Time (min)

FIG S3K
