# Supplementary material for: N-Succinyltransferase Encoded by a Cryptic Siderophore Biosynthesis Gene Cluster in Streptomyces Modifies Structurally Distinct Antibiotics
Source: mBio. 2022 Aug 30;13(5):e01789-22. doi: 10.1128/mbio.01789-22 (PMC9600172; doi:10.1128/mbio.01789-22)
Supplement: TABLE S3 [file mbio.01789-22-s0003.docx]

**Table S3A.**

| DstA3_*Hind*III | CGATAAG*AAGCTT*GTCATGAACGCCACGTACAACGG |
| --- | --- |
| DstA3_*EcoR*I | GAAAGCG*AATTC*CTCGCGCTCCGCCTCGCGCAGGTG |
| DstA3_fwd | TACATGAAGGCGTGGCGGAAC |
| DstA3_rev | ATGGTGCTTCCGACGGAAGAC |
| pGEM_Amp_fwd | GATGCATGGTTACTCACCACTGCGATCCCCACTTTTCGGGGAAATGTG |
| pGEM_Amp_rev | AACCCTATCTCGGTCTATTCTTTTGATTTATAAACTTGGTCTGACAGTTAC |
| CsbCwt_*Mfe*I | CATC*CAATTG*TCGGGCGGTGTACGAGAAG |
| CsbCwt_*Hind*III | GCGCC*AAGCTT*CATGCCCGCCATGACCCAC |

**Table S3B.**

| **Plasmid** | **Description** | **Source** |  |  |
| --- | --- | --- | --- | --- |
| pSOK201(2) | pSG5 minimal replicon, Am^R^, RP4 oriT, ColEI replication origin |  |  |  |
| pET-30a(+) | Protein expressing vector; Kan^R^ | Novagen |  |  |
| pGEM-3Zf(+) | Standard cloning vector; ColEI replicon, Amp^R^ | Promega |  |  |
| pUWLoriT(3) | Replicative vector for actinomycetes; pIJ101 replicon, *oriT*, Thio^R^, Amp^R^, *ermE* |  |  |  |
| pET-CsbC-S | *csb-S* expression, Kan^R^, based on pET-30a(+) | This work |  |  |
| pA | Amp^R^, based on pET-30a(+) | This work |  |  |
| pA-CsbC-S | *csb-S* expression, Amp^R^, based on pET-30a(+) | This work |  |  |
| pUWL_CsbCwt | *csbC* wild type expression, based on pUWLoriT | This work |  |  |
| **Bacterial strain** | **Description** | **Source** |  |  |
| *E. coli* XL1 Blue | General cloning host | New England Biolabs |  |  |
| *E. coli* ET12567/pUZ8002(4) | Strain for intergenic conjugation; Km^R^, Cm^R^ |  |  |  |
| *E. coli* BL21 (DE3) | CsbC expressing host | New England Biolabs |  |  |
| *Streptomyces sp*. YIM 120138 | Wild-type | This work |  |  |
| *Streptomyces sp*. YIM 121038/KN | Wild-type with integrated pDstA3_KN vector for *dstA3* gene disruption | This work |  |  |
|  |  |  |  |  |
