# Supplementary material for: N-Succinyltransferase Encoded by a Cryptic Siderophore Biosynthesis Gene Cluster in Streptomyces Modifies Structurally Distinct Antibiotics
Source: mBio. 2022 Aug 30;13(5):e01789-22. doi: 10.1128/mbio.01789-22 (PMC9600172; doi:10.1128/mbio.01789-22)
Supplement: TABLE S4 [file mbio.01789-22-s0004.docx]

**Table S4A.**

| Desertomycin X(**1**) | | | | | |  |
| --- | --- | --- | --- | --- | --- | --- |
| No. | δ_C_ | δ_H_, mult. | No. | δ_C_ | δ_H_, mult. |  |
| 1 | 169.2 |  | 34 | 42.5 | 1.66 ovlp. |  |
| 2 | 129.0 |  |  |  | 1.45 ovlp. |  |
| 3 | 144.1 | 6.79 (t, 7.1) | 35 | 66.3 | 4.03 ovlp. |  |
| 4 | 27.5 | 2.26 ovlp. | 36 | 46.3 | 1.54 ovlp. |  |
| 5 | 34.4 | 1.57 ovlp. | 37 | 69.7 | 4.27 m |  |
|  |  | 1.43 ovlp. | 38 | 137.9 | 5.56 (dd, 5.3, 15.7) |  |
| 6 | 35.9 | 1.67 ovlp. | 39 | 126.0 | 5.63 (ddd, 6.7, 6.7, 15.7) |  |
| 7 | 77.4 | 3.40 brd | 40 | 34.2 | 2.44 ovlp. |  |
| 8 | 42.8 | 1.75 m |  |  | 2.30 ovlp. |  |
| 9 | 74.6 | 3.82 ovlp. | 41 | 75.7 | 5.14 m |  |
| 10 | 33.3 | 1.57 ovlp. | 42 | 43.5 | 1.97 m |  |
|  |  | 1.39 ovlp. | 43 | 72.7 | 3.51 m |  |
| 11 | 30.4 | 2.25 ovlp. | 44 | 31.2 | 1.56 ovlp. |  |
|  |  | 2.07 m |  |  | 1.33 ovlp. |  |
| 12 | 131.6 | 5.48 ovlp. | 45 | 27.0 | 1.69 ovlp. |  |
| 13 | 133.9 | 5.46 ovlp. |  |  | 1.50 ovlp. |  |
| 14 | 44.0 | 2.20 m | 46 | 40.2 | 3.15 m |  |
| 15 | 76.6 | 3.88 (dd, 5.0, 5.0) | 47 | 12.6 | 1.85 brs |  |
| 16 | 131.9 | 5.48 ovlp. | 48 | 12.5 | 0.89 (d, 6.7) |  |
| 17 | 134.6 | 5.53 ovlp. | 49 | 11.9 | 0.78 (d, 6.7) |  |
| 18 | 41.1 | 2.33 m | 50 | 16.0 | 0.98 (d, 6.9) |  |
| 19 | 83.3 | 3.72 (d, 8.8) | 51 | 17.5 | 1.11 (d, 6.6) |  |
| 20 | 145.5 |  | 52 | 12.2 | 1.71 brs |  |
| 21 | 124.0 | 5.30 (d, 9.3) | 53 | 10.0 | 0.94 (d, 7.1) |  |
| 22 | 75.6 | 4.40 (dd, 3.4, 9.5) | 54 | 11.5 | 0.79 (d, 6.8) |  |
| 23 | 71.6 | 3.97 m | 55 | 10.9 | 0.93 (d, 7.3) |  |
| 24 | 41.4 | 1.51 ovlp. | 1' | 97.7 | 4.84 ovlp. |  |
| 25 | 65.9 | 4.09 m | 2' | 72.3 | 3.76 ovlp. |  |
| 26 | 46.3 | 1.64 ovlp. | 3' | 72.5 | 3.79 ovlp. |  |
|  |  | 1.54 ovlp. | 4' | 68.7 | 3.64 ovlp. |  |
| 27 | 69.0 | 4.07 m | 5' | 74.6 | 3.64 ovlp. |  |
| 28 | 43.1 | 1.74 ovlp. | 6' | 62.9 | 3.85 m |  |
|  |  | 1.67 ovlp. |  |  | 3.73 ovlp. |  |
| 29 | 74.9 | 3.81 ovlp. | 1'' | 175.7 |  |  |
| 30 | 40.8 | 1.64 ovlp. | 2'' | 34.1 | 2.45 ovlp. |  |
| 31 | 73.5 | 3.99 ovlp. | 3'' | 34.1 | 2.45 ovlp. |  |
| 32 | 41.7 | 1.69 ovlp. | 4'' | 180.1 |  |  |
| 33 | 70.1 | 4.17 m |  |  |  |  |
|  |  |  |  |  |  |  |

**Table S4B**

| Desertomycin A(**2**) | | | | | |  |
| --- | --- | --- | --- | --- | --- | --- |
| No. | δ_C_ | δ_H_, mult. | No. | δ_C_ | δ_H_, mult. |  |
| 1 | 169.2 |  | 34 | 42.5 | 1.64 ovlp. |  |
| 2 | 128.8 |  |  |  | 1.44 m |  |
| 3 | 144.3 | 6.80 (t, 7.4) | 35 | 66.3 | 4.03 ovlp. |  |
| 4 | 27.6 | 2.25 ovlp. | 36 | 46.3 | 1.53 ovlp. |  |
| 5 | 34.4 | 1.57 ovlp. | 37 | 69.6 | 4.27 m |  |
|  |  | 1.42 ovlp. | 38 | 138.1 | 5.57 (dd, 5.3, 15.3) |  |
| 6 | 35.9 | 1.66 ovlp. | 39 | 125.7 | 5.63 (ddd, 6.7, 6.7, 15.3) |  |
| 7 | 77.5 | 3.40 brd | 40 | 34.4 | 2.45 br |  |
| 8 | 42.9 | 1.75 m |  |  | 2.30 ovlp. |  |
| 9 | 74.7 | 3.81 ovlp. | 41 | 75.6 | 5.10 m |  |
| 10 | 33.4 | 1.59 ovlp. | 42 | 43.6 | 1.99 m |  |
|  |  | 1.39 ovlp. | 43 | 72.5 | 3.53 m |  |
| 11 | 30.3 | 2.24 ovlp. | 44 | 30.4 | 1.63 ovlp. |  |
|  |  | 2.06 m |  |  | 1.39 ovlp. |  |
| 12 | 131.6 | 5.47 ovlp. | 45 | 25.9 | 1.81 ovlp. |  |
| 13 | 133.9 | 5.44 ovlp. |  |  | 1.64 ovlp. |  |
| 14 | 44.0 | 2.20 m | 46 | 40.9 | 2.90 m |  |
| 15 | 76.7 | 3.88 (dd, 5.3, 5.3) | 47 | 12.7 | 1.85 brs |  |
| 16 | 131.9 | 5.49 ovlp. | 48 | 12.5 | 0.89 (d, 6.7) |  |
| 17 | 134.5 | 5.50 ovlp. | 49 | 12.0 | 0.78 (d, 6.4) |  |
| 18 | 41.1 | 2.33 m | 50 | 16.1 | 0.98 (d, 6.8) |  |
| 19 | 83.4 | 3.72 (d, 8.6) | 51 | 17.6 | 1.11 (d, 6.6) |  |
| 20 | 145.5 |  | 52 | 12.2 | 1.71 brs |  |
| 21 | 124.0 | 5.30 (d, 9.6) | 53 | 10.1 | 0.94 (d, 7.0) |  |
| 22 | 75.6 | 4.40 (dd, 3.4, 9.6) | 54 | 11.5 | 0.79 (d, 6.7) |  |
| 23 | 71.6 | 3.97 m | 55 | 10.1 | 0.94 (d, 7.0) |  |
| 24 | 41.4 | 1.50 ovlp. | 1' | 97.8 | 4.83 brs |  |
| 25 | 65.9 | 4.09 m | 2' | 72.4 | 3.76 ovlp. |  |
| 26 | 46.3 | 1.61 ovlp. | 3' | 72.6 | 3.78 ovlp. |  |
|  |  | 1.53 ovlp. | 4' | 68.7 | 3.63 ovlp. |  |
| 27 | 69.0 | 4.06 m | 5' | 74.6 | 3.63 ovlp. |  |
| 28 | 43.1 | 1.72 ovlp. | 6' | 62.9 | 3.85 brd |  |
|  |  |  |  |  | 3.73 ovlp. |  |
| 29 | 74.7 | 3.81 ovlp. |  |  |  |  |
| 30 | 40.8 | 1.63 ovlp. |  |  |  |  |
| 31 | 73.4 | 3.99 ovlp. |  |  |  |  |
| 32 | 41.7 | 1.69 ovlp. |  |  |  |  |
| 33 | 70.1 | 4.17 brdt |  |  |  |  |
|  |  |  |  |  |  |  |
